# Structure of Blm10:13S proteasome intermediate reveals parallel assembly pathways for the proteasome core particle

**DOI:** 10.1038/s41467-026-77943-w

**Published:** 2026-09-21

**Authors:** Mandeep Kaur, Xiang Chen, Stella Y. Lee, Tyler M. Weaver, Bret D. Freudenthal, Kylie J. Walters, Jeroen Roelofs

**Affiliations:** 1 Department of Biochemistry and Molecular Biology, University of Kansas Medical Center, 3901 Rainbow Blvd., HLSIC 1077, Kansas City, KS USA; 2 Protein Processing Section, Center for Structural Biology, Center for Cancer Research, National Cancer Institute, National Institutes of Health, Frederick, MD USA; 3 Present Address: Department of Biochemistry and Molecular Genetics, University of Virginia School of Medicine, Charlottesville, VA USA

**Keywords:** Proteases, Cryoelectron microscopy, Proteasome, Chaperones

## Abstract

The proteasome core particle (CP) can be capped by Blm10/PA200, which promotes ATP-independent degradation of disordered proteins and contributes to attenuating antigenic diversity and impacts immunotherapy upon upregulation. The assembly process that leads to Blm10-CP proteasomes remains unresolved and Blm10 is present not only at mature proteasomes but also bound to assembly intermediates. We identified and solved structures of five CP intermediate complexes by cryo-electron microscopy, two of which include Blm10. We find that CP intermediates interactions with Blm10 and Pba1/Pba2 chaperone complex are mutually exclusive and both leads to mature CP production. We further find no synergy between Pba1/Pba2 and Blm10 for CP assembly. These data suggest a parallel assembly pathway exists for Blm10 proteasomes that does not require sequential activity with Pba1/Pba2. Whereas CP affinity for Pba1/Pba2 is reduced during maturation to promote its release, no such affinity switch occurs for Blm10, further suggesting a parallel Blm10-assembly pathway for production of mature Blm10-bound CPs. Altogether, our findings reveal a functionally distinct CP assembly pathway for the production of mature Blm10-CP proteasomes.

## Abstract (web summary)

Proteasomes can be capped by regulatory particles other than the 19S complex, but how these form is unclear. Here, authors reveal cryo-EM structures of Blm10-proteasome assembly intermediates and provide evidence for parallel assembly pathways.

## Introduction

The proteasome is an essential cellular complex responsible for selectively degrading proteins into short peptides. The 26S and 30S proteasome complexes, which degrade ubiquitinated proteins, consist of a 750 kDa core particle (CP) capped by a 700 kDa regulatory particle (RP) at either (26S) or both (30S) ends. The RP contains receptors to recognize ubiquitinated proteins, deubiquitinating enzymes to remove ubiquitin, and an AAA-ATPase ring that facilitates unfolding and translocation of substrates into the CP^1–3^. The proteolytic activity occurs within the CP, which adopts a hollow cylindrical shape, with two β-rings sandwiched between two α-rings. Each ring is formed from seven homologous subunits (α1-α7 or β1-β7), with β1, β2, and β5 containing caspase-like, trypsin-like, and chymotrypsin-like proteolytic activities, respectively^4–6^. The CP cylinder has a gated entrance at either end that is closed or opened, as controlled by the N-terminal tails of the α subunits^7^. While degradative functions for CP by itself have been reported, it generally requires regulators to induce gate opening and other substrate-processing activities. In addition to the RP, other regulatory factors can cap the CP. A common factor amongst many of these regulators is a C-terminal HbYX motif (i.e., an antepenultimate hydrophobic residue, a penultimate tyrosine, and any C-terminal residue) that docks into inter-subunit pockets of the CP α-ring and contributes to gate opening.

Accurate and efficient proteasome assembly is critical for maintaining proteasome levels, the reduction of which causes proteotoxic stress and contributes to the development of neurodegenerative disorders and cancer^8–13^. The assembly of the CP is assisted by three proteasome-specific assembly chaperone complexes: Pba1/Pba2 (PAC1/PAC2), Pba3/Pba4 (PAC3/PAC4), and Ump1 (POMP). Pba1/Pba2 and Pba3/Pba4 promote α subunit ring formation; the HbYX motifs of Pba1 and Pba2 dock into the CP α5/α6 and α6/α7 pockets, respectively^14^, while at the opposite side, Pba3/Pba4 promotes α3 incorporation into the ring^15^. The β-subunits associate sequentially with the α-ring, and incorporation of β4, which follows β2 and β3, causes the steric expulsion of Pba3/Pba4^16^. Pba1/Pba2 remains bound to the immature complex until maturation, as enabled by their positioning at the outer surface of the α-ring^14,17^. Ump1 interacts with both α and β subunits at the same side of the α-ring as Pba3/Pba4 and contributes to the recruitment of the additional β subunits^14,18,19^. β7 is the last subunit to get incorporated (creating the half CP α_1-7_ β_1-7_), and its C-terminus contributes to the dimerization of two half CPs into a pre-holocomplex^20^. The final step of CP maturation, the cleavage of the propeptides from β1, β2, β5-7, and the degradation of Ump1, reduces the affinity of Pba1/Pba2, freeing the now mature CP to bind RP.

Blm10 (PA200 or PSME4)-bound mature CP functions in the ATP-independent degradation of short peptides and proteins with intrinsically disordered regions, contributing to spermatogenesis, gene regulation by degrading acetylated histones, and mitochondrial health^21–25^. More recently in humans, this regulator was discovered to be upregulated in tumors, causing attenuated antigenic diversity and impacting immunotherapy^26^. Blm10 levels are typically substoichiometric compared to CP complexes, with overexpression yielding increased Blm10-bound CP complexes at the expense of almost all 26S and 30S complexes otherwise present^27^. Blm10 also associates with CP assembly intermediates^20,28,29^. Deletion of Blm10 in yeast strains with proteasome assembly defects exacerbates growth phenotypes^20,30^, suggesting that Blm10 contributes to proteasome assembly. While many reviews of proteasome assembly do not specify a functional role for Blm10^31–35^, those that do propose a sequential model whereby Blm10 interacts with CP after Pba1/Pba2 dissociates and prior to RP binding^36,37^.

However, there is limited experimental support for this model, which remains disputed. Thus, the evolutionary benefit of Blm10 binding to assembly intermediates remains unclear, including the relevance of these interactions to regulating the levels of Blm10-bound CP versus RP-bound CP. While structures have been reported for both Blm10:CP and PA200:CP^38–40^, there is no structure of Blm10 bound to an assembly intermediate, limiting our ability to understand the changes that occur during assembly or the role played by Blm10-assembly intermediates in proteasome formation.

Here, we solved cryo-electron microscopy (cryo-EM) structures of five CP assembly intermediates. These structures indicate that Blm10 and Pba1 binding to CP intermediates is mutually exclusive. As we can detect both Blm10 and Pba1/Pba2 on early-stage and late-stage intermediates, our findings support a model whereby Blm10 and Pba1/Pba2 function in parallel CP assembly pathways, rather than sequentially acting in one common assembly pathway. Finally, the lack of interactions between Blm10 and Ump1 or β propeptides suggests that, unlike Pba1/Pba2^14^, Blm10 remains bound throughout CP maturation.

## Results

### Blm10:13S and Blm10:α-ring structures

**Fig. 1:**
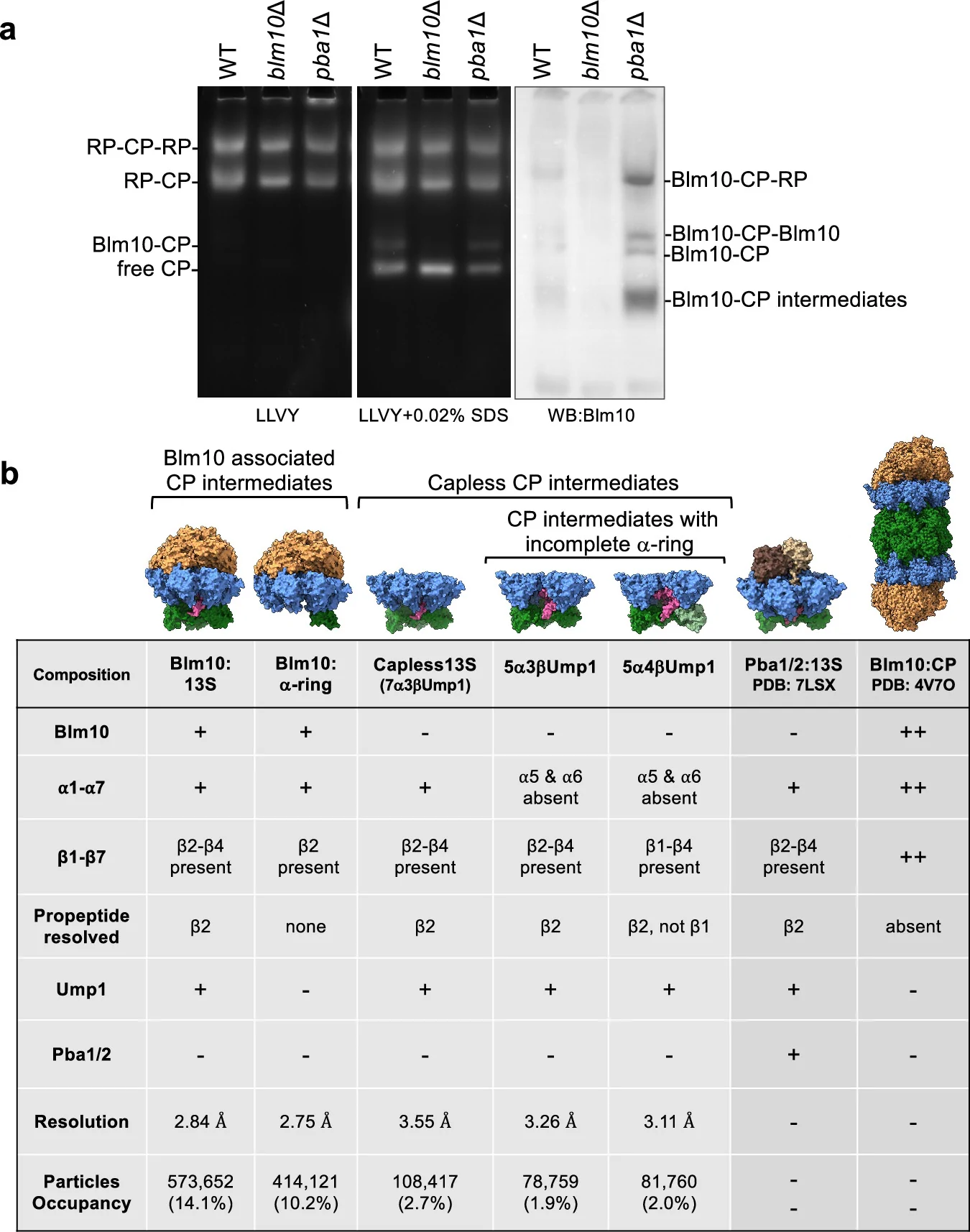
Comparison of CP assembly complex composition in *pba1*Δ cells. **a** Blm10:13S complexes are present in wild-type cells and enriched upon deletion of PBA1. Indicated yeast strains were grown for 24 h in YPD media and lysed. Lysates were separated by native PAGE and visualized using in-gel LLVY-amc peptidase activity assay in the absence or presence of 0.02% SDS. Next, the gel was transferred to PVDF membrane and immunoblotted for Blm10 (right most panel). The experiment was independently repeated three times with similar results (**b**). Cryo-EM structures of five distinct CP assembly intermediates (Blm10 associated and capless) determined in this study are compared with previously reported structures of Pba1/2:13S (also referred to as 13S; PDB: 7LSX) and Blm10:CP (PDB: 4V7O). The corresponding subunit composition, resolution, and particle occupancy are indicated. Symbols “+” and “++” denote the presence of one or two copies of the respective subunits, respectively. Blm10, **α**-subunits, β-subunits, Ump1, Pba1, and Pba2 are colored in orange, blue, green, pink, brown, and beige, respectively.

To evaluate how Blm10 contributes to proteasome assembly, we purified CP assembly intermediates by using a yeast strain with the CP chaperone Ump1 C-terminally TAP-tagged^17^. We also deleted the Pba1-encoding gene *PBA1* to omit the abundant assembly intermediate complexes 13S (onwards referred to as Pba1/2:13S), which comprises α1-α7, β2-β4, and Pba1/Pba2, and pre-15S, which contains α1-α7, β2-β6, and Pba1/Pba2^14^. This mutant strain shows the same Blm10-containing complexes observed in wild-type cells (Fig. 1a). Moreover, deletion of *PBA1* resulted in increased association of Blm10 with immature CP intermediates as well as mature CP, indicating that the deletion did not result in the accumulation of dead-end assembly intermediates (Fig. 1a). Furthermore, reconstitution experiments showed that Blm10 associated with these assembly intermediates can form Blm10-CP-RP complexes (Supplementary Fig. 1a). The purified assembly intermediates were prepared for cryo-EM data collection on a Krios G3i transmission electron microscope equipped with a Gatan3 camera, as described in Methods. Following 2D classification and refinement, we determined the structures of two Blm10-containing complexes, representing 24% of particles, to resolutions <3 Å, as well as three additional Blm10-lacking assembly intermediate complexes (Fig. 1b and Supplementary Fig. 1b). The composition of these complexes was consistent with mass spectrometry analyses from similar purifications^17^. Notably, the β2 propeptide, a feature only present in immature CP intermediates, was observed in four of these pre-assembly intermediates (Fig.1b), as discussed below.

**Fig. 2:**
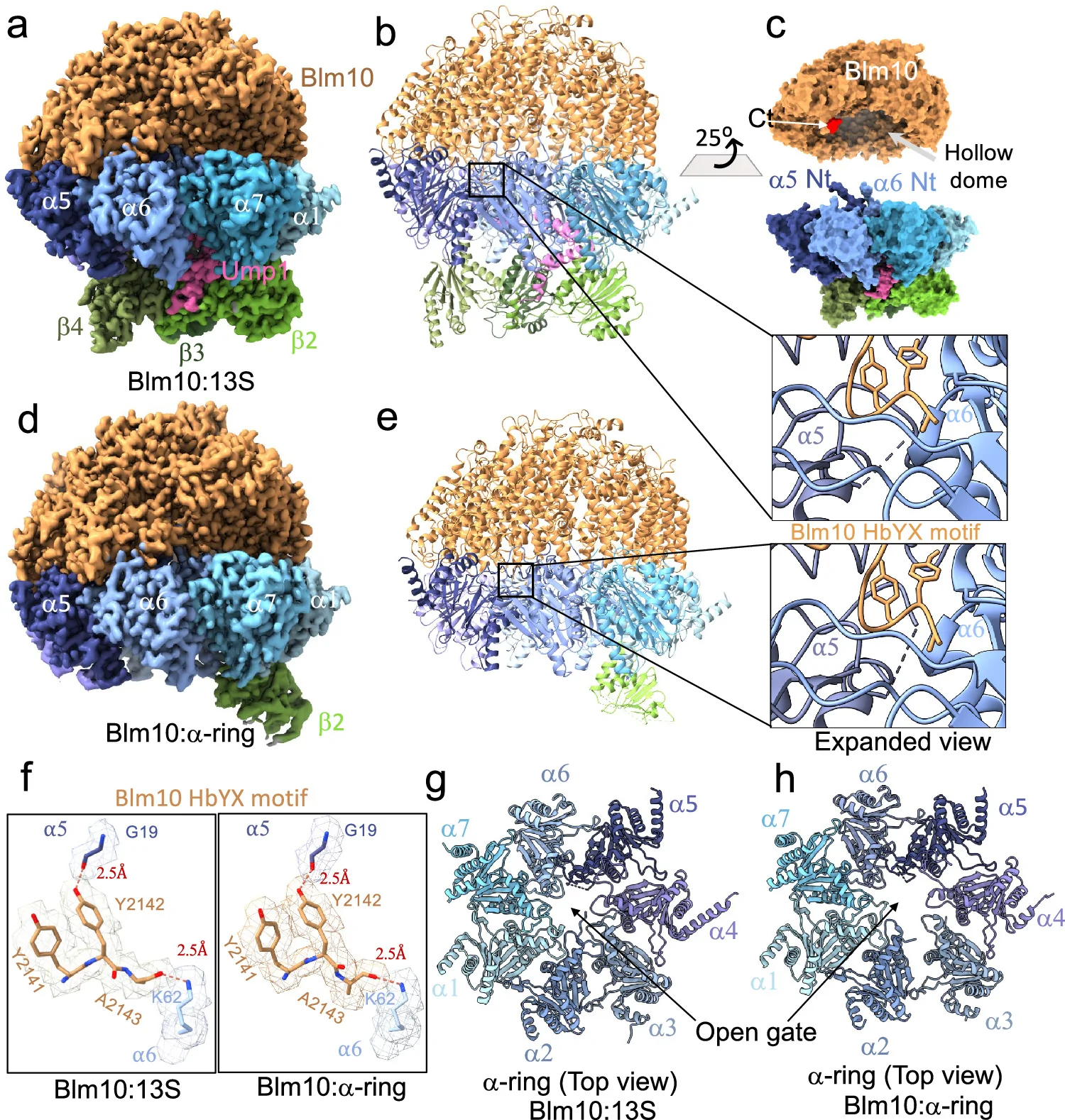
Structure of Blm10:13S and Blm10:α-ring. **a**, **b** Cryo-EM density map (2.84 Å, **a**) and corresponding ribbon diagram (**b**) of Blm10:13S which includes the complete α-ring (α1–α7, shades of blue), β-subunits β2, β3, and β4 (shades of green), Blm10 (orange), and Ump1 (pink). An expanded view highlights the insertion of the Blm10 C-terminus (HbYX motif, orange) into the **α**5/**α**6 inter-subunit pocket. **c** Surface representation of Blm10:13S with Blm10 separated and rotated by 25° to reveal termini critical for the interaction**. “**Ct” and “Nt” denote the C-terminus of Blm10 and the N-terminus of the **α**5 and **α**6 subunits, respectively. The C-terminal HbYX motif of Blm10 is colored orange. **d**, **e** same as (**a**, **b**) for Blm10:**α**-ring (2.75 Å). **f** Expanded views of the two important electrostatic interactions (red dashed lines) between the C-terminal HbYX motif of Blm10 (orange) and residues G19 of **α**5 and K62 of **α**6 (dark and light blue, respectively) in Blm10:13S (left) and Blm10:**α**-ring (right). Corresponding electron densities are shown in mesh. **g**, **h** Top views of the **α**-ring with Blm10 hidden to illustrate the open-gate CP conformation. In **b**, **e**, **g** and **h**, dashed lines connect residue endpoints where density is not observed.

Blm10:13S, comprising α1-α7, β2/β3/β4, and Ump1, was the most abundant complex and was resolved to 2.84 Å resolution (Fig. 2a, b and Supplementary Fig. 2a–e). The β2 propeptide is observed in this complex interacting with Ump1 (Supplementary Fig. 2f, g). Structurally, Blm10:13S resembles an octopus, with three stubby arms formed by the CP β subunits. Blm10 adopts a hollow dome architecture, with its C-terminal HbYX motif inserted into the α5/α6 inter-subunit pocket, while the α5 and α6 N-terminal ends extend into the Blm10 dome (Fig. 2b expanded view and Fig. 2c).

A complex containing Blm10, α1–α7, and β2, but lacking Ump1, hereafter referred to as Blm10:α-ring, was resolved to 2.75 Å resolution (Fig. 2d, e and Supplementary Fig. 2h–j). We rationalized that the tagged Ump1 used for purification was lost during sample handling; Ump1 becomes increasingly structured by interacting with the β subunits as they are added during CP assembly^18^ and a complex with fewer β subunits is likely more prone to Ump1 loss. β2 has been previously proposed to be the first β subunit added to the α-ring^14,18^, consistent with it being the only β subunit present in our Blm10:α-ring structure (Fig. 2d). The β2 propeptide is not observed, most likely because it becomes disordered following the loss of its extensive interactions with Ump1 (Supplementary Fig. 2g).

When Blm10 is bound to mature CP, there is one hydrogen bond interaction and one salt-bridge between its C-terminal HbYX motif and the α5/α6 pocket that are critical for association between Blm10 and the α-ring; disruption of these contacts abrogates Blm10 binding to CP^21,41^. Specifically, a hydrogen bond forms between the sidechain of Blm10 Y2142 and α5 G19, and a salt bridge forms between the C-terminal carboxylate of Blm10 (A2143) and α6 K62. These interactions aid in the opening of the CP gate by repositioning P17 of α5^29^. Notably, the same interactions are observed in the Blm10:13S (Fig. 2f, left panel) and Blm10:α-ring (Fig. 2f, right panel) structures. Moreover, these Blm10-bound assembly intermediates adopt an open gate CP conformation (Fig. 2g, h). Thus, the binding of Blm10 to assembly intermediates mimics structural features of the mature CP:Blm10 complex.

### Capless13S and partial α-ring complexes

**Fig. 3:**
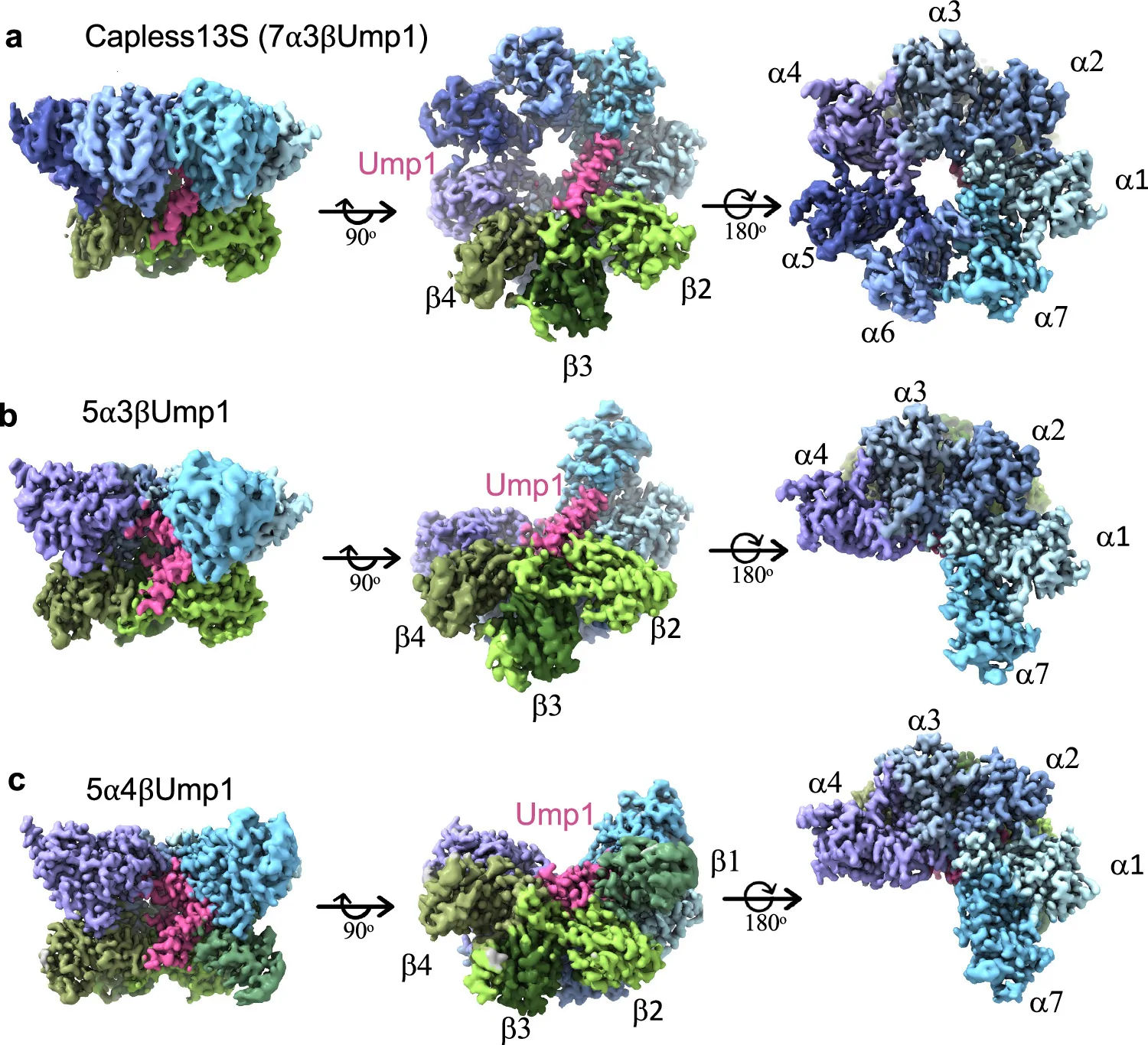
Structure of the capless13S and partial α-ring complexes. **a**–**c** Cryo-EM density maps of capless13S (7**α**3βUmp1, resolution 3.55 Å, a), 5**α**3βUmp1 (resolution 3.26 Å, b) and 5**α**4βUmp1 (resolution 3.11 Å, c) as shown in side views (left), bottom views (middle), and top views (right). **α** subunits are colored in shades of blue and purple, β subunits in shades of green, and Ump1 is pink.

Among the three complexes lacking Blm10, one complex, which we refer to as capless13S, contains a complete α-ring, Ump1, and β2/β3/β4 (Fig. 3a and Supplementary Fig. 3a–c). Considering proteasomes can assemble without Blm10 and Pba1/Pba2, this complex likely reflects a genuine 13S-like assembly intermediate, albeit it is possible that RP was lost during sample handling. Surprisingly, in the capless13S complex, the N-termini of the α subunits are disordered, and the ring has an opened gate without the docking of an HbYX motif (Fig. 3a, right panel). Potentially, this complex cycles between an open and closed state, as has been observed for CP^42^ despite X-ray crystallography only capturing a closed conformation of CP. Nonetheless, no closed form of capless13S was detected in our data, and it is possible that a closed state cannot be achieved with only β2/β3/β4 present.

The remaining complexes, 5α3βUmp1 and 5α4βUmp1, consist of α1/α2/α3/α4/α7 and Ump1, with either β2/β3/β4 (Fig. 3b and Supplementary Fig. 3d–f) or β1/β2/β3/β4 (Fig. 3c and Supplementary Fig. 3g–i), respectively. The identification of intermediates that have β subunits while lacking a complete α-ring was unexpected as the α-ring is generally thought to form first^18,33^. However, it is consistent with our previous observations^17^ and such intermediates have been observed in mutant strains^43^. Since ring structures are generally stable^44^ and the missing subunits are those with which Pba1/Pba2 or Blm10 interact (α5 and α6), the presence of these complexes suggests that Pba1/Pba2 and Blm10 facilitate the incorporation of α5 and α6 into the α-ring. There is no structural indication that 5α3βUmp1 and 5α4βUmp1 represent dead-end products because all subunits are correctly ordered in these intermediates. Thus, at least in this *pba1*Δ *background*, we find evidence that assembly does not always proceed by formation of the α-ring first.

### Blm10:13S and Ump1-mediated conformational changes

**Fig. 4:**
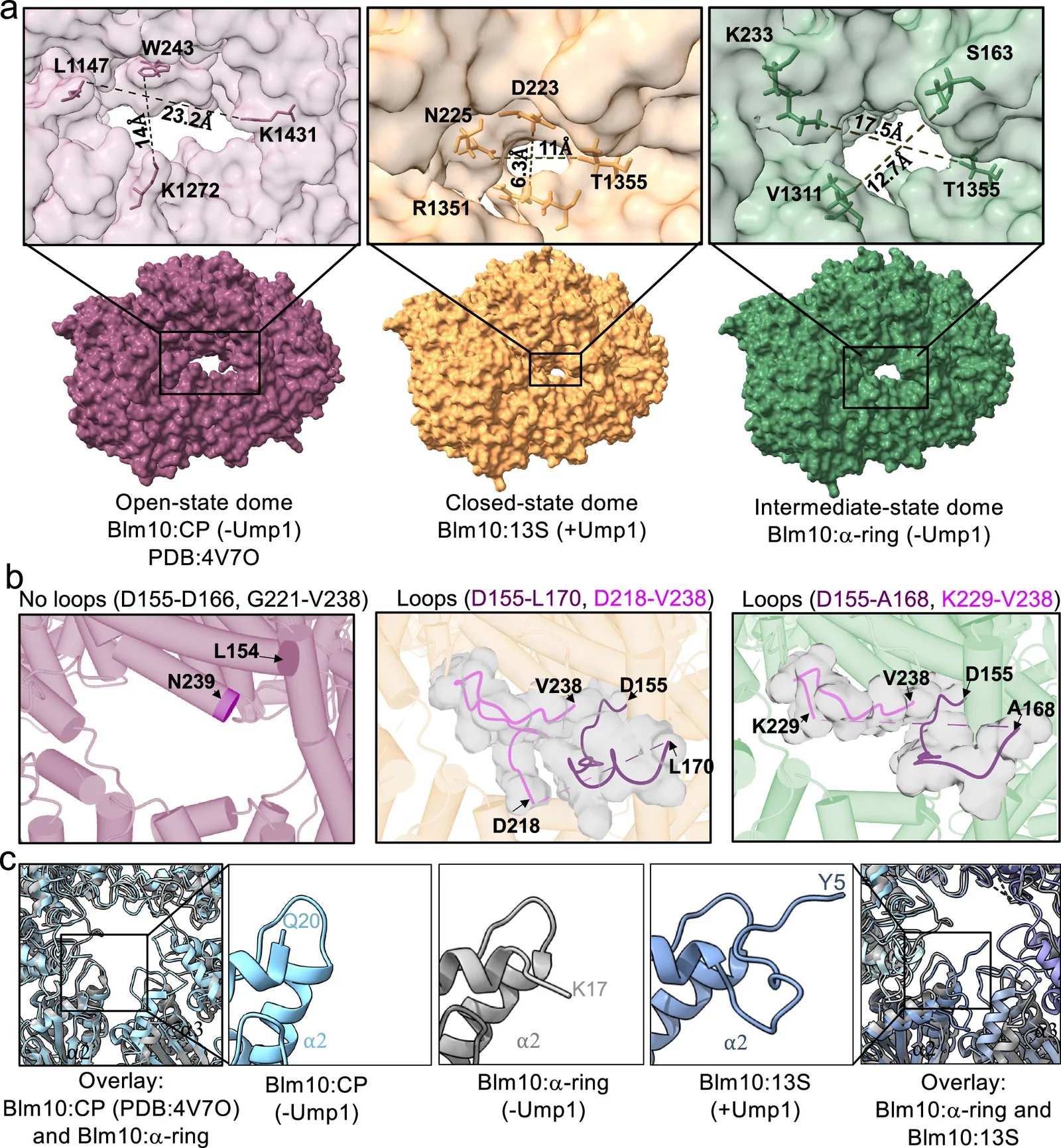
Ump1 mediated changes in the Blm10:CP interaction. **a** Comparison of the maximum diameter of the Blm10 dome opening in Blm10:CP (magenta; PDB: 4V7O), Blm10:13S (orange), and Blm10:**α**-ring (green). Expanded views with corresponding measurements are shown on top. **b** Expanded views of Blm10 dome opening, with **α** helices represented as tubes. In Blm10:CP (left), no loop residues are resolved between D155 and V238. In Blm10:13S (middle), the dome opening is covered by loops D155-L170 (dark pink) and D218-V238 (light pink). In Blm10:**α**-ring (right), the dome is partially occluded by loops D155-A168 (dark pink) and K229-V238 (light pink). Electron densities corresponding to these loops are shown in gray. **c** Superposition of the **α**-ring from Blm10:CP (cyan) and Blm10:**α**-ring (gray) is shown in the leftmost panel. Expanded views highlight the first resolved N-terminal residues of **α**2 in Blm10:CP, Blm10:**α**-ring, and Blm10:13S, as indicated. The far-right panel shows the superposition of the **α**-ring from Blm10:13S (shades of blue) and Blm10:**α**-ring (gray).

During CP assembly, Ump1 directly interacts with multiple α and β subunits and appears to contribute to propeptide cleavage^19^. Furthermore, Ump1 is degraded as part of CP maturation. Thus, the presence or absence of Ump1 presents as a logical driver of structural changes that occur upon CP maturation. Comparison of Ump1-containing Blm10:13S to Ump1-lacking Blm10:α-ring or Blm10:CP revealed several differences including a conformational change in the Blm10 dome. The Blm10 dome in Blm10:CP contains a large lateral opening measuring approximately 14 × 23.2 Å that is situated laterally above α2 and α3 (Fig. 4a, left panel). This opening is speculated to allow entry and subsequent degradation of intrinsically disordered proteins^29^. The corresponding opening in Blm10:13S is substantially narrower, at 6.3 × 11 Å (Fig. 4a, middle panel). This constriction is caused by Blm10 loops spanning D155-L170 and D218-V238, which are resolved in the Blm10:13S structure, but missing in the Blm10:CP structure (Fig. 4b middle and left panel and Supplementary Fig. 4a)^29^. In Blm10:13S, multiple hydrogen bonds and hydrophobic interactions stabilize these loops (Supplementary Fig. 4b). The reduced opening in Blm10:13S (6.3 Å by 11 Å) is expected to sterically hinder entry of an unfolded polypeptide, as it approximates the dimensions of the closed CP gate (9 Å diameter versus open gate ~20 Å) or the closed Sec61 complex (9-15 Å versus 40-60 Å when open)^45,46^. Therefore, we refer to this configuration as in a closed state. The changes in the dome are accompanied by changes at the interface between α3 and Blm10 loops that connect helix 49 (H49) to helix 50 (H50) and helix 46 (H46) to helix 47 (H47). In Blm10:13S, hydrophobic contacts and three hydrogen bond interactions are observed at this interface that are absent in Blm10:CP (Supplementary Fig. 4c, d). In the Blm10:α-ring structure, which lacks Ump1, the dome opening is intermediate in size, at 12.7 × 17.5 Å (Fig. 4a, b, right panel). The Blm10 loops D155-A168 and K229-V238 are observable, but D218-G228, which was observed in the Blm10:13S structure, is not (Fig. 4b, right panel). In all, these data suggests that the Blm10 dome can transition between closed and open conformations. Such putative allosteric regulation might be functionally relevant, as Blm10 has been proposed to mediate degradation of specific protein substrates^21–23,41^.

Another structural difference correlated with the presence of Ump1 is the status of the α2 N-terminal residues. These residues are disordered in Blm10:CP, as indicated by the absence of electron density for the first 19 residues (Fig. 4c, panel 1 and 2). In contrast, in Blm10:13S, residues from Y5 onwards are resolved in the structure and partially occlude the CP gate (Fig. 4c, panel 4 and 5, and Supplementary Fig. 5). The α2 N-terminus is disordered in Blm10:α-ring, more closely resembling Blm10:CP (where Ump1 is absent) than Blm10:13S (Fig. 4c, panel 3). This comparison suggests that the maturation of CP, which is accompanied by degradation of Ump1 leads to increased flexibility of the α2 N-terminal residues (accompanied by a more opened CP gate) and a transition of the Blm10 dome from a closed to an opened conformation, which likely contributes to complex activation. CP assembly and the degradation of Ump1 are likely to induce additional conformational rearrangements. For example, superimposing the β-subunits from Blm10:13S and Blm10:CP reveals steric clashes that must be resolved during maturation. This requires a rearrangement of β2 and β3 to avoid steric clashes with Ump1 and the β2 pro-peptide (Supplementary Fig. 6).

### Differences in specificity for 13S between Blm10 and Pba1/2

**Fig. 5:**
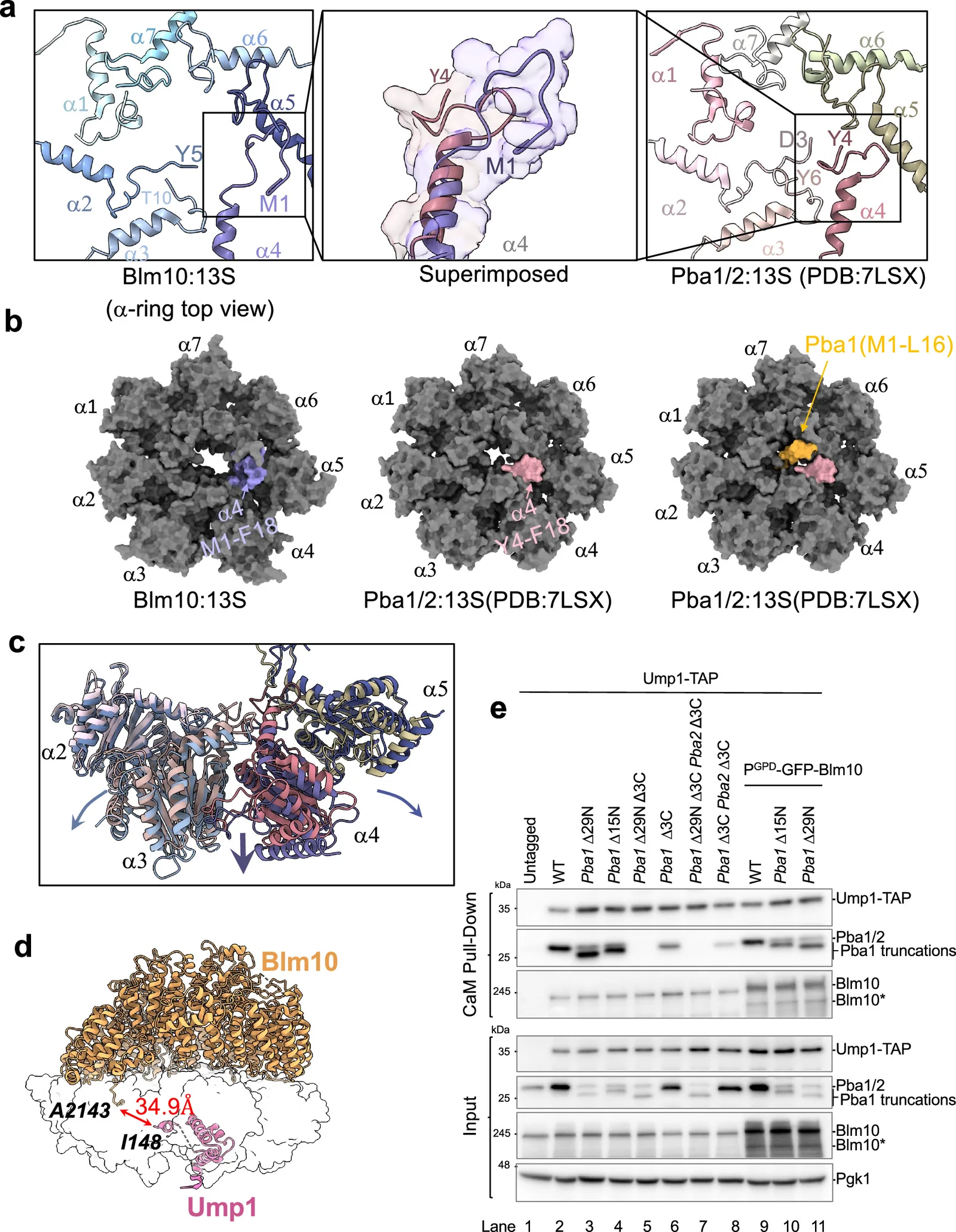
Structural differences between Blm10:13S and Pba1/2:13S. **a** N-terminal tails of α1-α7 indicating the first resolved residues in Blm10:13S (left) and Pba1/2:13S (right). The middle panel shows an expanded view of the superimposed **α**4subunit’s N-terminal residues, highlighting their distinct orientations in Blm10:13S and Pba1/2:13S. **b** Top view of the **α**-ring illustrating the extent of gate opening upon binding of Blm10 (left) or Pba1/Pba2 (middle), as well as the displacement of α4subunit in each complex. The right panel is identical to the middle panel but includes the Pba1 N-terminal tail (orange). **c** Superposition of the **α**-ring from Blm10:13S (shades of blue and purple) and Pba1/2:13S (shades of pink and green), showing only the **α**2–**α**5 subunits. Arrows indicate the downward displacement of the subunits, with the largest shift observed for **α**4. **d** Blm10:13S structure with Blm10 and Ump1 displayed as ribbons and all other subunits rendered as transparent white surfaces. The distance between the C-terminal residue of Blm10 (A2143) and Ump1 (I148) is indicated by a red solid line. **e** The Pba1 N-terminus contributes to its affinity for immature CP. Strains with N- or C-terminal truncations of Pba1 were lysed, and Ump1-containing complexes were purified using calmodulin (CaM) resin. Following the pull-down, samples were separated by SDS-PAGE, transferred to a PVDF membrane, and immunoblotted for the indicated proteins. Top panel shows immunoblot analysis of Ump1-TAP CaM pull-down from the specified yeast strains. Bottom panel shows the total cell lysate (input) from the respective strains. Blm10* denotes a Blm10 specific band, likely reflecting a breakdown product. Uncropped immunoblots are provided in source data file. Similar results were obtained in three independent experiments.

The HbYX motifs of Blm10 and Pba1 occupy the same α5/α6 inter-subunit pocket in CP (Fig. 2b, expanded view)^14,47^. Nevertheless, alignment of Blm10:13S with Pba1/2:13S reveals notable differences in the CP gate opening (Fig. 5a, b). In Blm10:13S, the α2-α5 side of the α-ring is displaced downward toward the β-ring, with the largest shift observed for α4 (Fig. 5a, c). In Blm10:13S, electron density is absent for α2 M1 to R4, α3 M1 to R9, and the α4 N-terminus is shifted outward, collectively resulting in an open CP gate (Fig. 5a, left and middle panel). However, despite this open gate conformation, the entry of polypeptides from the top is unlikely, as the Blm10 dome is closed (Fig. 4a, middle panel). In Pba1/2:13S, although the HbYX motif is similarly docked, most of the N-terminal residues of α2, α3 and α4 are ordered due to interactions with the Pba1 N-terminal tail, which plugs the gate (Fig. 5a, b middle and right panels)^14^.

The N-terminal tail of Pba1 also interacts with Ump1 and the β5 propeptide^14^. In contrast, Blm10 does not directly contact Ump1; the closest residue, A2143, lies 34.9 Å away (Fig. 5d). Despite this difference, Ump1 is positioned almost identically in both 13S structures (Supplementary Fig. 7a), indicating neither Pba1 nor Blm10 significantly influence its positioning. The lack of direct interactions between Blm10 and intermediate complex specific components, such as Ump1 or β subunit propeptides, suggests that Blm10 affinity is not dramatically altered during CP maturation as Ump1 or the propeptides are degraded or processed. Consistent with this interpretation, Blm10 remains associated in the absence of Ump1, as observed in the Blm10:α-ring structure (Fig. 1) and Blm10-bound CP complexes. In all, our data provide a structural rationale for the presence of Blm10 on both assembly intermediates and mature CP, while Pba1/Pba2 solely associates with intermediates^17,27,47^.

To determine whether the Pba1 N-terminal tail plays a role in regulating the relative association of Blm10 versus Pba1/Pba2 with the 13S intermediate, we aimed to disrupt the Ump1-Pba1 interaction by mutating Ump1 residues R93 and E89, which interact with the Pba1 N-terminus (Supplementary Fig. 7b). However, we did not see any difference in Pba1 binding to Ump1, possibly because the propeptides also interact with Pba1. Therefore, we next examined the effects of N-terminal truncations of Pba1. Using small deviations compared to previous efforts^48^, we were able to readily detect Pba2 and N-terminally truncated Pba1 variants (either 15 residues, *pba1*Δ15 N, or 29 residues, *pba1*Δ29 N) in the cell lysates, albeit at lower levels compared to full length Pba1 (Fig. 5e, bottom, lane 3 and 4 compared to lane 2). While the N-terminal truncations by themselves did not abrogate Pba1/Pba2 binding to assembly intermediates, we detected an impact on binding when combined with truncations of the HbYX motif of Pba1 alone (*pba1*Δ3 C) or in combination with Pba2 (*pba1*Δ3 C *pba2*Δ3 C) (Fig. 5e). Strikingly, the deletion of both Pba1 and Pba2 HbYX motifs showed more residual binding to 13S than Pba1 with its N- and C-terminus truncated (*pba1* Δ29 N Δ3 C, Fig. 5e, lane 8 versus lane 5). In all, these data show the importance of the Pba1 N-terminus for binding to intermediates.

Nevertheless, altering Pba1/Pba2 interaction with 13S did not alter the amount of copurified Blm10 (Fig. 5e), raising the possibility that the substoichiometric levels of Blm10 relative to CP in yeast could obscure any putative competition. Indeed, upon overexpression of Blm10, the *pba1*Δ15 N and *pba1*Δ29 N showed a larger reduction in Pba1/Pba2 association with Ump1-containing assembly intermediates as compared to full length Pba1 (Fig. 5e, lane 2, 3, and 4 versus 9, 10, and 11). Thus, the N-terminal tail of Pba1 contributes to the high affinity of Pba1/Pba2 for assembly intermediates and helps to prevent any excess Blm10 from displacing Pba1/Pba2 from assembly intermediates.

Taken together, our analyses highlight a crucial difference between Blm10 and Pba1/Pba2 regarding their interaction within the 13S assembly intermediates. For Pba1/Pba2, there are interactions with Ump1 and β-subunit propeptides that contribute to an affinity switch whereby Pba1/Pba2 loses affinity upon maturation of the CP (i.e., when Ump1 and propeptides get degraded). Blm10 lacks these interactions and does not undergo an affinity switch to release it upon CP maturation. Instead, Blm10 remains associated with CP upon maturation to form Blm10:CP complexes.

#### Parallel pathways of CP assembly

**Fig. 6:**
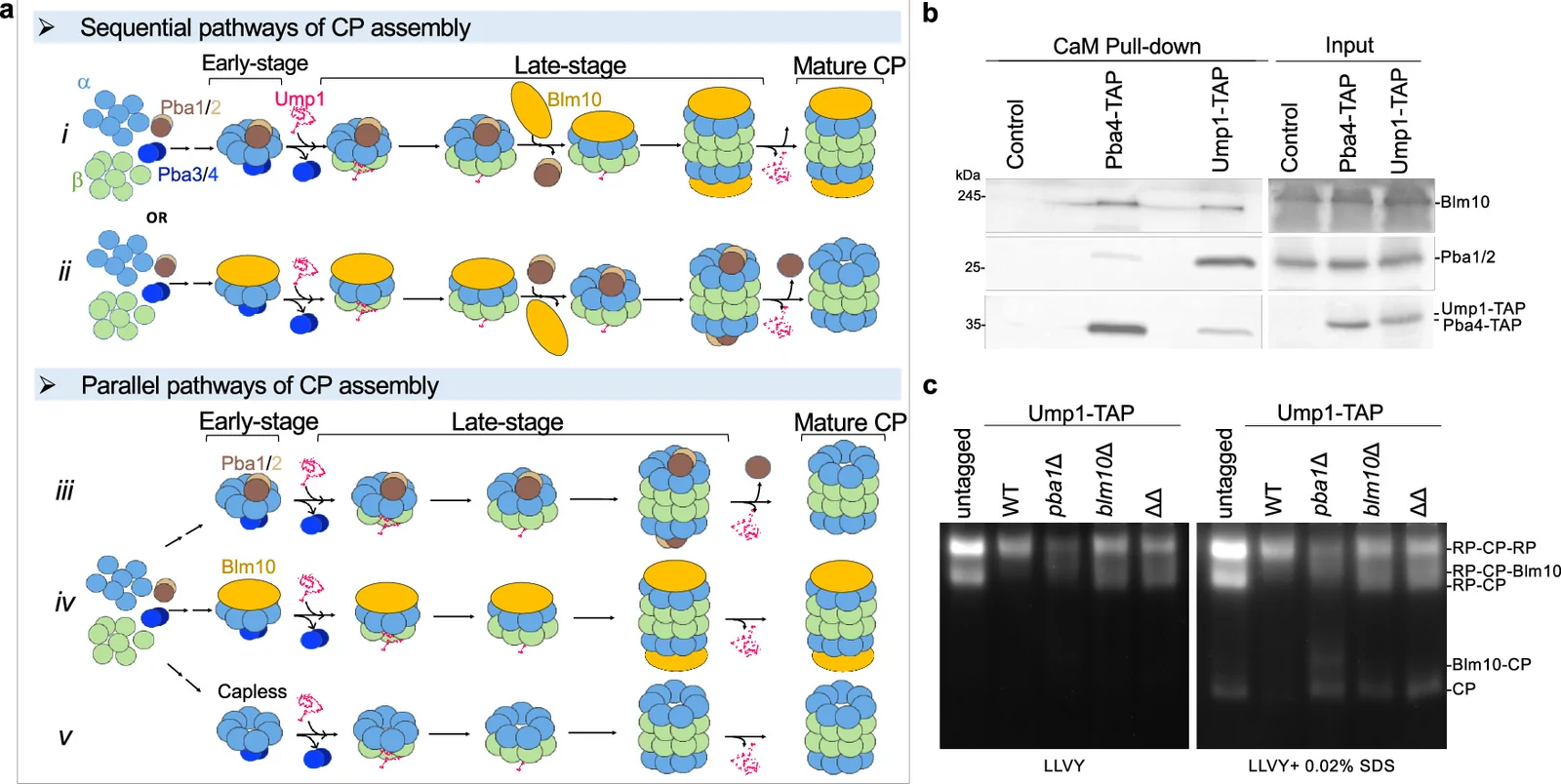
Parallel pathways of proteasome assembly. **a** Conceptual model of CP assembly via sequential or parallel assembly pathways. The 20S assembly starts with the formation of **α**-ring assisted by Pba1/Pba2 and Pba3/Pba4 heterodimers. The β subunits are sequentially incorporated with the assistance of Ump1, which replaces the Pba3/Pba4 dimer. Together with β subunit propeptides, Ump1 promotes assembly of the half CP rings into a pre-holocomplex. In a sequential pathway (top), either Pba1/Pba2 binds in the early stages of assembly and gets replaced by Blm10 in the late stages (*path i*) or vice versa (*path ii*), ultimately resulting in mature free CP (details in text). In a parallel pathway (bottom), CP assembly follows three parallel pathways, (*path iii*) Pba1/Pba2-associated intermediates, (*path iv*) Blm10-associated intermediates, or (*path v*) with capless intermediates (RP associated, potentially). **b** CaM based pull down assay to detect the presence of Pba1/Pba2 and Blm10 at early stage (Pba4 pull-down) and late stage (Ump1 pull-down) assembly intermediates (left panel). Blm10 and Pba1/Pba2 are present at early and late-stage CP assembly intermediates. The right panel shows the total cell lysates (input) from the respective yeast strains. The wild-type strain lacking a TAP tag served as a negative control. **c** Native gel showing proteasome activity in total cell lysates of different strains. The left panel shows activity without SDS and right panel shows activity after incubating the gel in 0.02% SDS. For experiments in **b**, **c** uncropped immunoblots are provided in source data file and these experiments were performed at least three times producing similar results.

The binding of Blm10 and Pba1/Pba2 to an α-ring is mutually exclusive, and previous models have proposed that they bind sequentially^36,37^. Sequential binding can occur by Blm10 association following displacement of Pba1/Pba2 (Fig. 6a, top panel, path *i*), or alternatively, with Blm10 binding first (Fig. 6a, top panel, path *ii*). The latter model is inconsistent with the retention of Blm10 on mature CP. Yet, Pba1 is found on late assembly complexes, such as Ump1-containing 13S, 15S, and pre-holoenzyme complexes, which is inconsistent with the former model. Therefore, we postulated that instead of a sequential pathway, a parallel assembly pathway is more consistent with the experimental data and protein structures (Fig. 6a, lower panel). If Blm10 and Pba1/Pba2 act in parallel (i.e., different) pathways of assembly, Blm10 and Pba1/Pba2 would both be present at both early and late assembly intermediates. In a sequential pathway, at least one of them should be absent early or late in assembly.

Early steps in the CP assembly pathway can be distinguished from later steps by the different chaperones that associate with the complex; Pba3/Pba4 binds the α-ring during early assembly steps (Fig. 6a, dark blue), whereas Ump1 binds to the complex at later steps (Fig. 6a, red). A steric clash between Pba4 and Ump1 ensures that upon incorporation of Ump1, Pba3/Pba4 is expelled from the assembly intermediate, providing a clear definition of early versus late assembly intermediates; while a recent cryo-EM study revealed one structure with PAC3/PAC4 and POMP within the same complex amongst a series of human CP assembly intermediates^18^, it should be noted that these structures were purified from heterologously expressed human proteins. All studies looking at endogenous complexes have not detected this complex, suggesting that it is a short-lived, low abundance complex under physiological conditions. In both yeast and humans, Pba3/Pba4 leaves early during CP assembly, Ump1 arrives later and remains bound until a mature CP has been produced^18,33^.

Performing pulldowns from Pba4-tagged (early) and Ump1-tagged (late) yeast strains, we detected Pba1/Pba2 in both purifications (Fig. 6b). Blm10, which we know binds to mature CP as well as the 13S complex that contains Ump1 (our Blm10:13S structure)^49^, also copurified with early- and late-stage intermediates (Fig. 6b, Pba4-TAP and Ump1-Tap lanes, respectively). Thus, both Blm10 and Pba1 are present in early as well as late stages of CP assembly, which is consistent with parallel assembly pathways. As we also solved the structure of capless13S (i.e., 13S-like complex without Blm10 or Pba1/Pba2 (Fig. 1 and Fig. 3a), there are likely three parallel assembly pathways to form ½ CP: Blm10-dependent, Pba1/Pba2-dependent, and a pathway that is independent of both (potentially assisted by RP^20^). The latter pathway must exist, as proteasomes can still form in strains deleted of both PBA1 and BLM10 (Fig. 6c).

If the Pba1/Pba2 and Blm10 dependent assembly pathways are redundant and function to ensure robust production of mature CP, their combined deletion should be more detrimental for cells. As Rpn4-dependent upregulation of proteasome subunits compensates for the absence of Pba1/Pba2^50^, we deleted BLM10 and PBA1 in the *rpn4*Δ background. The *rpn4*Δ *pba1*Δ cells (unlike *rpn4*Δ *blm10*Δ) showed sensitivity to heat stress and the arginine analog canavanine (Supplementary Fig. 8). This sensitivity was not exacerbated in the *rpn4*Δ *pba1*Δ *blm10*Δ cells, suggesting Blm10 and Pba1/Pba2 do not have redundant roles. To explore this further, we utilized a β7 mutant with a truncation that removes the last 19 codons, β7^CTE^, which has an assembly defect and shows synergy with the deletion of Blm10^20^. In our background, we did not observe the same phenotype (Supplementary Fig. 9a). However, upon removal of the transcriptional terminator, we did observe this mutant become sensitive to stress, which was modestly exacerbated by the deletion of Blm10 (Supplementary Fig. 9a and b). Deletion of Pba1 in this background did not further sensitize these cells (Supplementary Fig. 9c). In all, our data suggest Pba1/Pba2:13S and Blm10:13S are intermediates in parallel assembly pathways that serve separate functions and show little to no redundancy. That notwithstanding, hybrid proteasome complexes (such as Blm10:CP:RP) exist, which presumably utilize ½ CPs derived from the parallel pathways.

## Discussion

The accurate and efficient assembly of proteasomes depends on proteasome-specific chaperones, including the CP chaperones Pba1, Pba2, Pba3, Pba4, and Ump1. Here, we report the cryo-EM structure of the Blm10:13S assembly intermediate, revealing mutually exclusive binding of Blm10 and Pba1/Pba2 to the 13S based on overlapping interaction surfaces on the α-ring and shared docking of their HbYX motifs to the α5/α6 inter-subunit pocket. These findings raise questions regarding the timing and mechanism of Blm10 binding to the α-ring and whether Pba1/Pba2 remains bound until the CP is fully assembled. Our biochemical data show that both Blm10 and Pba1/Pba2 are present early in assembly (Pba4-based pulldown) and late in assembly (Ump1-based pulldown), which is inconsistent with the model that Blm10 binds after Pba1/Pba2 in the assembly pathway^36,37^. Instead, we propose a model whereby Blm10:13S, Pba1/2:13S, and capless13S represent intermediates in parallel pathways of CP assembly (Fig. 6a, lower panel).

In the model of parallel assembly pathways, there are numerous advantages that would provide the cell with benefits and adaptability. First, parallel pathways can provide robustness to the CP assembly process by having another pathway available when one is compromised. Indeed, upon deletion of PBA1, the upregulation of proteasome subunits (via rpn4 stabilization) becomes critical, and Blm10 or RP might substitute for Pba1/Pba2 function^20^. However, the lack of a synergy phenotype between BLM10 and PBA1 in the *rpn4*Δ or β7^CTE^ backgrounds, suggest this is not an important function of the Blm10:13S assembly pathway.

Another function of parallel assembly pathways could be the ability to generate heterogenous proteasome complexes, with their relative abundance controlled at the step of assembly. One striking difference between the Pba1/Pba2-dependent and Blm10-dependent pathway is the different fates of the proteins following CP maturation. For Pba1/Pba2, there is a dramatic drop in affinity for the CP when propeptides are removed and Ump1 is degraded^17^. This affinity switch ensures Pba1/Pba2 is tightly bound during assembly, but not associated with mature CP. Thus, Pba1/Pba2’s sole purpose is to allow for efficient CP assembly, after which the mature CP is available for association with RP or PA28 family members (absent in yeast). In the Blm10:13S structure, Blm10 is 34 Å away from Ump1 and does not have any direct interactions with immature CP features (Ump1 or propeptides). As such, the degradation of Ump1 upon CP maturation does not impact the affinity of Blm10 for the α-ring. Consistent with this model, we determined the structure of Blm10:α-ring, demonstrating stable Blm10 association in the absence of Ump1. Thus, it appears that the function of Pba1/Pba2 dependent assembly is the formation of CP, while the Blm10 dependent assembly pathway leads to the formation of Blm10-bound CP complexes. Since Blm10 and the human ortholog PA200 are responsible for the degradation of a variety of substrates^23,24,41^, this parallel pathway might ensure the presence of a certain amount of Blm10-bound CP complexes in the cell. Interestingly, overexpression of Blm10 in yeast, or the observed high expression of PA200 in mouse testis is accompanied by high cellular levels of Blm10 (PA200)-bound CP and reduced 26S and 30S proteasomes in cells^51^. It remains to be determined to what extent these are formed from competition between RP and Blm10 for mature CP binding versus being driven by this alternative assembly pathway directly. If the off rates of RP and Blm10 in vivo are low, the assembly pathway might be particularly important.

A third putative function of parallel pathways is to deal with misassembled CP intermediates. The existence of proteasome specific assembly chaperones illustrates how complex and crucial the assembly of proteasomes is and reflect the potential of errors in assembly. Since the deletion of BLM10 does not have a substantial impact on CP assembly in general, it is intriguing that higher amounts of Blm10-bound CP are reported in mutants that result in CP assembly defects, such as *ump1*Δ^30^ and *PRE4*-CTEΔ^20^. All these mutants are likely to have reduced fidelity in assembly. Blm10 has also been reported to associate with CPs with an open gate^30^ and the α3 knockout strain, which has α4-α4 proteasomes with a wider CP gate, also show more Blm10 bound mature CP complexes^52^. Collectively, this might reflect a functional link between a non-canonical α-ring and Blm10. In humans, this property might have further evolved, whereby PA200 functions with a specialized α subunit, such as in spermatozoa where both PA200 and α4 s are highly expressed^23,53^.

The very early steps of CP assembly, where individual α subunits combine to form an α-ring remain poorly characterized. The structures we solved that lack a complete α-ring, suggest that there is not a strict requirement for the formation of an α-ring before β-subunits are added, as α1, α2, α3, α4, and α7 form a stable intermediate with β2, β3, β4, plus or minus β1. The formation of this complex would not involve the Pba1/Pba2 chaperone, as Pba1/Pba2 intimately binds to α5 and α6 and seems to help with the incorporation of these subunits into the α-ring. We purified α5β3Ump1 and α5β4Ump1 from a *pba1*Δ strain and in wild-type cells, these two intermediates are less likely to accumulate. Pba1/Pba2 together with α5 and α6 would readily be incorporated to form Pba1/2:13S, as this complex has β subunits with propeptides and Ump1, both of which bind the Pba1 N-terminal tail (Supplementary Fig. 7a, b). This leaves the possibility open that Blm10 can compete with Pba1/Pba2 for the assembly intermediate particularly when an α-ring is being formed or prior to Ump1 and β subunit incorporation. Consistent with this possibility, a recent cryo-EM structure of a PAC1/PAC2-bound α-ring showed the N-terminal tail of at least PAC1, was not involved in binding to this intermediate^54^.

In summary, our data show the existence of three parallel pathways of CP assembly and provide insight into the early assembly process by questioning the strictly α-ring first assembly path. Our data reveal an additional level of complexity to an already complex process. Because we observe little to no free Blm10 in cell lysates^27^, it appears that the Blm10-dependent assembly pathway ensures and enables cells to produce a certain level of Blm10 (or PA200)-bound proteasomes. This allows Blm10 expression to regulate the balance between RP-bound CP and Blm10-bound CP complexes. This mechanism of regulation has direct relevance in understanding tumors where PA200 is upregulated and influences immunotherapy responses^26^. It will also aid in understanding the physiological role and regulation of PA200 in spermatogenesis, mitochondrial health and the degradation of acetylated histones^21–25^.

## Methods

### Yeast strains

All strains were constructed in background *MAT A his3Δ0 leuΔ0 met15Δ0 ura3Δ0* unless mentioned otherwise (Supplementary Table 1). Standard PCR based procedures (primers and plasmid templates presented in Supplementary Table 2 and 3) were applied for the gene deletions. Crispr Cas9 was used to generate Pba1, Pba2, and Ump1 mutations. Primers and synthesized DNA fragments were ordered from Integrated DNA Technologies. Plating assays for yeast phenotype analyses were done using 4-fold dilutions in 96 wells plate and transfer of cells to plates using a pin array. Plates were incubated 2-5 days at 30 °C or indicated temperatures^55^.

### CaM-based pull-down assay

Indicated yeast strains were grown for 4 h starting at an OD_600_ = 0.38. Cell lysis was done by cryogrinding. Calmodulin (CaM) resin (G-Biosciences) was prewashed three times with EDTA-free lysis buffer (50 mM Tris-HCl, pH 7.5, 5 mM MgCl_2_, 1 mM ATP, 2 mM CaCl_2_). The washed resin was incubated with the lysate for 1 h at 4 °C followed by three washes with wash buffer (50 mM Tris-HCl, pH 7.5, 5 mM MgCl_2_, 1 mM ATP, 2 mM CaCl_2_, 50 mM NaCl). The proteins bound to CaM resin were subjected to SDS-PAGE.

### Western blot analysis

Yeast strains were grown at 30 °C and harvested when the OD_600_ reached 1. Cell pellets equivalent of OD_600_ = 2 were collected by centrifugation, then promptly lysed or stored at −80 °C. Lysis was performed using an alkaline lysis method or by grinding in Liquid N_2_^56,57^. Briefly, the pellets were resuspended in 100 μL distilled water with the addition of 100 μL of 200 mM NaOH, followed by a 5-min incubation at room temperature. Cell suspensions were pelleted, resuspended in 50 μL SDS-PAGE sample buffer (0.06 M Tris–HCl, pH 6.8, 5% glycerol, 2% SDS, 50 mM DTT, 0.0025% bromophenol blue), boiled at 98 °C for 5 min, and supernatant was collected. Proteins were separated on SDS-PAGE and Western blots were done using antibodies against Blm10 (Rabbit polyclonal; Enzo Life Sciences BML-PW0570), Pba1/2^17^, CBP (Mouse; GenScript CBP Tag Antibody cat. No. A01798) and Pgk1 (Invitrogen, catalog no.459250) all in 1:1000 dilution. Secondary antibodies conjugated to horseradish peroxidase were from Rockland Immunochemicals. HRP activity was visualized using the Immobilon Forte Western HRP substrate (Millipore). Images were acquired using a Gbox imaging system (Syngene) and captured with GeneSys software.

### Native gel analyse

Yeast cells were harvested at indicated times. Cell pellets equivalent to OD_600_ 50 were centrifuged and lysed by cryo-grinding under non-denaturing conditions as described previously^56,58^. Protein concentrations were measured using nanodrop. Equal amounts of protein (300 μg) for each strain were loaded on native gel (3.5%). The activity of native proteasome complexes was visualized by an in-gel LLVY-AMC peptidase activity assay described previously^56^. Images were acquired using Gbox imaging system (Syngene).

### Assembly intermediate purification

Proteasome assembly intermediate purification was carried out by two step TAP purification method^59^. Specific yeast strain with Ump1-TAP tag was grown to a final OD_600_ ~ 10.0 in a 9 L YPD media. 105 g cell pellet was collected and resuspended in 1.5X pellet volume of lysis buffer (50 mM Tris-HCl, pH 8.0, 1 mM EDTA, Protease inhibitor Roche cOmplete mini tablet) and lyzed by French press (1200 Psi.). IgG beads (MP Biomedical; 1 ml bed volume per 50 g cell pellet were added to the lysate and incubated for 1 h at 4 °C. IgG Beads were washed with 50 bed volumes of wash buffer 1 (50 mM Tris-HCl, pH 7.5, 100 mM NaCl, 1 mM EDTA) followed by 15 bed-volumes of cleavage buffer (50 mM Tris-HCl, pH 7.5, 5 mM MgCl_2_, 1 mM DDT). Overnight (at 4 °C) incubation of washed resin in the cleavage buffer with 10 μl TEV protease (Invitrogen) was done to elute the proteasome intermediates with CBP part of the TAP tag still intact with the CP assembly intermediates. To the eluted protein sample (total 5 mg), 2 mM CaCl_2_ was added prior to performing the second affinity purification using CaM (calmodulin SepharoseTM 4B) resin (2 ml slurry). Following 1.5-h incubation, the resin was washed twice with wash buffer 2 (50 mM Tris-HCl, pH 7.5, 5 mM MgCl_2_, 2 mM CaCl_2_, 50 mM NaCl) and once with wash buffer 3 (50 mM Tris-HCl, pH 7.5, 5 mM MgCl_2_, 50 mM NaCl with no CaCl_2_). Final elution was done using buffer with 2 mM EGTA (50 mM Tris-HCl, pH 7.5, 5 mM MgCl_2_, 2 mM EGTA, 1 mM DDT) after 30 min incubation. The eluate was concentrated to 1.8 mg/ml.

### Cryo-EM sample preparation and data acquisition

3 μL of the purified sample (1.8 or 0.9 mg/mL) were applied on Quantifoil® R 1.2/1.3 Cu 400 mesh grids (Electron Microscopy Sciences, cat. Q4100CR1.3), that had been glow-discharged three times (0.42 mbar, 15 mA for 45 s.) by using a PELCO easiGlow glow discharge system (Ted Pella). Grids were vitrified in 100% liquid ethane using a Leica EM GP 2 plunge freezer (Leica Microsystems) with a blot time of 1 s or 3 s, a blot distance of 43 mm, and 80% humidity. Cryo-EM data were collected on a 300 kV Titan Krios G3i transmission electron microscope (Thermo Fisher Scientific) equipped with a K3 direct electron detector (Gatan) and an energy filter (Gatan). The images were acquired at a nominal magnification of 81,000, corresponding to a calibrated pixel size is 1.068 Å per pixel. Each movie consisted of 58 frames recorded at a total dose of 60 elections/Å^2^. A total of 5235 movies were collected using the EPU program (v.3.3.0; Thermo Fisher Scientific), with defocus values ranging from −2.5 to −0.9 μm, at 0.2 μm intervals. Data collection parameters are summarized in Table 1.

**Table 1:** Statistics for cryo-EM analyses

	Blm10:13S	Blm10:α-ring	Capless13S (Ump1:α-ring:β2/β3/β4)	5α3βUmp1 (Ump1:α1/α2/α3/ α4/α7:β2/β3/β4)	5α4βUmp1 (Ump1:α1/α2/α3/ α4/α7:β1/β2/β3/β4)
**Data collection and image processing**					
Microscope	Titan Krios				
Camera	K3				
Magnification	81,000				
Voltage (kV)	300				
Electron exposure (e^-^/Å^2^)	60				
Defocus range (µm)	0.9 − 2.5				
Pixel size (Å)	1.068				
Symmetry imposed	C1				
Total micrographs	5,235				
Initial particle images	2,226,993				
Final particle images	586,158	414,121	108,417	78,759	81,760
Map resolution (Å)	2.84	2.75	3.55	3.26	3.11
FSC threshold	0.143	0.143	0.143	0.143	0.143
Map sharpening *B* factor (Å^2^)	−83.8	−77.7	−87.8	−65.20	−75.20
**EMDB code**	46461	46559	46570	46525	46535
**Model building and refinement**					
Initial model used (PDB ID)	7LSX, 4V7O	7LSX, 4V7O	7LSX	7LSX	7LSX
Model resolution (Å)	2.8	2.8	3.7	3.3	2.9
FSC threshold	0.143	0.143	0.143	0.143	0.143
***Model composition***					
Non-hydrogen atoms	68216	59266	35673	29042	31404
Amino acid residues	4297	3731	2289	1859	2008
Protein molecules	12	9	11	9	10
***Real-space correlation***					
CC (volume)	0.70	0.76	0.69	0.74	0.76
CC (mask)	0.70	0.75	0.68	0.73	0.76
***RMS deviations***					
Bond lengths (Å)	0.012	0.012	0.012	0.012	0.011
Bond angles (°)	2.059	2.028	2.038	2.004	1.976
***Validation***					
MolProbity score	1.04	1.01	1.27	1.06	0.83
Clash score	0.60	0.47	0.39	0.48	0.38
Rotamers outliers (%)	1.25	1.28	1.66	1.27	1.11
CaBLAM outliers (%)	1.54	1.85	3.43	1.99	1.56
Cβ outliers (%)	1.53	0.68	0.38	0.46	0.27
***Ramachandran plot***					
Favored (%)	96.12	96.08	92.59	95.53	97.15
Allowed (%)	3.88	3.92	7.41	4.47	2.85
Outliers (%)	0.00	0.00	0.00	0.00	0.00
**PDB code**	9D0T	9D4C	9D4U	9D35	9D3I

### Cryo-EM image processing

All cryo-EM data were processed using cryoSPARC (v.4.4.1)^60^. A data processing flowchart is shown in Supplementary Fig. 1, and a summary of cryo-EM reconstruction statistics is provided in Table 1. A total of 5235 dose-fractionated movies were gain-reference corrected, motion-corrected, dose-weighted, and summed into single-frame micrographs by Patch Motion Correction. Constant transfer function (CTF) parameters were estimated using Patch CTF Estimation. Micrographs were visually inspected, and those exhibiting broken or thick ice, or a CTF-estimated resolution worse than 5 Å were excluded, resulting in a final dataset of 4786 micrographs. An initial set of 589 particles was manually picked from 11 micrographs and subjected to 2D classification. Eighteen well-defined classes were selected to generate templates for automated picking. Template-based particle picking from 232 micrographs identified 234,354 particles, which were further classified to generate improved templates from 28 selected 2D classes. Using these refined templates, a total of 4,058,518 particles were picked from 4786 micrographs and extracted by using a box size of 340 pixels.

Particle stacks were subjected to 2D classification to remove false positive particles, carbon edges, and other junk particles. A total of 2,226,993 particles representing diverse orientations were selected from well-defined 2D classes and re-centered. These particles were subjected to one round of ab initio model generation followed by 3D heterogeneous refinement into 3 classes. One class, comprising 1,462,486 particles, exhibited density corresponding to presumed Blm10 (Class 1 in Supplementary Fig. 1) and was further processed through two additional rounds of ab initio model generation and 3D heterogeneous refinement. Particles were sorted and grouped into Blm10:13S-like or Blm10:α-ring-like populations based on the presence of presumed β2, β3, β4, and Ump1 density. Each group of particles underwent additional 2D classification, CTF refinement and non-uniform refinement, yielding Blm10:13S and Blm10:α-ring reconstructions at overall resolution of 2.84 Å (Blm10:13S with 573,652 particles) and 3.02 Å (Blm10:α-ring with 227,391 particles), respectively, as determined by the gold-standard Fourier shell correlation (GSFSC) in cryoSPARC (Supplementary Fig. 1a). From the first round of 3D heterogeneous refinement, two other classes of particles were identified. One class, consisting of 556,986 particles, displayed a CP-like density map without detectable Blm10 density (Class 2 of Supplementary Fig.1). These particles were subjected to similar ab initio model generation, 3D heterogeneous refinement, CTF refinement and non-uniform refinement. Three maps were reconstructed corresponding to 7α3βUmp1, 5α3βUmp1, and 5α4βUmp1 assemblies, with overall resolution of 3.55 Å, 3.26 Å, and 3.11 Å, respectively (Supplementary Fig. 1b). The remaining 207,611 particles (Class 3 of Supplementary Fig. 1) were processed using the same refinement strategy; however, the resulting maps were resolved at >5.7 Å and were not analyzed further.

In parallel with the first round of 3D heterogeneous refinement, the full set of 2,226,993 particles was subjected to one round of ab initio model generation followed by 3D homogeneous refinement, yielding a reconstruction of 2.85 Å resolution. Subsequently, unsupervised 3D classification was performed, sorting the particles into 6 classes. One class, comprising 414,121 particles, was distinct from the others and exhibited density corresponding to only a single β subunit. This particle subset was further processed by CTF refinement and non-uniform refinement, resulting in a reconstructed Blm10:α-ring map at an overall resolution of 2.75 Å (Supplementary Fig. 1d). This Blm10:α-ring map was used for subsequent model building and refinement. All GSFSC curves, viewing direction distribution plots, and local resolution maps were generated in cryoSPARC.

### Model building and structure analyses

For atomic model building and refinement, the Blm10 structure from PDB 4V7O and α1–α7, β2–β4, Ump1 structures from PDB 7LSX were fitted as rigid bodies into the Blm10:13S density map using UCSF ChimeraX (v.1.9)^61^. To assist model interpretation, the refined Blm10:13S map, along with other reconstructed maps, was processed using EMready (v.2.0)^62^. The Blm10:13S structural model was refined through iterative cycles of manual model building in Coot (v.0.9.8.93 EL)^63^, followed by real-space refinement in Phenix (v.1.21.2)^64^. Model geometry and real-space correlation were validated using the *phenix.validation_cryoem* module in Phenix (v.1.21.2)^64^. The refined Blm10:13S atomic model was subsequently used as the initial model for atomic model building and refinement of Blm10:α-ring, 7α3βUmp1, 5α3βUmp1, and 5α4βUmp1 complexes. For 5α4β:Ump1, the β1 structure from PDB 4V7O was additionally fitted as a rigid body into the density map. A summary of model building and validation statistics is provided in Table 1. All structural figures were prepared using UCSF ChimeraX (v.1.9)^61^.

### Reporting summary

Further information on research design is available in the Nature Portfolio Reporting Summary linked to this article.

## Supplementary information

Supplementary Information

Reporting Summary

Transparent Peer Review file

## Source data

Source Data

## Data Availability

The cryo-EM maps generated by this study have been deposited into the Electron Microscopy Data Bank (EMDB) under accession codes EMD-46461 (Blm10:13S), EMD-46559 (Blm10:α-ring), EMD-46570 (7α3βUmp1), EMD-46525 (5α3βUmp1), and EMD-46535 (5α4βUmp1). The corresponding atomic models have been deposited into the Protein Data Bank (PDB) under accession codes 9D0T (Blm10:13S), 9D4C (Blm10:α-ring), 9D4U (7α3βUmp1), 9D35 (5α3βUmp1), and 9D3I (5α4βUmp1). The raw micrographs and particle stacks have been deposited in the Electron Microscopy Public Image Archive (EMPIAR) under accession number EMPIAR-12275. Source data are provided with this paper. All other data supporting the findings of this study are available from the corresponding authors upon request. Source data are provided with this paper.
