# Supplementary material for: Structure of Blm10:13S proteasome intermediate reveals parallel assembly pathways for the proteasome core particle
Source: Nat Commun. 2026 Sep 21;17:10993. doi: 10.1038/s41467-026-77943-w (PMC13683372; doi:10.1038/s41467-026-77943-w)
Supplement: Supplementary file 1 [file 41467_2026_77943_MOESM1_ESM.pdf]

Structure of Blm10:13S proteasome intermediate reveals parallel assembly pathways for the proteasome core particle

Mandeep Kaur[#,1], Xiang Chen[#,2], Stella Y. Lee[1], Tyler M. Weaver[1,3], Bret D. Freudenthal[1], Kylie J. Walters[*,2], and Jeroen Roelofs[*,1]

[1]Department of Biochemistry and Molecular Biology, University of Kansas Medical Center, Kansas City, 3901 Rainbow Blvd., HLSIC 1077, Kansas, USA

[2]Protein Processing Section, Center for Structural Biology, Center for Cancer Research, National Cancer Institute, National Institutes of Health, Frederick, MD, USA.

[3] Current address: Department of Biochemistry and Molecular Genetics, University of Virginia School of Medicine, Charlottesville, Virginia 22908, USA

[#]These authors contributed equally

[*]correspondence: kylie.walters@nih.gov or jroelofs@kumc.edu

Running title: Parallel Assembly Pathways for Proteasome Core Particle

Supplementary Figure 1-9

Supplementary tables 1-3

Source Data – supplementary figure immunoblotting

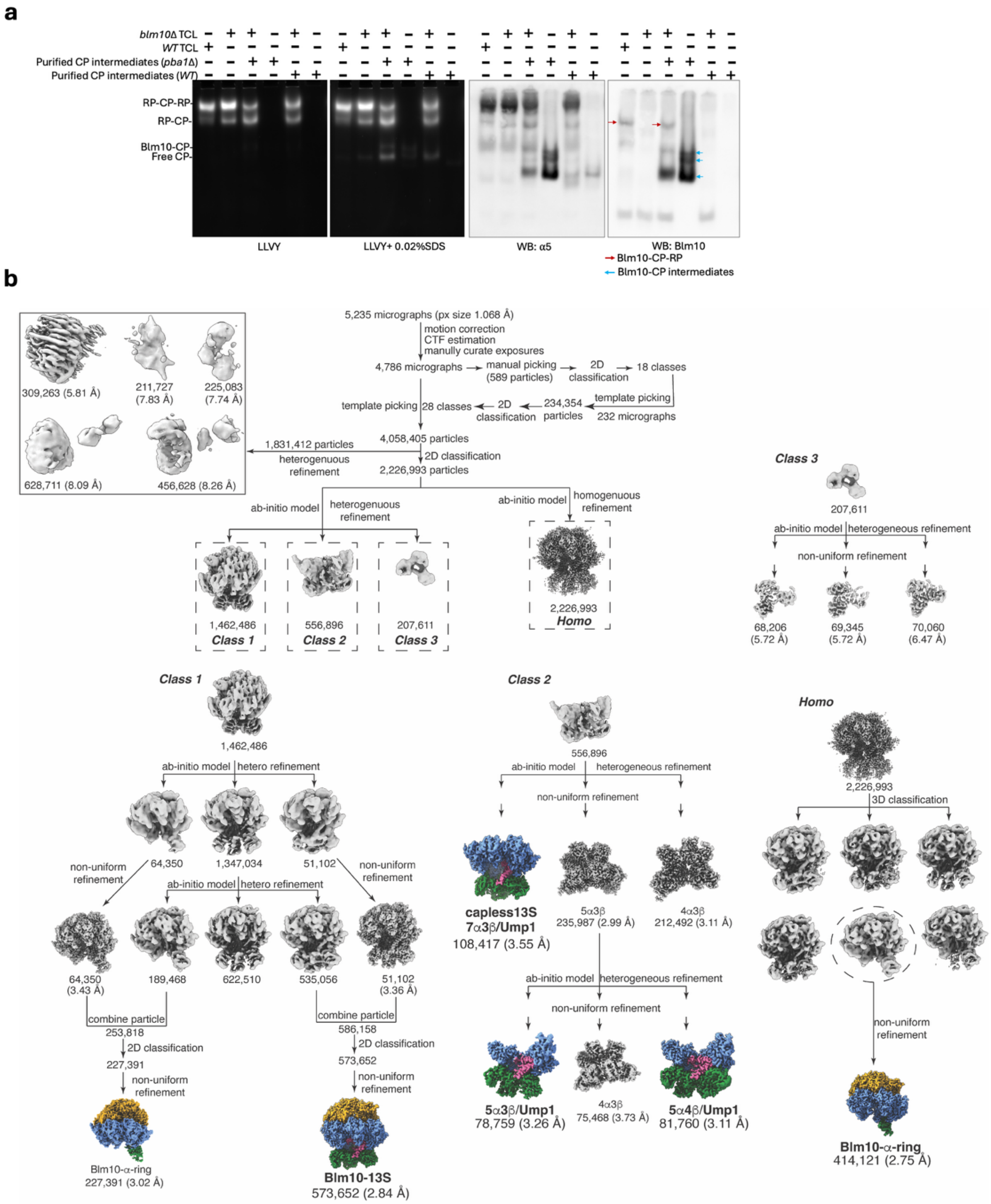

Supplementary Fig. 1. Reconstitution and cryo-EM workflow for complexes purified using an Ump1-affinity tag. **a**, Reconstitution of purified Blm10-bound CP intermediates with total cell lysates (TCL) leads to formation of mature Blm10:CP-RP complexes.

Purified CP intermediates from wild-type or *pba1*Δ strains were incubated with cleared total lysate of wild-type or *blm10*Δ strains for 30 mins at 30 °C. Next, samples were separated on native PAGE, followed by in-gel peptidase activity assay. The gels were transferred onto PVDF membrane and blotted for α5 and Blm10. Lane 4 in the right two panels show the purified assembly intermediate from *pba1*Δ (similar to the preparation used for cryo-EM structure determination). In lane 3, the sample from lane 4 was mixed with lysate from *blm10*Δ cells and the formation of Blm10 positive slower migrating bands indicates the formation of Blm10:CP and Blm10:CP-RP complexes (indicated by a red arrow). These results suggest that the Blm10-associated assembly intermediates (Blm10:13S) are functional and can form mature Blm10-bound CP complexes upon reconstitution with TCL from the *blm10*Δ strain. The experiment was repeated two times. **b,** Cryo-EM data processing workflow for complexes purified using an Ump1-affinity tag. Processing scheme for the classification illustrates reconstructions obtained following both homogeneous and heterogeneous refinement of Blm10:13S, Blm10:α-ring, 7α3βUmp1, 5α3βUmp1, and 5α4βUmp1 complexes. The reconstructed maps are colored as in Fig. 1.

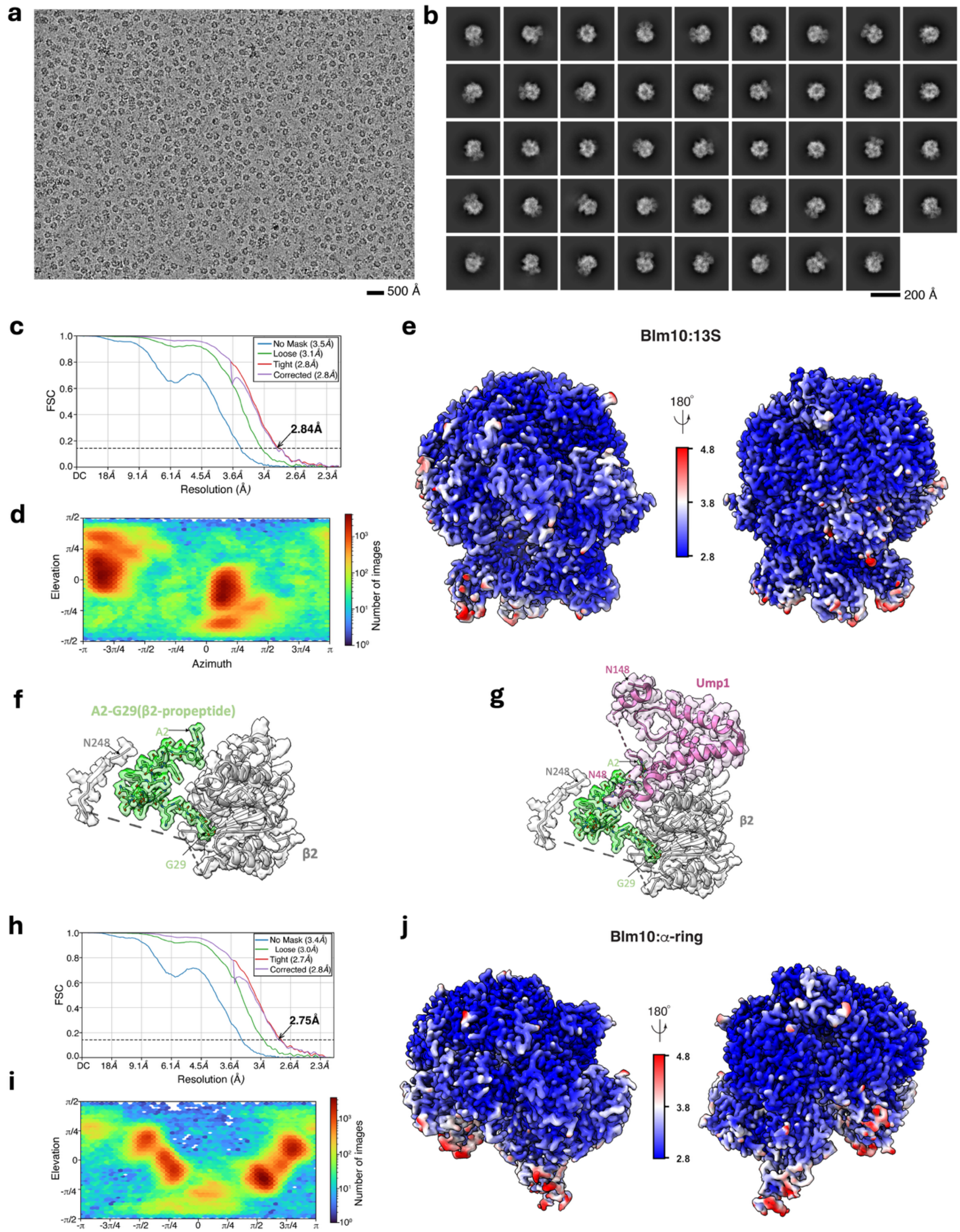

Supplementary Fig. 2. Cryo-EM data analyses of the Blm10:13S and Blm10:α-ring complexes. a, Representative cryo-EM micrograph acquired on the Ump1-tagged

sample. Data were collected on a Titan Krios G3i transmission electron microscope equipped with a Gatan K3 direct detector and energy filter. Scale bar, 500 Å. **b,** Representative 2D class averages generated in cryoSPARC [60]. Scale bar, 200 Å. **c-e and h-j,** Gold-standard Fourier Shell correlation (GSFSC) curves (c and h), viewing direction distribution plots (d and i), and cryo-EM density maps colored by local resolution as calculated in cryoSPARC (e and j) are shown for the refined reconstruction maps of the Blm10:13S (c - e) or Blm10:α-ring (h - j) complexes. In panel c and h, the FSC value of 0.143 is indicated by a black dashed line. **f,** Cryo-EM density of the β2 subunit in Blm10:13S with the model structure showing the propeptide (residues A2-G29) in green and the remainder of the subunit in grey. **g,** Cryo-EM density of Ump1 (pink) and the β2 subunit (colored as in panel f) in Blm10:13S with the model structure, illustrating the orientation of Ump1 relative to the β2 subunit.

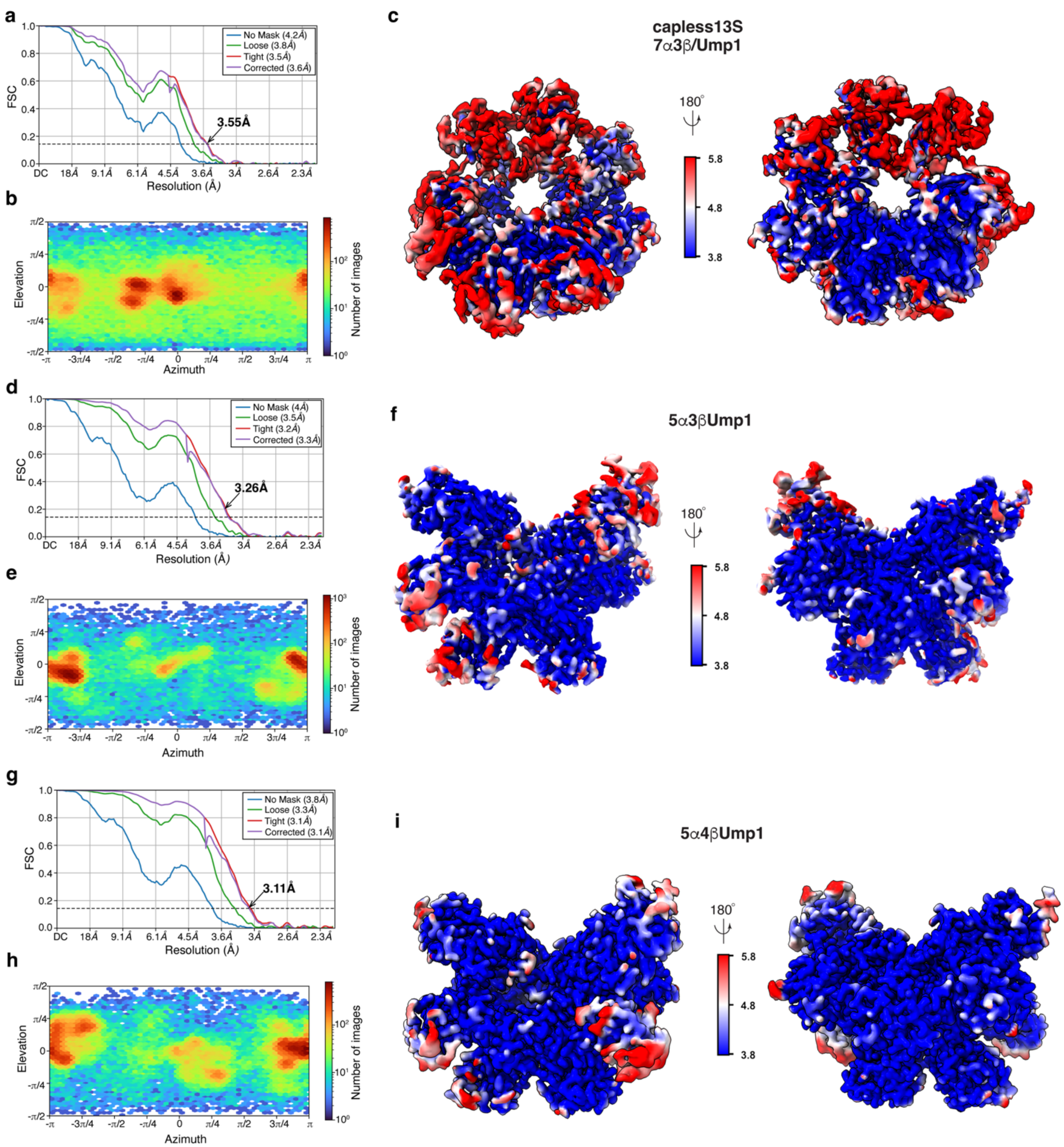

Supplementary Fig. 3. Cryo-EM data analyses of the capless13S and partial α-ring complexes. a-i, GSFSC curves (a, d, and g), viewing direction distribution plots (b, e, and h), and cryo-EM density maps colored by local resolution as calculated in cryoSPARC (c, f, and i) are shown for the refined reconstruction maps of capless13S (a - c), 5α3βUmp1 (d - f), and 5α4βUm (g - i). The FSC value of 0.143 is indicated by a black dashed line in a, d, and g.

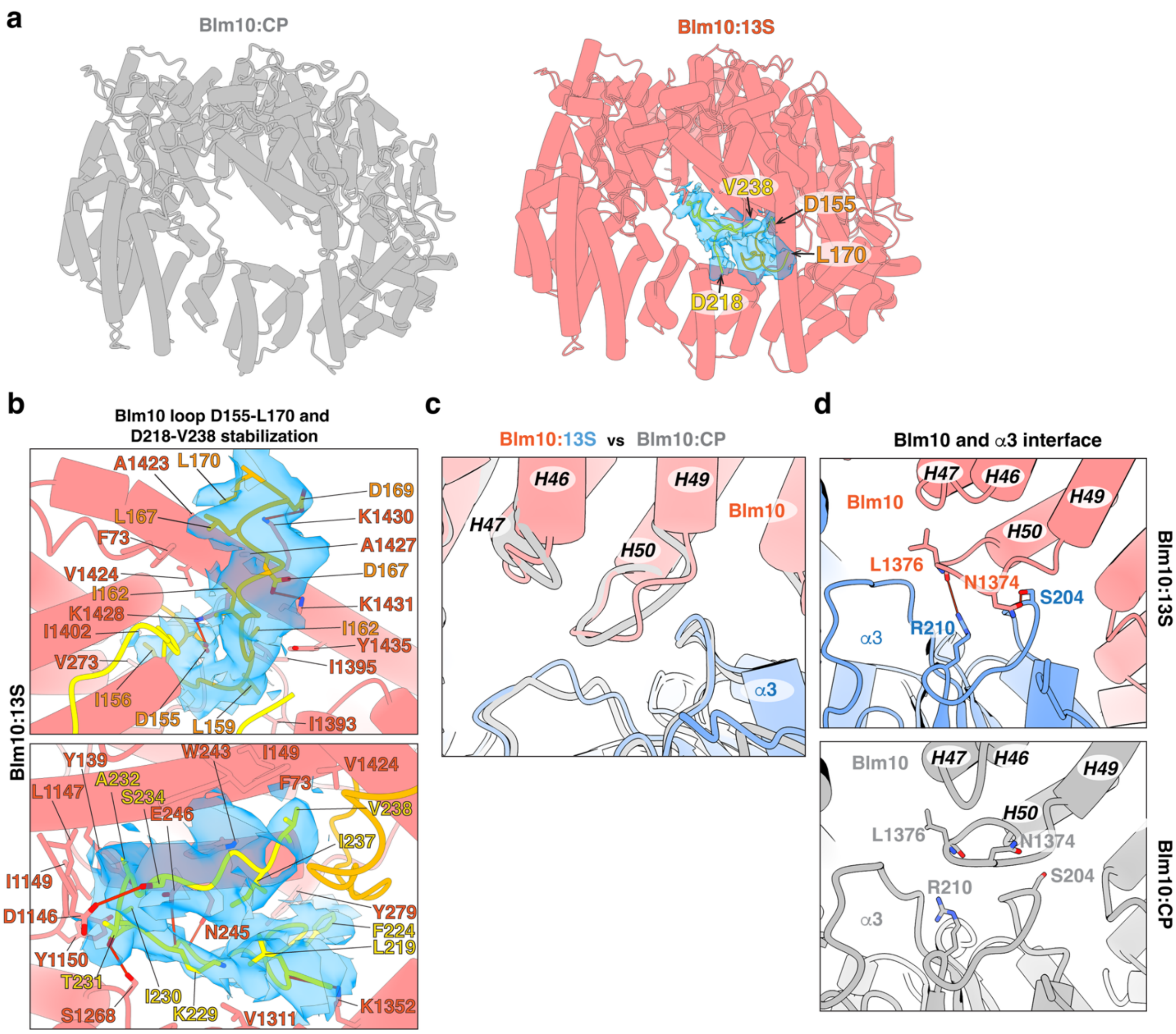

Supplementary Fig. 4. Conformational changes impacting Blm10 dome opening. a, Top view of side-by-side Blm10 cartoon structures from Blm10:CP (grey, left; PDB: 4V7O) and Blm10:13S (pink, right). The Blm10 loops resolved in Blm10:13S (residues D155-D166 and G221-V238) are highlighted in orange and yellow, respectively. Corresponding electron densities for Blm10 loops from the unsharpened Blm10:13S map (blue) is shown alongside the structural model. These loops are not resolved in the Blm10:CP structure (PDB:4V7O). **b**, Expanded cartoon view of the Blm10:13S structure highlighting intramolecular interactions involving the Blm10 loops D155-D166 (orange, top) and G221-V238 (yellow, bottom). The residues participating in hydrogen bonding or hydrophobic interactions are shown as sticks, with nitrogen and oxygen atoms colored indigo and red, respectively. Corresponding electron densities for Blm10 loops from the unsharpened

Blm10:13S map (blue) is shown alongside the structural model. **c**, Expanded view of the superimposed Blm10 (pink):13S (blue) and Blm10:CP (grey; PDB:4V7O) cartoon structures highlighting the rearrangement of the loops connecting Blm10 helices H46 to H47 and H49 to H50. These loops are positioned at the Blm10 (pink):α3 (blue) interface. **d**, Expanded cartoon view of the Blm10:13S structure (top) showing additional interactions between Blm10 and α3 that are absent in the Blm10:CP structure (bottom). Blm10 and α3 are colored as in (c). Interacting residues in Blm10 and α3 are shown as sticks, with nitrogen, oxygen and sulphur atoms colored indigo, red, and yellow, respectively. In panels b and d, hydrogen bonds and hydrophobic interactions are indicated by red and black lines, respectively. In panels b-d, Blm10 helices H4-H6, H46-H47, H49-H50, and H52 are labeled.

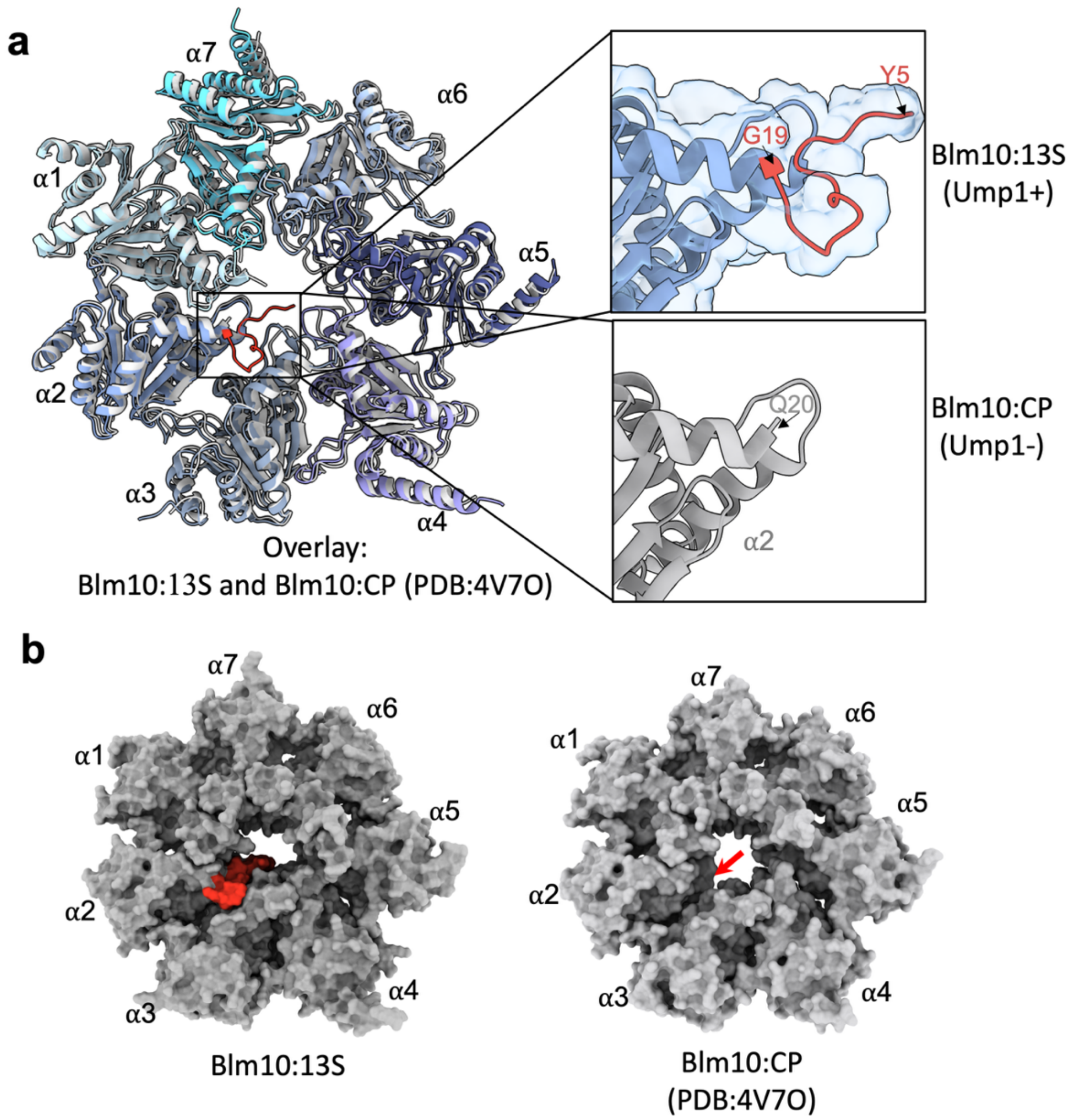

Supplementary Fig. 5. A stabilized α2 N-terminus restricts gate opening in Blm10:13S compared to Blm10:CP. a, Top views of the α-ring from Blm10:13S (shades of blue and purple) and Blm10:CP (shades of gray; PDB: 4V7O) are shown. Expanded views (right) highlight the more ordered α2 N-terminus (residues Y5-G19, red) of Blm10:13S (top), compared with Blm10:CP (bottom), in which the first resolved residue is Q20 (grey). Corresponding electron densities from the unsharpened Blm10:13S map (blue) is shown alongside the structural model. **b,** Top views of the α-ring molecular surface illustrating the CP gate architecture in Blm10:13S (left) and Blm10:CP (right). The stabilized α2 N-terminus (S8-G19, red, left) is highlighted in Blm10:13S. A red arrow indicates the position of the α2 N-terminus in Blm10:13S.

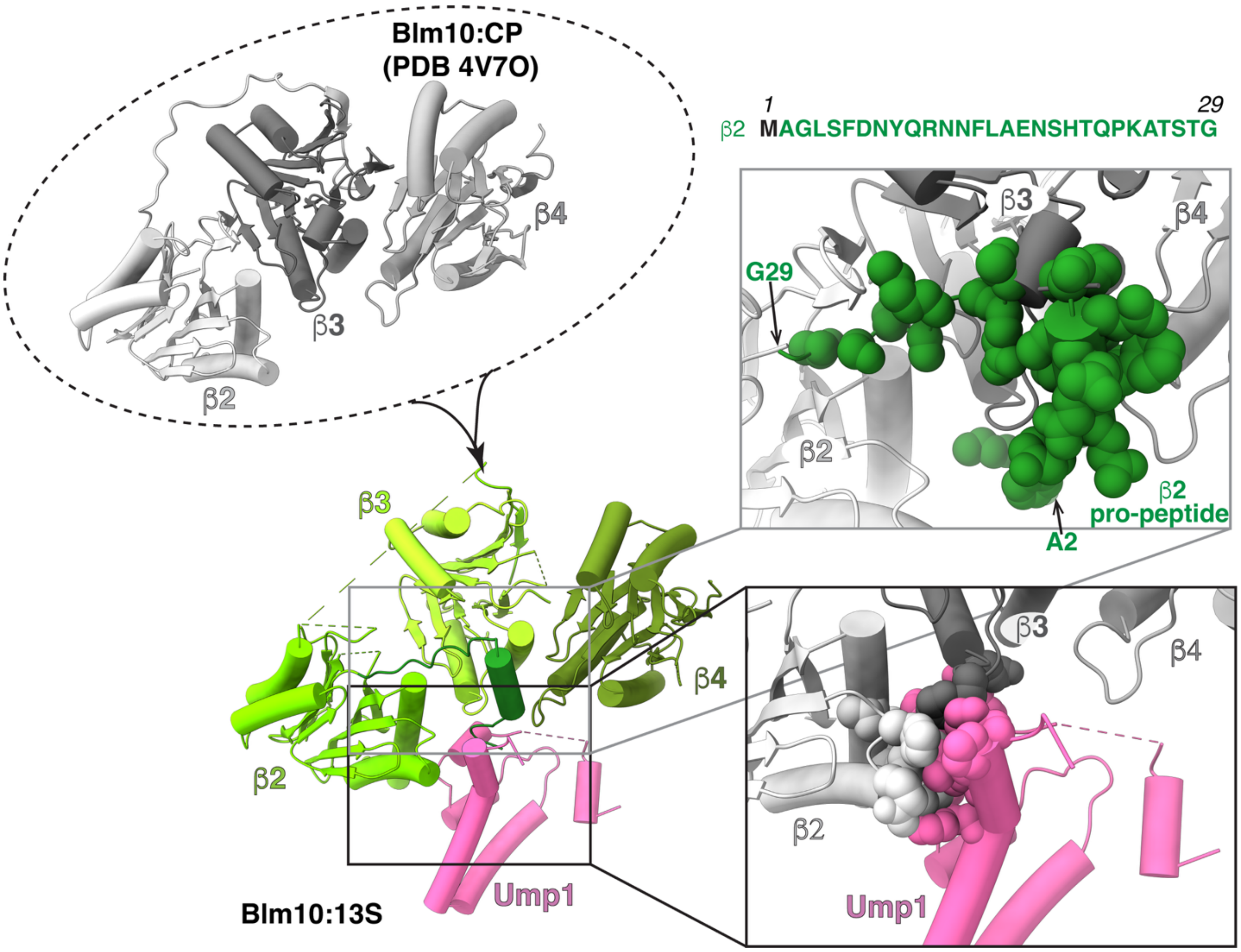

Supplementary Fig. 6. Steric clashes involving Ump1 upon alignment of Blm10:13S and Blm10:CP reveal conformational changes during CP maturation. Superposition of the β-ring subunits (β2, β3, and β4) from the Blm10:CP structure (top left, shades of grey; PDB: 4V7O) with the corresponding subunits in the Blm10:13S structure (bottom left, shades of green) is shown. Expanded views (top and bottom right) highlight the rearrangements of β2 and β3 that prevent steric clashes between β2 and Ump1 (pink), as well as between β3 and the β2 propeptide (dark green). The residue atoms of β2 propeptide (top, right), β2 and Ump1 (bottom, right) involved in steric clashes are displayed as spheres. The amino acid sequence of the β2 pro-peptide is also shown.

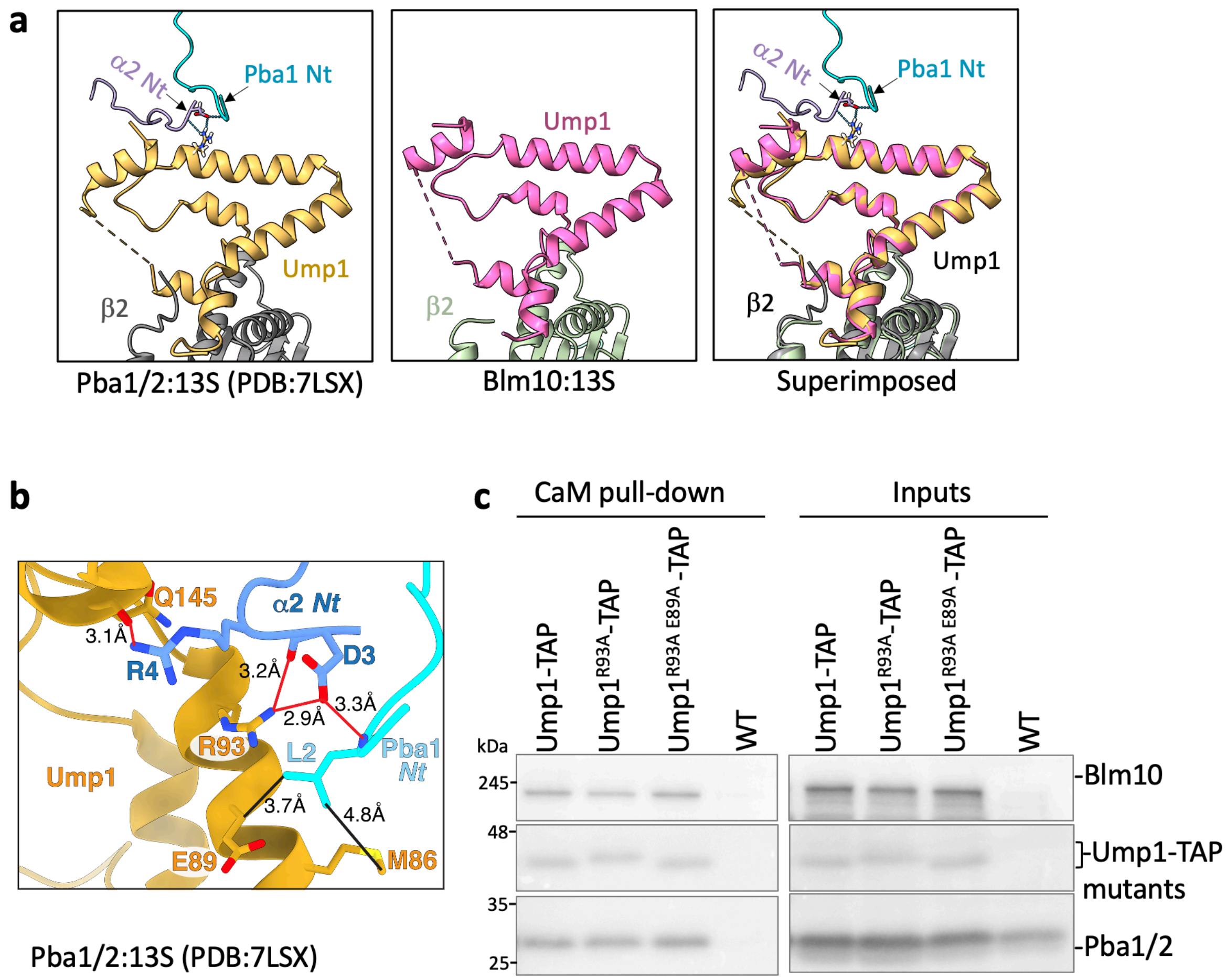

Supplementary Fig. 7. Disruption of putative Pba1/Ump1 interaction does not impair Pba1 binding to Pba1/2:13S. a, The position of Ump1 in Pba1/2:13S and Blm10:13S remains unchanged. The left panel shows Ump1 (yellow) interacting with the N-termini of Pba1 (cyan) and α2 (purple), with hydrogen bonds indicated by blue dashed lines. The middle panel shows Ump1 (pink) aligned within the Blm10:13S complex, and the right panel presents a superposition of Pba1/2:13S (PDB:7LSX) and Blm10:13S, highlighting the conserved positioning of Ump1. **b,** Residues involved in the interaction between N-terminus of Pba1, the α2 subunit, and Ump1 in Pba1/2:13S (PDB: 7LSX) are shown. Hydrogen bond are indicated by red lines and hydrophobic interactions by black lines, with distances displayed in Å. **c,** Western blot analysis of Ump1-TAP CaM based pull down from mutant strains Ump1^{R93A} and Ump1$^{R93A\ E89A}$, along with the corresponding inputs. Experiment was repeated two times with similar results.

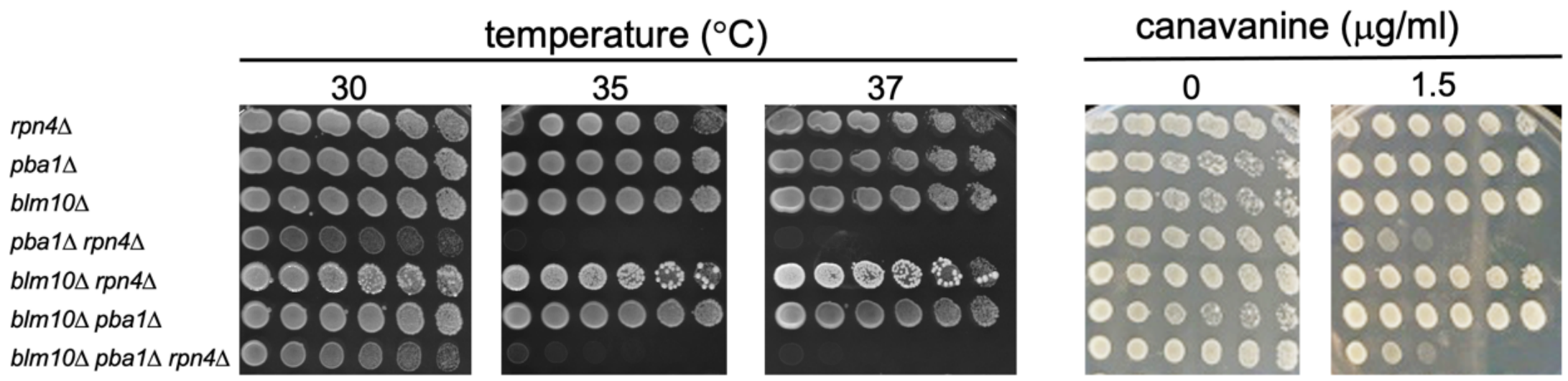

Supplementary Fig. 8. No synergetic effect between PBA1 and BLM10 in the presence or absence of RPN4. Indicated strains were monitored for several days to determine impact of genetic manipulations on growth under heat stress and the arginine analog canavanine using 4-fold serial dilutions. Rpn4 is a transcription factor for proteasomes and can mask assembly phenotypes through the upregulation of expression of proteasome subunits. If the parallel CP assembly pathways mediated by Blm10 and Pba1 were redundant or synergistic for the formation of CP, we hypothesized that the combined deletion, especially in the *rpn4*Δ background should show a more severe phenotype. The lack of an additive effect of the deletion of Blm10 in the *pba1*Δ *rpn4*Δ background suggests that these pathways do not exist to provide more robust CP formation.

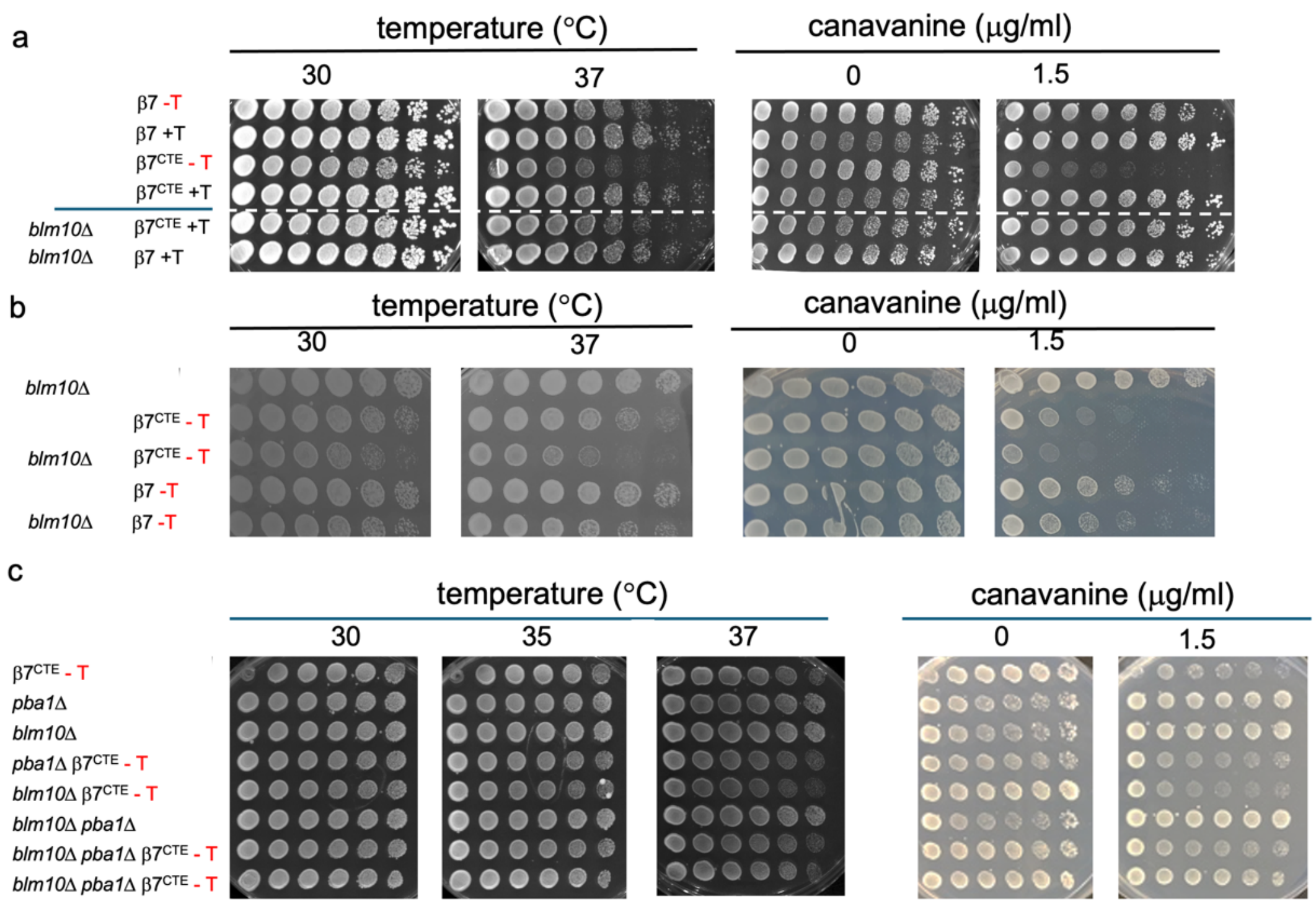

Supplementary Fig. 9. β7^{CTE} phenotype exacerbated by the absence of transcriptional terminator and no synergy between BLM10 and PBA1. a, Indicated strains were monitored for several days to determine impact of genetic manipulations on growth under heat stress and the arginine analog canavanine using 4-fold serial dilutions. Plates were incubated for 2-5 days at 30 °C or indicated temperature before imaging. In contrast to previous publications, introducing the β7 CTE mutation (deletion of last 19 amino acids) into the *blm10*Δ background did not yield an appreciable phenotype in our background (row 2, 5, and 6). However, in previous study a transcriptional terminator appeared to be disrupted upon introducing the β7 truncation. We had previously observed that for different genes in our background such absence contributed to phenotypes presumably due to reduced proteins levels due to improper mRNA formation. -T truncation was introduced into the endogenous locus by deletion of part of the ORF and insertion of a selection cassette without introducing a replacement transcriptional terminator. +T strains introduced the truncation together with a heterologous transcriptional terminator behind the truncation β7. -T shows a modest growth defect that

was enhanced by the truncation. **b,** Deletion of BLM10 further exacerbates the sensitivity of β7 mutants for heat stress and canavanine induced protein misfolding. **c,** $\beta7^{CTE}$ -T was combined with deletion of BLM10 and/or PBA1 to determine occurrence of synergy. In our strain background, the $\beta7^{CTE}$ -T mutation shows a subtle phenotype when combined with *blm10*Δ but not *pba1*Δ, especially when comparing the difference in growth for each strain with and without stress. The combined deletion of both BLM10 and PBA1 in the $\beta7^{CTE}$ -T did not further exacerbate the phenotype, suggesting Blm10 and Pba1 work in non-synergistic parallel assembly pathways.

Supplementary Table 1. Strain list

Strain	Genes manipulated	Repair DNA	Fig.	Ref.
sUB61	*MATα lys2-801 leu2-3, 2-112 ura3-52 his3Δ200 trp1-1*	n/a	1a, 5e,6b-c, ED7	(a)
sJR288	*MATA rpn4::KAN*	n/a	ED8	(d)
sJR395	*MATα blm10::NAT*	n/a	1a, ED8, 9	(d)
			ED8	
sJR785	*MAT A ump1::UMP1-CBP-TEV-ZZ-(His3MX6)*	n/a	5e,6b-c, ED7	(b)
sJR792	*MAT A pba1::NAT ump1::UMP1- CBP-TEV-ZZ- (His3MX6)*	n/a	6c	(c)
sJR793	*MAT A pba1::HYG blm10::NAT ump1::UMP1- CBPTEV-ZZ-(His3MX6)*	n/a	6c	(c)
sJR794	*MAT A blm10::NAT ump1::UMP1- CBPTEV-ZZ-(His3MX6)*	n/a	6c	(c)
sJR795	*MAT A pba1::PBA1Δ3C (KAN) ump1::UMP1- CBPTEV-ZZ-(His3MX6)*	n/a	5e	(c)
sJR809	*MAT A pba1::PBA1 Δ3C (KAN) pba2:: PBA2 Δ3C (NAT) ump1::UMP1- CBPTEV-ZZ-(His3MX6)*	n/a	5e	(c)
sJR857	*MATα pba1::HYG*	n/a	1a, ED8, 9	(c)
sJR2578	*MAT A pba1::PBA1^{L2S}Δ3-17 ump1::UMP1-CBP-TEV-ZZ-(His3MX6)*	pRL1478	5e	This study
sJR2583	*MAT A pba1::PBA1 ^{L2S}Δ3-31 ump1::UMP1-CBP-TEV-ZZ-(His3MX6)*	pRL1503	5e	This study
sJR2584	*MAT A pba1::PBA1 ^{L2S}Δ3-31 pba1::PBA1Δ3C (KAN) ump1::UMP1-CBP-TEV-ZZ-(His3MX6)*	pRL1503	5e	This study
sJR2586	*MATA pba1::PBA1 ^{L2S}Δ3-31 pba1::PBA1Δ3C (KAN) pba2:: PBA2 Δ3C (NAT) ump1::UMP1-CBP-TEV-ZZ-(His3MX6)*	pRL1503	5e	This study
sJR2588	*MATA pba1::PBA1^{L2S}Δ3-17 blm10::GPDpGFPBLM10 (CloNAT) ump1::UMP1-CBP-TEV-ZZ-(His3MX6)*	pRL1478	5e	This study
sJR2589	*MATA pba1::PBA1 ^{L2S}Δ3-31 blm10::GPDpGFPBLM10 (CloNAT) ump1::UMP1-CBP-TEV-ZZ-(His3MX6)*	pRL1503	5e	This study
sJR1012	*MATA blm10::GPDpGFPBLM10 (NAT) ump1::UMP1-CBP-TEV-ZZ-(His3MX6)*	n/a	5e	(d)
sJR2614	*MATA pba4::PBA4-CBP-TEV-ZZ-(His3MX6)*	n/a	6b	(b)
sJR2543	*MATA ump1::UMP1^{R93A} ump1::UMP1- CBPTEV-ZZ-(His3MX6)*	pRL1470	ED7	This study
sJR2545	*MATA ump1::UMP1$^{R93A, G89A}$ ump1::UMP1- CBPTEV-ZZ-(His3MX6)*	pRL1471	ED7	This study
sJR2750	*MATA pre4::PRE4(KAN)*	n/a	ED9	This study
sJR2751	*MATA pre4::PRE4$^{no\ terminator}$ (KAN)*	n/a	ED9	This study
sJR2752	*MATA pre4::PRE4^{CTE}(KAN)*	n/a	ED9	This study
sJR2753	*MATA pre4::PRE4$^{CTE,\ no\ terminator}$ (KAN)*	n/a	ED9	This study
sJR2754	*MATα pre4::PRE4(KAN) blm10::NAT*	n/a	ED9	This study
sJR2755	*MATα pre4::PRE4^{CTE}(KAN) blm10::NAT*	n/a	ED9	This study
sJR2757	*MATα pre4::PRE4$^{no\ terminator}$ (KAN) blm10::NAT*	n/a	ED9	This study
sJR2756	*MATα pre4::PRE4$^{CTE,\ no\ terminator}$ (KAN) blm10::NAT*	n/a	ED9	This study
sJR2765	*MATa pre4::PRE4$^{CTE,\ no\ terminator}$ (KAN) pba1::HYG*	n/a	ED9	This study
sJR2766	*MATa blm10::NAT rpn4::KAN*	n/a	ED8	This study
sJR2768	*MATa pba1::HYG rpn4::KAN*	n/a	ED8	This study
sJR2782	*MATa blm10::NAT pba1::HYG*	n/a	ED8,9	This study
sJR2784	*MATA blm10::NAT pba1::HYG rpn4::KAN*	n/a	ED8	This study
sJR2786	*MATA pre4::PRE4$^{CTE,\ no\ terminator}$ (KAN) blm10::NAT pba1::HYG*	n/a	ED9	This study
sJR2787	*MATα pre4::PRE4$^{CTE,\ no\ terminator}$ (KAN) blm10::NAT pba1::HYG*	n/a	ED9	This study

genotype is: ***MAT A his3Δ0*** *leuΔ0 met15Δ0 ura3Δ0,* except for sUB61 which is *MATα lys2-801 leu2-3, 2-112 ura3-52 his3D200 trp1-1.* (a) Finley, D., Ozkaynak, E. & Varshavsky, A. **1987** Cell 48, 1035-1046. (b) Ghaemmaghami, S. et al. **2003** Nature 425, 737-741. (c) Wani, P. et al. **2015** Nature Communications 7384, 1-11. (d) Burris, A. et al 2021 Journal of Biological Chemistry, 296, 1-18.

Supplementary Table 2. Crispr/Cas9 gRNA and repair DNA

Target gene	gRNA	Plasmid with gRNA & Cas9	Repair DNA	Sequence repair DNA
UMP1	TACTACCGTTTGTTGAAAGG	pJR1162	pRL1470	AGCAAGAAGGCGGTGCAGTACCTCTTTCCACACAATTGAATGACAGACATCCATTAGAATCAACATTGAAGAACTGGGAAACCACACAGCGCCAAAGACAAATGGAACAGTATCGACAAATATTTGGCATTGCCGAACCAATGAAgAGaACtATGGAgATGGAAATCGTCAATgcaACCGACTTCAAtCCaCTgTCtACcAACGGTAGTATACACCGTGACATACTACTGAACAAAGAGTGCAGCATCGATTGGGAGGACGTCTACCCTGGTACTGGCCTACAAGCCAGCACCATGGTAGGTGATGACGTCCACAGC
UMP1	TACTACCGTTTGTTGAAAGG	pJR1162	pRL1471	AGCAAGAAGGCGGTGCAGTACCTCTTTCCACACAATTGAATGACAGACATCCATTAGAATCAACATTGAAGAACTGGGAAACCACACAGCGCCAAAGACAAATGGAACAGTATCGACAAATATTTGGCATTGCCGAACCAATGAAgAGaACtATGGAgATGGcAATCGTCAATgcaACCGACTTCAAtCCaCTgTCtACcAACGGTAGTATACACCGTGACATACTACTGAACAAAGAGTGCAGCATCGATTGGGAGGACGTCTACCCTGGTACTGGCCTACAAGCCAGCACCATGGTAGGTGATGACGTCCACAGC
PBA1	CTACTCTAGCAAAATATGAT	pJR1164	pRL1478	TTGGCACCTAATTCAGACATTTTTGCTTTTGTTCCTCCACTCAAATGTAGTCTTTTTCAATTCTGAAGTGGGATTATTTATAGATGACGTTTGACTTGATGAAGTTTTATTGCCTTTACTTTTTTTTTTCTTTCGCATCAAAATTTCAGACTTCATTGAAATCAATTTAAGAAGTTGCGTGGCAATAGGTGGCAACAGGCGAAATAATAAGAAACAGCGACCAAGATATCATCGCACTACAGTAAAATTTTCATTTATAGCGATGagtCTCCCAGAGATTTCAAAAAACCTGCAATCTTTAGAAGTCTGCCCGGTTCCAAAAGTTGAGTTTCCTCAGGACTTGGATGTT
PBA1	CTACTCTAGCAAAATATGAT	pJR1164	pRL1503	TTGGCACCTAATTCAGACATTTTTGCTTTTGTTCCTCCACTCAAATGTAGTCTTTTTCAATTCTGAAGTGGGATTATTTATAGATGACGTTTGACTTGATGAAGTTTTATTGCCTTTACTTTTTTTTTTCTTTCGCATCAAAATTTCAGACTTCATTGAAATCAATTTAAGAAGTTGCGTGGCAATAGGTGGCAACAGGCGAAATAATAAGAAACAGCGACCAAGATATCATCGCACTACAGTAAAATTTTCATTTATAGCGATGagtCCGGTTCCAAAAGTTGAGTTTCCTCAGGACTTGGATGTTCCTCAATATTCGACAGCCGTCATTACTACTAAGATAATGAACCCATTATTCCCCAAAAATCTGCTTCAATTAACCTCTATTGGTGAAATTAAAACTACATTGACGGTGAAAAGTCCAAGTCTTCCCCAATCTTCAGGTAAACACTCGTGG

Supplementary Table 3. PCRs for PRE4 integration material and screening

	Primer 1	Primer 2	template	Screening size
PRE4^{CTE}*(KAN)*	pRL1664 (AGA ACA CAG GTT TAA CTT TCA AGA AAA ACT TGC AAG TTT AGC GCC ACT TCT AAA TAA GCG)	pRL1668 (AAA AAT AAA ATG AGT ATT TAA TGA AGG TGG GAT AAA ACT CAT CGA TGA ATT CGA GCT CG)	pYM13 (Janke et al, Yeast, 2004)	
PRE4$^{CTE,\ no\ terminator}$ *(KAN)*	pRL1665 (AAG AAC ACA GGT TTA ACT TTC AAG AAA AAC TTG CAA GTT TAG CGT ACG CTG CAG GTC GAC)	pRL1668 (AAA AAT AAA ATG AGT ATT TAA TGA AGG TGG GAT AAA ACT CAT CGA TGA ATT CGA GCT CG)	pFA6a-kanMX6 (Bahler et al, Yeast, 1998)	
PRE4(KAN)	pRL1666 (CGC CAA GGA TAT TAA AGG CTA CGG TAC TCA AAA AAT TTA GCG CCA CTT CTA AAT AAG CG)	pRL1668 (AAA AAT AAA ATG AGT ATT TAA TGA AGG TGG GAT AAA ACT CAT CGA TGA ATT CGA GCT CG)	pYM13 (Janke et al, Yeast, 2004)	
PRE4$^{no\ terminator}$ *(KAN)*	pRL1667 (CGC CAA GGA TAT TAA AGG CTA CGG TAC TCA AAA AAT TTA GCG TAC GCT GCA GGT CGA C)	pRL1668 (AAA AAT AAA ATG AGT ATT TAA TGA AGG TGG GAT AAA ACT CAT CGA TGA ATT CGA GCT CG)	pFA6a-kanMX6 (Bahler et al, Yeast, 1998)	
**Screening primers** pRL1669 (CCA CTG TAC AGG TTG CTG) pRL1700(AGC GGT GGT GCA TGG TG), and pR714(GCG ACA GTC ACA TCA TGC)				
wild type				389 bp
PRE4^{CTE}*(KAN)*				490 bp
PRE4$^{CTE,\ no\ terminator}$ *(KAN)*				302 bp
PRE4(KAN)				550 bp
PRE4$^{no\ terminator}$ *(KAN)*				362 bp

Source Data – supplementary figure immunoblotting

Red dashed boxes provides indication of cropping used in manuscript

Supplementary Figure 7

Blotting: membrane was cut in two parts;
top was blotted for Blm10, bottom for Pba1/2

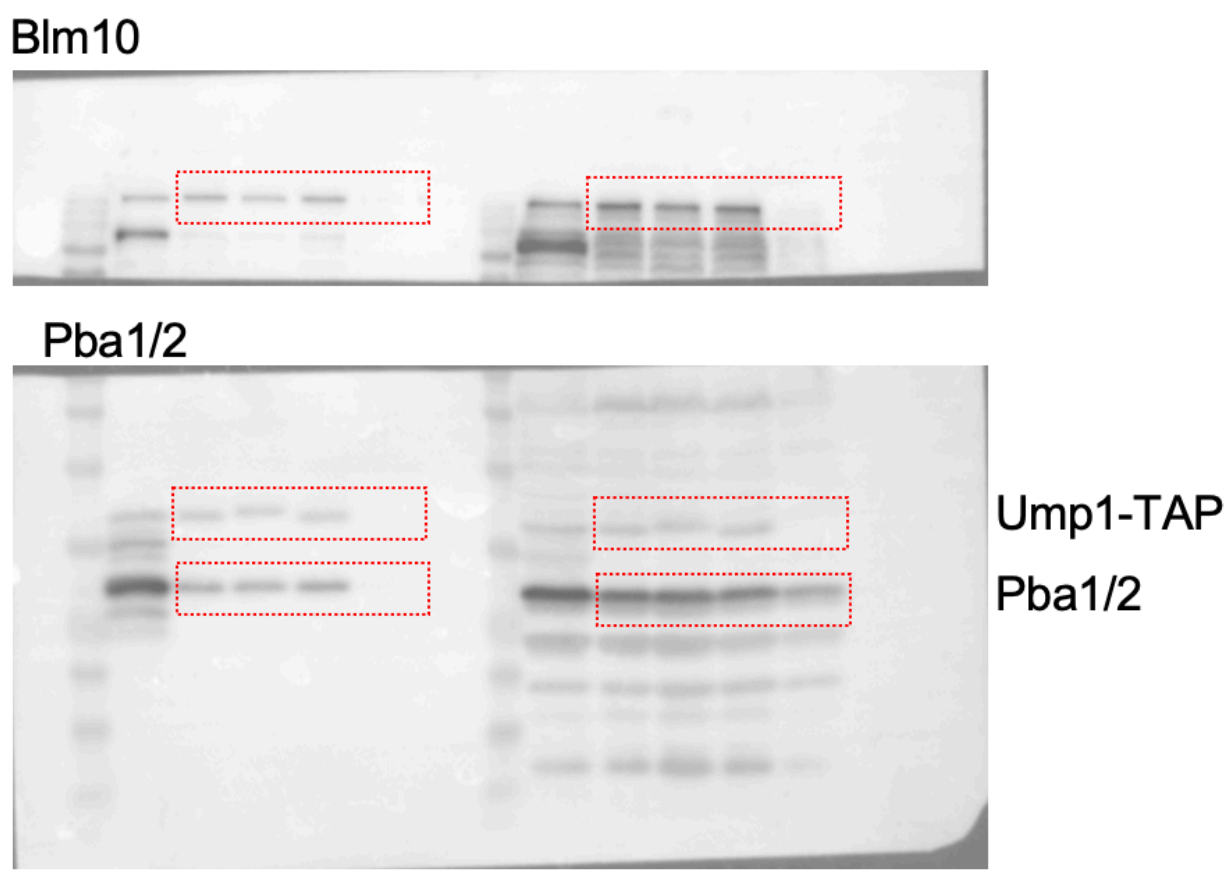

Note: Secondary antibody (rabbit, poly) binds to proA-tag on Ump1
