# Supplementary material for: Structure of Blm10:13S proteasome intermediate reveals parallel assembly pathways for the proteasome core particle
Source: Nat Commun. 2026 Sep 21;17:10993. doi: 10.1038/s41467-026-77943-w (PMC13683372; doi:10.1038/s41467-026-77943-w)
Supplement: Supplementary file 3 [file 41467_2026_77943_MOESM3_ESM.pdf]

Structure of Blm10:13S proteasome intermediate reveals parallel assembly pathways for the proteasome core particle

Mandeep Kaur[#,1], Xiang Chen[#,2], Stella Y. Lee[1], Tyler M. Weaver[1,3], Bret D. Freudenthal[1], Kylie J. Walters[*,2], and Jeroen Roelofs[*,1]

[1]Department of Biochemistry and Molecular Biology, University of Kansas Medical Center, Kansas City, 3901 Rainbow Blvd., HLSIC 1077, Kansas, USA

[2]Protein Processing Section, Center for Structural Biology, Center for Cancer Research, National Cancer Institute, National Institutes of Health, Frederick, MD, USA.

[3] Current address: Department of Biochemistry and Molecular Genetics, University of Virginia School of Medicine, Charlottesville, Virginia 22908, USA

[#]These authors contributed equally

[*]correspondence: kylie.walters@nih.gov or jroelofs@kumc.edu

Running title: Parallel Assembly Pathways for Proteasome Core Particle

Source data: uncropped images main figures.

Source Data – uncropped images immunoblotting

Red dashed boxes provides indication of cropping used in manuscript

Figure 5 pulldown

Blotting sequence: Anti-Pba1/2 followed by anti-Blm10 (no stripping in between)

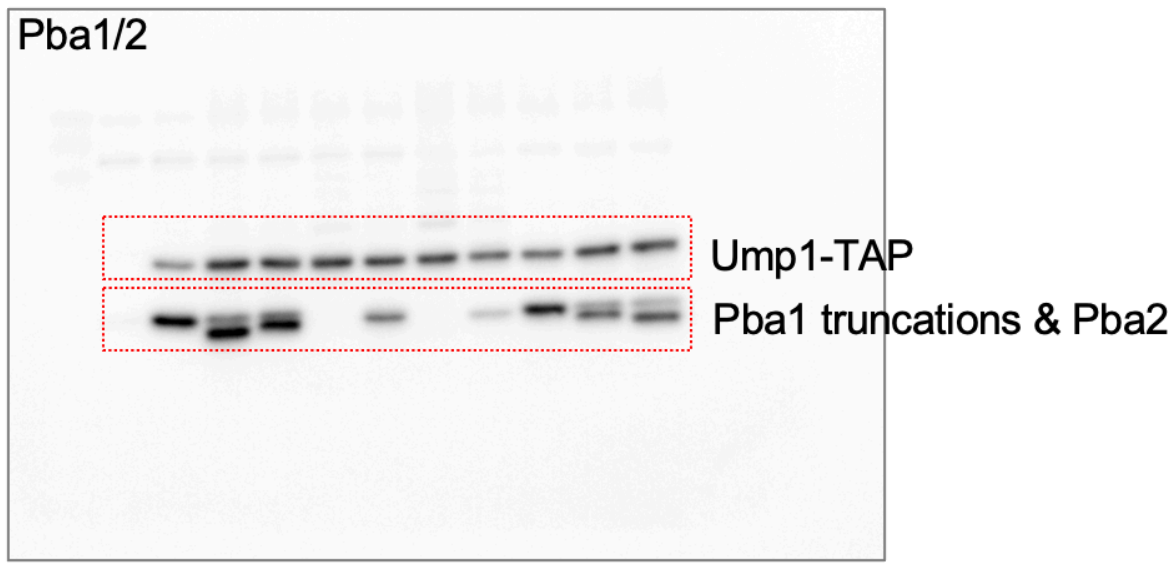

Note: Secondary antibody (rabbit, poly) binds to proA-tag on Ump1

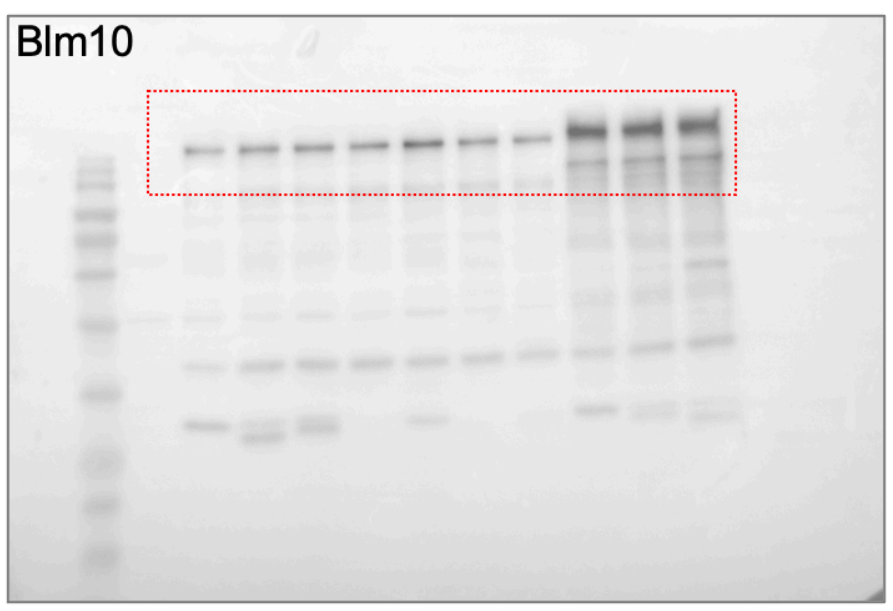

Figure 5 Input

Blotting sequence: Anti-Pba1/2 followed by anti-Blm10 (no stripping in between)

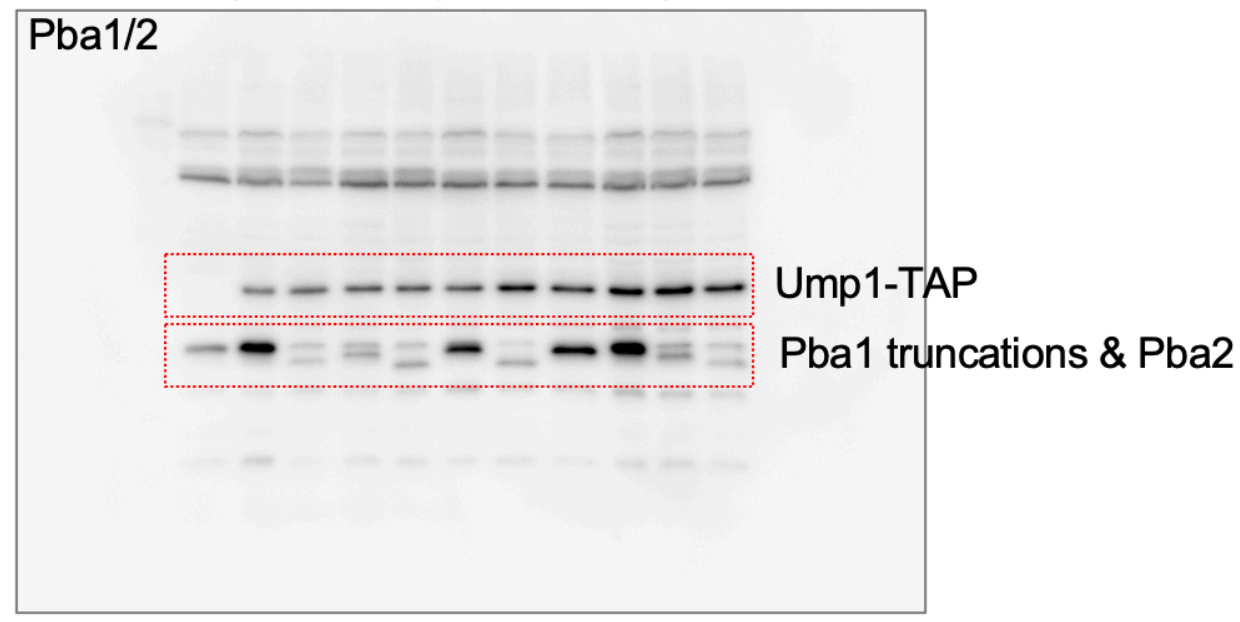

Note: Secondary antibody (rabbit, poly) binds to proA-tag on Ump1

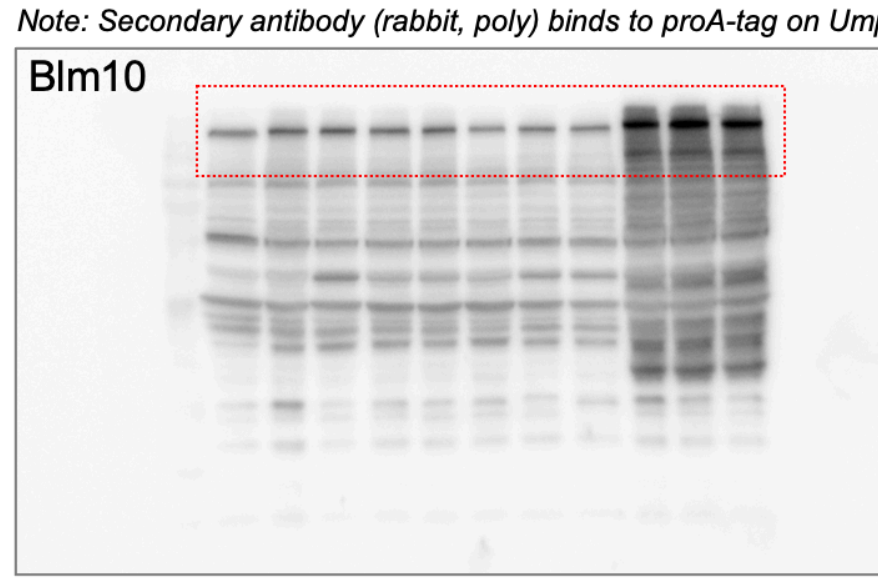

Note: Anti-Blm10 has high background, partly from degradation product and part nonspecific, as confirmed previously using *blm10*Δ cells

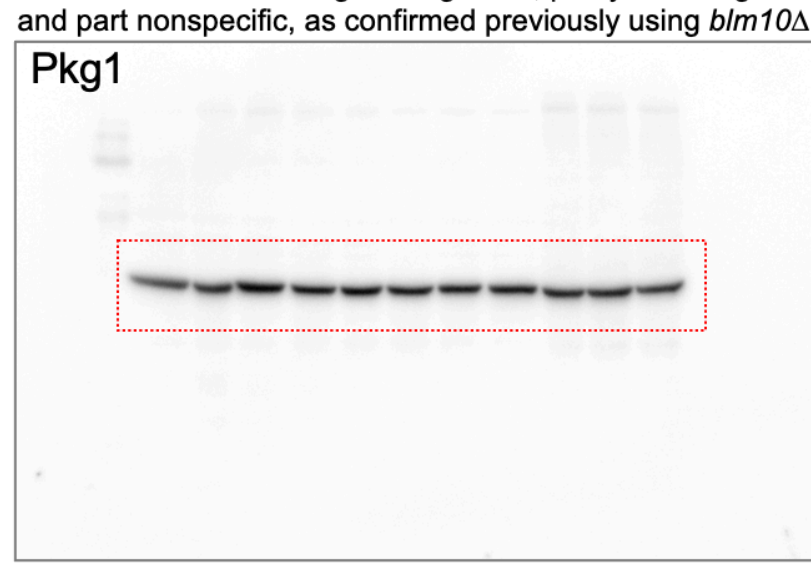

Figure 6 pulldown

Blotting: membrane was cut in two parts;
top was blotted for Blm10, bottom for Pba1/2

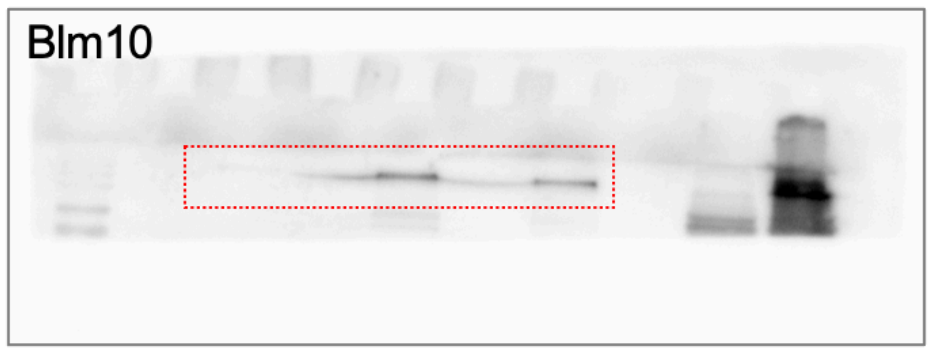

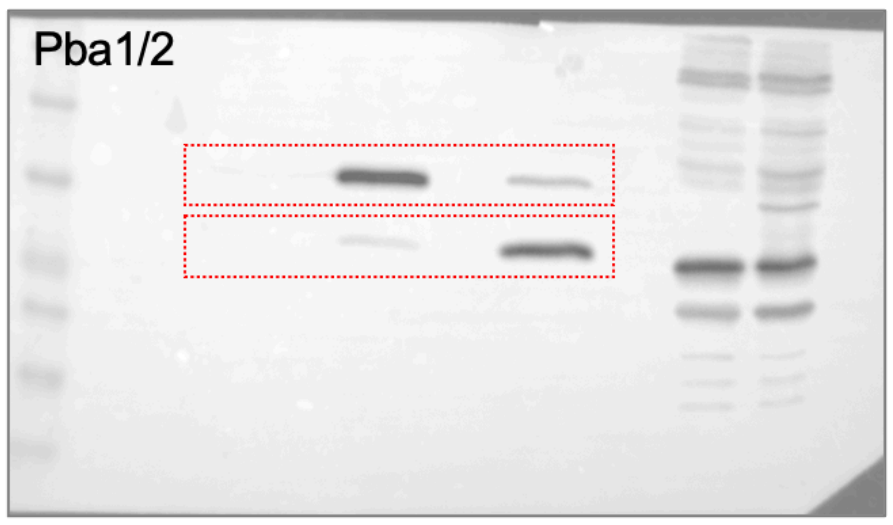

Ump1-TAP / Pba4-TAP

Pba1/2

Note: Secondary antibody (rabbit, poly) binds to proA-tag on Ump1 and Pba4

Figure 6 Input

Blotting: membrane was cut in two parts;
top was blotted for Blm10, bottom for Pba1/2

Blm10

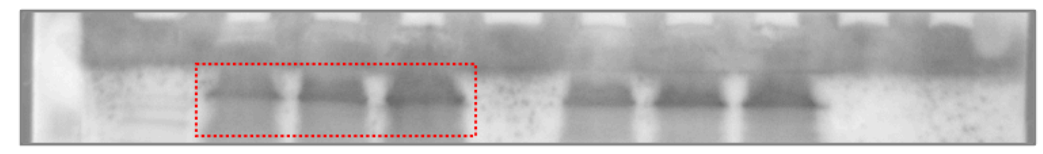

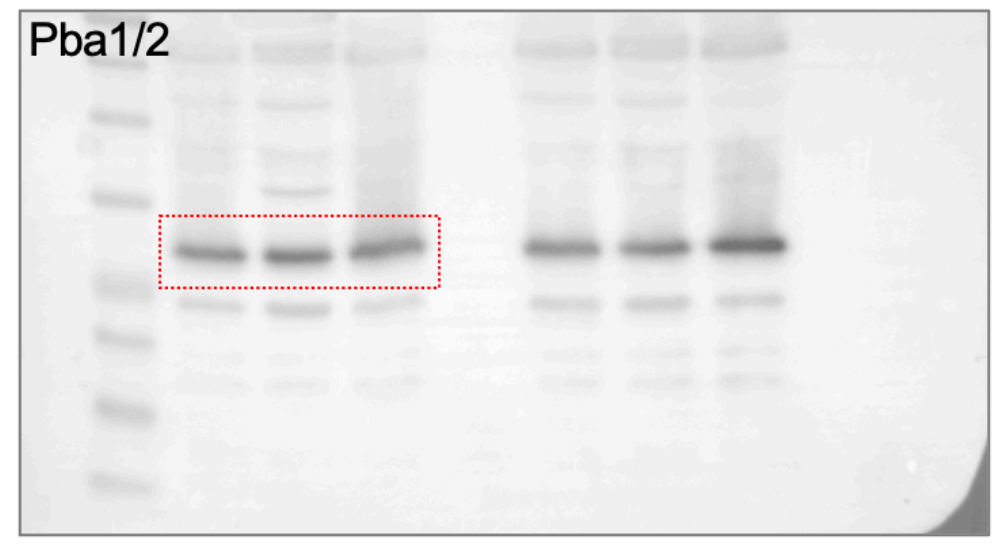

Anti-CBP (part Tap-tag)

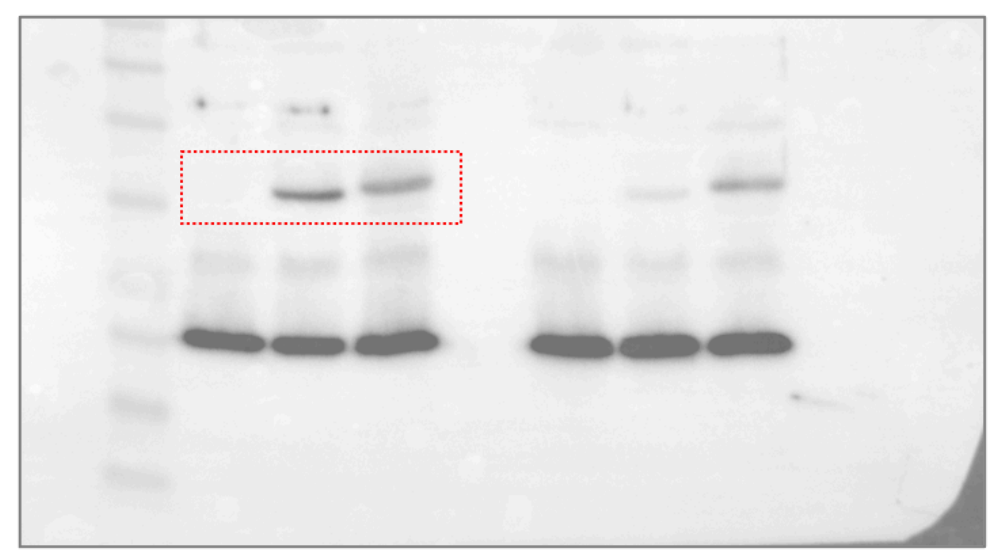

Note: CBP antibody was used to improve sensitivity in total lysate
