# Supplementary material for: Structure of Blm10:13S proteasome intermediate reveals parallel assembly pathways for the proteasome core particle
Source: Nat Commun. 2026 Sep 21;17:10993. doi: 10.1038/s41467-026-77943-w (PMC13683372; doi:10.1038/s41467-026-77943-w)
Supplement: Supplementary file 4 [file 41467_2026_77943_MOESM4_ESM.pdf]

nature portfolio

Peer Review File

Structure of Blm10:13S proteasome intermediate reveals parallel assembly pathways for the proteasome core particle

Corresponding Author: Professor Jeroen Roelofs

This file contains all reviewer reports in order by version, followed by all author rebuttals in order by version.
Editorial Note: Parts of this Peer Review File have been redacted as indicated to remove third party material where no permission to publish were obtained.

Version 0:

Reviewer comments:

Reviewer #1

(Remarks to the Author)
In the paper "Structure of Blm10:13S proteasome intermediate reveals parallel
assembly pathways for the proteasome core particle" the authors describe cryo-EM structures and some biochemical data indicating the existence of alternative pathways of the proteasome maturation leading to the formation of a Bllm10-CP-Blm10 complex independently from the presence of the assembly factor Pba1. We find the finding original and important, view the involvement of the Bllm10-CP-Blm10 complex in very specific degradation pathways of e.g.peptides (important for immune response) or intrinsically disordered proteins. We think that the paper is worth publication but we have some minor doubts that we ask the authors to address.

The strategy chosen (using a delta-Pba1 variant and pull down experiments on Ump1 allowed the authors to obtain good-quality structures of intermediate complexes most likely related to the late stages of the proteasome assembly. However, all throughout the reading of the paper one question lingers:

1. are the beta subunits propeptides visible? The authors should comment on it and show densities if the peptides are visible as pro-peptide presence (together with Ump1 presence) is the first line of evidence, that we are in fact observing assembly intermediates. We understand that it is possible that the pro-peptide regions are flexible and not visible. It is also possible that they get cancelled out by the EMReady post processing process, and it is therefore important that the authors also show densities from the unsharpen maps when necessary.
The maps made available were all sharpened with EMReady and they were not aligned to one another or not even to their respective pdb models. This makes the analysis quite difficult. Please make sure you submit (also) unsharpened maps and aligned models.
Where, in the literature, there is evidence of Pba1 - pro-peptide interaction? (Citation needed in the text, present in the introduction).

Additional questions and suggestions that we have are:

2. It would be ideal to have a Supplementary figure showing comparisons with the existing Blm10(PA200) maps (references 33-35 in the text).
3. When reporting relative amounts of the different states (basically based on how many particles went to each state) it would be important to include also the number of excluded particles (from the 2D cleaned dataset). This is important to understand how much of the data is still unexplained. Do the authors have any map (not shown dataset) without Ump1 and with more b subunits? Again in this case it would be ince to see if these b subunits have the pro-peptide and of Ump1 has then been already digested or was pulled out too strongly.

The paper is mainly dominated by structural information but some little extra biochemistry could make it better and more sound. We would like to ask the authors to:

4. Add a mass spec step after pull down to confirm the identity of everything in the sample.
5. Run native gels followed by WB to confirm the existence of the assemblies. Native could also be coupled with in gel mass spec.
6. The native gels and mass spec and /or WB should be performed from both Ump1 pull downs from delta Pba1 and WT

strains. Such experiments will remove some unclarity from the study. Fore example: In figure 5e, the unchanged level of Blm10 on the assemblies, even in cases of totally abrogated Pba binding (lanes 5 and 7), suggests that they form completely different complexes, confirming the proposed "Parallel pathways of CP assembly." This could be better highlighted in the text. Why is Rpt1 seen in untagged pulldown? By adding a line for a sample from delta Pba1 but with over expression of Blm10, should look like the WT. Such experiment could be performed adn added as extra lines to Fig.5e. In figure 6e Without Pba1, the assembly of the proteasome seems less efficient, while the deletion of Blm10 appears to have almost no effect. Surprisingly, the double deletion also shows a signal similar to the WT. Can the authors comment? How can authors confirm that the band labelled as Blm10-Cp is really this and not a different assembly intermediate?

Using the native gels of delta pba1 or WT pull downs (on Ump1), followed by mass spec or WB could help to confirm the structural data and understand the overall picture.

Other points:
There should be some attention in the interpretation of flexible small regions (e.g. text line 182-184) discussion should be made on unsharpened map. Is the gate openings in the maturating complexes really important? Because at this point the substrate can enter from the b subunits side.

Nice the results/analysis about the Blm10 gates. Regarding this: the open and closed state of CP/Blm10 are related to structured peptidase pockets?

Overall, we think that the paper is reach in information and the structural analysis is properly performed. We would however require that the authors show and/or discuss densities of the pro-peptides, show the density when indicating very specific details /such as the H-bonds networks (Figures 2c and 2f) and that try to confirm the structural data with the usage of native gels and associated WB or mass spec.

Reviewer #2

(Remarks to the Author)
The manuscript under review describes the structures of 5 subassemblies of the yeast proteasome that likely represent intermediates on the assembly pathway. The manuscript pays particular attention to complexes that contain Blm10, which can serve as an alternative proteasome cap or as an assembly factor. The complexes are obtained by pulldowns from yeast strains in which the assembly chaperone Ump1 contains an affinity tag, and proteasome assembly is attenuated by the deletion of the assembly chaperone Pba1.
Structures of assembly intermediates containing Blm10 had not previously been described and the manuscript describes some interesting features of these structures. The structures also reveal the structural basis that prevents the simultaneous presence of Blm10 and the assembly chaperone Pba1/2.

In addition, the manuscript describes a series of pull-down experiments in which the composition of assembly intermediates is investigated by Western blotting. In particular, the manuscript finds that Blm10 can be pulled down with assembly chaperones that are involved in early (Pba4) and in late (Ump1) steps of the assembly pathway; it finds the same for Pba1/2. From these observations, and the fact that Pba1 and Blm10 cannot bind to the same assembly intermediates simultaneously, the manuscript concludes that Blm10 and Pba1/2 must act in parallel assembly pathways. This conclusion is certainly plausible. However, conclusions about pathways without some kinetic information are, by necessity, speculative. Could the different assemblies represent off-pathway intermediates?

Reviewer #3

(Remarks to the Author)
I co-reviewed this manuscript with one of the reviewers who provided the listed reports. This is part of the Nature Communications initiative to facilitate training in peer review and to provide appropriate recognition for Early Career Researchers who co-review manuscripts.

Reviewer #4

(Remarks to the Author)
Kaur et al. used a TAP-tagged Ump1 proteasome assembly chaperone to purify assembly intermediates from baker's yeast and report five cryoEM structures of partially assembled 20S proteasome core particles (CPs). Two of the complexes include the proteasome regulatory factor Blm10 bound to the alpha-subunit ring of intermediates, one which apparently lost the Ump1 factor used in the affinity purification. By comparing their structures among themselves and to two previous CP structures (Pba1/2-13S and Blm10-CP), they suggest potential functions for various observed molecular interactions and conformational states, such as gate opening of the alpha ring and size variation of the aperture in the dome-shaped Blm10 protein structure.

The cryoEM structures, gathered on a state-of-the-art microscope, appear to be of high quality and have high overall resolutions. Most of the inferences about function are speculative, which I am fine with, but the main speculation, which is part of the title, is that Blm10 is part of a novel, parallel pathway of CP assembly. The straw man in this is that the prevailing model in the field holds that the Pba1/2 assembly factor (acting early) and Blm10 (acting late) function sequentially in CP

assembly. There really is no agreement in the field on this idea, and the authors merely cite two review articles that speculate that this might be the case. In fact, the real question is whether Blm10 has any role at all in CP assembly. The authors themselves note in their Introduction that "[i]t is unclear what role Blm10 plays when bound to assembly intermediates…." Unfortunately, this statement is still true following my reading of the results presented here. If the alpha-ring of various intermediates is not bound by Pba1/2, which would clash sterically with Blm10 binding, then the exposed ring surface could simply associate with available Blm10, which has an immense binding surface for binding to the alpha ring, as well as the HbYX motif at its C-terminus. Blm10 might still function entirely as a regulator of mature CP proteolytic activity, as originally proposed by Schmidt and colleagues.

My opinion is that these new structures are fine in and of themselves but add only incrementally to our understanding of CP assembly given the large number of papers on CP assembly intermediates published in the past few years. Neither the data nor the proposed model of parallel Pba1/2 and Blm10 dependent CP assembly pathways resolves whether Blm10 truly has a role in assembly. As such, I would recommend the current manuscript for a more specialist structure journal.

Other comments:

Line 168. While I believe that CP intermediates involving incomplete alpha rings are likely to be on-pathway for CP assembly or at least resolvable into competent assembly intermediates, I think the concluding sentence here needs to be tempered: "Thus, at least in this pba1D background, assembly does not always proceed by formation of the a-ring first." I would add "probably" before "does not".

Line 188: The "closed state" still has a sizeable opening of the Blm10 aperture. Would an unfolded polypeptide chain still be able to thread through it?

Line 195: "In all, these data suggests that the Blm10 dome opening can change from a closed state to an open state, enabling potential allosteric regulation of Blm10-dependent protein degradation." There are no data here that directly address effects on Blm10-dependent degradation.

Figure 6b: Looks to me like you pull down the 'other' chaperone as well, e.g., Blm10-TAP pulls down some Pba4 and vice versa. Maybe Blm10 and Pba4 binding is not fully mutually exclusive?

Version 1:

Reviewer comments:

Reviewer #1

(Remarks to the Author)
We really appreciate all the work the authors put onto the manuscripts and new experiments to address our questions and requests.
The paper, which was already good, has further improved.

Reviewer #3

(Remarks to the Author)
I co-reviewed this manuscript with one of the reviewers who provided the listed reports. This is part of the Nature Communications initiative to facilitate training in peer review and to provide appropriate recognition for Early Career Researchers who co-review manuscripts.

Reviewer #4

(Remarks to the Author)
I like the rewriting of the abstract and introduction, and some of the new genetic data are revealing. However, while the structures of Blm10-13S, etc. in the manuscript are fine in and of themselves, I still am not convinced of their physiological function in CP (or Blm10:CP) assembly.

Specific comments:
Fig. 1a. Anti-Blm10 blot should have individual species labeled. It is hard to tell which bands are what.

Line 79. The CTE of beta7 is part of the mature protein. It is not a propeptide.

Line 125. I don't see evidence that the Blm10-CP-RP complexes are proteolytically active, as stated. These particles are not obviously resolved from RP-CP complexes in the substrate overlay assays in these native gels. Consistent with this point, the authors do not mark this active Blm10-CP-RP complex in extended data Fig. 1a. Also, these blots should have included an anti-RP antibody to show its inclusion in the larger species (although this is highly likely!)

Line 185: "There is no structural indication that 5 3 Ump1 and 5 4 Ump1 represent dead-end products because all subunits are correctly ordered in these intermediates." But I think there is no functional evidence that they are on-pathway

intermediates either. True?

Line 244: However, despite this open gate conformation, the complex is unlikely to be catalytically active, as the Blm10 dome is closed" And because the catalytic sites are physically absent!

Line 324. "Thus, both Blm10 and Pba1 are present in early as well as late stages of CP assembly, which is consistent with parallel assembly pathways." Potentially supporting this, a previous report had also seen both Blm10 and Pba1 in 13S-like particles (doi: 10.1038/sj.emboj.7601681.)

Line 338. What exactly is this beta7-CTE mutant? Should clarify.

Line 341. What kind of terminator? Stop codon? Transcriptional terminator?

Line 343. "Deletion of Pba1 in this background did not further sensitize these cells" Another interpretation is that Blm10 simply does not have a significant effect on CP assembly. Alternatively, the fact that the double pba1 blm10 deletion does not exacerbate the growth defects of the singles could also still argue that they work in the same pathway.

Line 353. "revealing mutually exclusive binding of Blm10 and Pba1/Pba2 to the 13S" This was pretty much guaranteed to be true given that Blm10 occludes virtually the entire alpha-ring end surface.

Line 368. "…Blm10 or RP might substitute for Pba1/Pba2 function. However, the lack of a synergy phenotype between BLM10 and PBA1 in the rpn4delta or beta7CTE backgrounds, suggest this is not an important function of the Blm10:13S assembly pathway." As above, another interpretation is that Blm10 has a function distinct from CP assembly, and the small fraction of Blm10-13S complexes observed might be physiologically insignificant or involved in some kind of quality control or proteasome nuclear import as others have proposed.

In their rebuttal letter, the authors state, based on this lack of genetic interaction (which was not what they had originally predicted), "Therefore, we favor the model whereby Blm10 association early in CP assembly ensures the formation of Blm10:CP complexes." This is possible but assumes slow dissociation of Blm10 from assembly intermediates and Blm10:CP complexes. Is there evidence for this? If Blm10:CP complexes are purified, do they limit RP-CP-RP formation when challenged with RP at relative concentrations that are close to physiological?

Version 2:

Reviewer comments:

Reviewer #4

(Remarks to the Author)
The authors have made reasonable arguments addressing all of the points in my last review, and I am happy to see this move forward for publication.

Open Access This Peer Review File is licensed under a Creative Commons Attribution 4.0 International License, which permits use, sharing, adaptation, distribution and reproduction in any medium or format, as long as you give appropriate credit to the original author(s) and the source, provide a link to the Creative Commons license, and indicate if changes were made.
In cases where reviewers are anonymous, credit should be given to 'Anonymous Referee' and the source.
The images or other third party material in this Peer Review File are included in the article's Creative Commons license, unless indicated otherwise in a credit line to the material. If material is not included in the article's Creative Commons license and your intended use is not permitted by statutory regulation or exceeds the permitted use, you will need to obtain permission directly from the copyright holder.
To view a copy of this license, visit https://creativecommons.org/licenses/by/4.0/

We appreciate the feedback from all reviewers and the extra time provided to submit this revision. We hope that the reviewers and editorial team agree that the additional data together with a better explained narrative and logic has addressed all concerns, and that the manuscript is now suited for publication in Nature Communications.

Point to point rebuttal on reviewer comments

Reviewer #1 (Remarks to the Author):

In the paper "Structure of Blm10:13S proteasome intermediate reveals parallel assembly pathways for the proteasome core particle" the authors describe cryo-EM structures and some biochemical data indicating the existence of alternative pathways of the proteasome maturation leading to the formation of a Bllm10-CP-Blm10 complex independently from the presence of the assembly factor Pba1. We find the finding original and important, view the involvement of the Bllm10-CP-Blm10 complex in very specific degradation pathways of e.g.peptides (important for immune response) or intrinsically disordered proteins. We think that the paper is worth publication but we have some minor doubts that we ask the authors to address.

We appreciate the reviewer's recognition of our findings as "*original and important"* and the assessment that the manuscript is worthy of publication in Nature Communications.

The strategy chosen (using a delta-Pba1 variant and pull down experiments on Ump1 allowed the authors to obtain good-quality structures of intermediate complexes most likely related to the late stages of the proteasome assembly. However, all throughout the reading of the paper one question lingers:

1. are the beta subunits propeptides visible? The authors should comment on it and show densities if the peptides are visible as pro-peptide presence (together with Ump1 presence) is the first line of evidence, that we are in fact observing assembly intermediates. We understand that it is possible that the pro-peptide regions are flexible and not visible. It is also possible that they get cancelled out by the EMReady post processing process, and it is therefore important that the authors also show densities from the unsharpen maps when necessary. The maps made available were all sharpened with EMReady and they were not aligned to one another or not even to their respective pdb models. This makes the analysis quite difficult.

We agree with the reviewers that the presence of β subunit propeptides is an important indication of an assembly complex. Of the seven β subunits, five contain propeptides; in the Blm10:13S structure, only the β2 propeptide is present. We apologize for not stating this clearly in the original manuscript, but we did observe the propeptide of β2 in four of the five pre-assembly intermediate complexes. We now report this observation in the Figure 1 overview of the structures.

Also, for the Blm10:13S structure, Extended Data Fig. 2f,g and 6 shows residues A2-T29 of β2 in green, corresponding to the propeptide region preceding the active site residue T30. In the revised manuscript, we describe this feature explicitly in the Results section. We note that the initiator methionine is not observed and likely removed by methionine aminopeptidase during translation.

Please make sure you submit (also) unsharpened maps and aligned models.

Thank you for this helpful reminder. We have updated the relevant figures to include the unsharpened maps. In the revised manuscript, electron densities from the unsharpened maps have been added to Figures 2f, 4b and 5a, as well as Extended Data Figures 2f, 2g, 4a, 4b, and 5a.
In the revised submission, the unsharpened maps, EMReady-sharpened maps, and corresponding PDB structures have been aligned with validation reports included in the attached files. The associated maps and coordinates files have also been updated in EMDB and PDB.

Where, in the literature, there is evidence of Pba1 - pro-peptide interaction? (Citation needed in the text, present in the introduction).

We describe this later in the manuscript with reference, but the reviewer is correct that the last sentence of the introduction implies this as well. We now added the reference to support this statement (Schnell et al, 2021 Nat Struct Mol Biol, PMID: 33846632).

Additional questions and suggestions that we have are:

2. It would be ideal to have a Supplementary figure showing comparisons with the existing Blm10(PA200) maps (references 33-35 in the text).

We apologize, we have several comparisons between Blm10:13S and Blm10:CP and are not sure what specific comparison the reviewer is looking for.

3. When reporting relative amounts of the different states (basically based on how many particles went to each state) it would be important to include also the number of excluded particles (from the 2D cleaned dataset). This is important to understand how much of the data is still unexplained. Do the authors have any map (not shown dataset) without Ump1 and with more β subunits? Again in this case it would be nice to see if these β subunits have the pro-peptide and of Ump1 has then been already digested or was pulled out too strongly.

Thank you for pointing this out. The relative abundance of the two Blm10-containing complexes has been corrected in the revised manuscript to 24% of the total particles, rather than 44% as previously stated. In addition, Extended Data Fig.1 has been updated to include further classification of the excluded particles (1,833,1412 particles). All five excluded classes appear to represent small or potentially denatured particles.

An affinity tag on Ump1 was used for all purifications. Autocatalytic activation of the active sites occurs only at the final stage of CP assembly, when Ump1 becomes enclosed within the CP. At this stage, Ump1 is enclosed, gets degraded and the purification tag is inaccessible for purification. To our knowledge, neither our experiments nor the literature provide evidence that propeptide are cleaved or that proteolytic activity occurs prior to complex CP assembly. The only structure lacking Ump1 is the Blm10:α-ring complex, which most likely reflects Ump1 dissociation during sample preparation, as Ump1 carried the affinity tag. Although the β2 propeptide is not resolved in this structure, we consider it more likely that this reflects its reduced structural organization in the absence of Ump1 and additional β subunits, rather than premature propetide cleavage. We have clarified this point in the revised manuscript. In Extended Data Fig. 1, the class 3 structures and

excluded particles likely represent smaller assembly intermediates or fragments generated during sample or cryo-grid preparation, or potentially denatured particles. These species lacked sufficient structural homogeneity to permit further analysis or to draw definitive conclusions regarding propeptide presence or absence.

The paper is mainly dominated by structural information but some little extra biochemistry could make it better and more sound. We would like to ask the authors to:

4. Add a mass spec step after pull down to confirm the identity of everything in the sample.

To determine these structures, we started with 10 L of yeast culture and ended up with about 20 microliter of purified sample, all of which was used for grid preparation. As the entire preparation was consumed for cryo-EM analysis, it was not possible to perform mass spectrometry on the identical sample; a separated purification would have to suffice. However, this has basically already been done and published by us in the past: we have conducted similar purifications with these strains in the past and analyzed them by 2D-PAGE and mass spectrometry, confirming that the major proteasome components correspond to the proteasome subunits identified in our structures, including the β2 propeptide and Blm10 (Wani et al, 2016 Nat.comm, PMID: 25812915). Furthermore, the cryo-EM density maps do not contain any unexplained or unassigned density, further supporting that the resolved structures are consistent with the identity of the components. We have clarified this point in the revised manuscript.

5. Run native gels followed by WB to confirm the existence of the assemblies. Native could also be coupled with in gel mass spec.

We hope we correctly interpreted the reviewer's concern here, namely whether Blm10-bound assembly intermediates complexes are observed in wild-type cells and not an artifact resulting from purification or the use of a particular mutant yeast strain. To address this, we ran a native gel of total lysate of wild-type, blm10Δ, and pba1Δ cells (new Fig. 1a, see also below). We also ran purified Blm10:13S on native gel (new Extended Data Fig. 1a, lane 4, see also below) and conducted a reconstitution experiment. Note assembly intermediates lack LLVY-amc activity and migrate faster than the CP (lowest band with LLVY-amc activity). For the total lysate in panel a, the pba1Δ strain clearly shows a Blm10 positive species that migrates slightly slower than CP. This is consistent with previous publications showing Blm10 binds to assembly intermediates in mutant backgrounds (Li et al, 2007 EMBO j. PMID: 17431397). While this band is enriched in pba1 deletion, we, importantly, here show it is also present in wild-type cells. Furthermore, the enrichment in Blm10 containing species in *pba1Δ* is similar for both assembly intermediates as well as the complexes with mature CP (as indicated by LLVY-amc activity), indicating the assembly complex is not a dead-end product that accumulates selectively, but further matures into functional complexes (Blm10-CP, Blm10-CP-Blm10, and Blm10-CP-RP). To further explore this functionality, we also conducted some reconstitution experiments, where the purified Blm10:13S complex was mixed with lysate of wild-type or *blm10Δ* cells. The Blm10 immunoblot of lane 4 shows only assembly intermediates, upon mixing this purified complex with lysate of *blm10Δ* (lane 3), Blm10-CP-RP complexes appear. This finding shows that the Blm10 from the purified assembly intermediates can form proteolytically active CP complexes.

We appreciate the reviewer asking about the existence of the assemblies and include these new data in the results section of the revised manuscript.

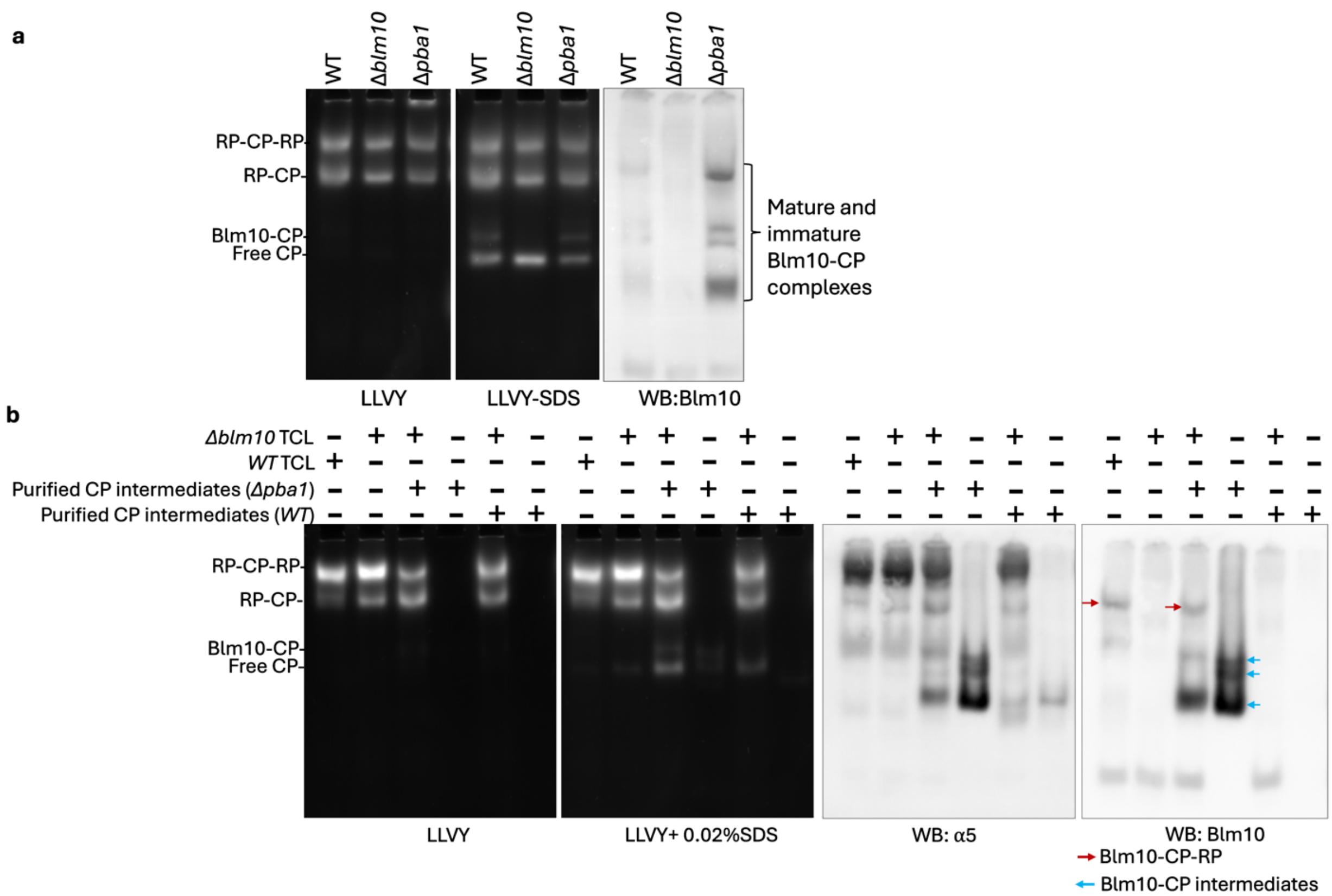

New data as Fig. 1a (top) and Extended Data Fig. 1A (bottom): Blm10:13S are endogenous complexes that mature. a) Blm10:13S complexes are present in wild type cells and enriched upon deletion of PBA1. Indicated yeast strains were grown for 24 hours in YPD and lysed. Lysates were separated by native PAGE and visualized using in-gel LLVY-amc peptidase activity assay in the presence and absence of 0.02% SDS. Next, the gel was transferred to PVDF membrane and immunoblotted for Blm10 (right most panel). Four Blm10 positive bands are fomr the top Blm10-CP-RP, Blm10-CP-Blm10, Blm10-CP, and Blm10 bound to assembly intermediate (no proteolytic activity). **b)** Reconstitution of purified Blm10 bound CP intermediates with total cell lysates leads to formation of mature Blm10-CP-RP complexes**.** Purified CP intermediates from WT or Δ*pba1* strains were incubated with cleared total of wild type or *blm10*Δ strains for 30 mins at 30 °C. Next, samples were separated on native PAGE, followed by in-gel peptidase activity assay. The gels were transferred onto PVDF membrane and blotted for α5 subunit and Blm10. The lane4 in right two panels show the purified assembly intermediate from *pba1*Δ (similar to prep used for cryo-EM structure determination). In lane 3 this was mixed with lysate from *blm10*Δ cells and the formation of Blm10 positive slower migrating bands indicates the formation of Blm10-CP and Blm10-CP-RP species (marked by red arrow). These results suggest that the Blm10 associated assembly intermediates (Blm10:13S) is functional and can upon reconstitution with TCL from *blm10*Δ strain can form mature Blm10 bound CP complexes.

6. The native gels and mass spec and /or WB should be performed from both Ump1 pull downs from delta Pba1 and WT strains. Such experiments will remove some unclarity from the study. For example: In figure 5e, the unchanged level of Blm10 on the assemblies, even in cases of totally abrogated Pba binding (lanes 5 and 7), suggests that they form completely different complexes, confirming the proposed "Parallel pathways of CP assembly." This could be better highlighted in the text. By adding a line for a sample from delta Pba1 but with over expression of Blm10, should look like the WT. Such experiment could be performed and added as extra lines to Fig.5e.

We appreciate the reviewer for these valuable suggestions. The immunoblots of total lysate shown above and added as supplementary figure to the paper, we would argue, best illustrate the point we think the reviewer makes here. We'd like to point out that Blm10 is substoichiometric and that we are not aware of any paper showing an excess of free Blm10 in cells, apart from our JBC publication where we strongly overexpressed Blm10 (Burris et al, 2021 JBC, PMID: 33639167). The increased Blm10 in the pba1 deletion might reflect a stabilization of Blm10 or increased expression. Furthermore, overexpression of Blm10 leads to increase in Blm10 bound to immature CP complexes, although there seems to be a limit to this (Burris et al, 2021 JBC, PMID: 33639167 and Fig 5).

We thank you for encouraging us to incorporate the immunoblots of total lysates with wild-type and *pba1*Δ and appreciate the reviewer pointing out how such data supports our argument. We have incorporated this argument, which is a bit different from the one laid out by the reviewer, in our revised manuscript.

Why is Rpt1 seen in untagged pulldown?

The proteasome is a very abundant complex in cells and the assembly complexes are substantially less abundant, which increase the chance of some proteasome subunit background. Our focus in this assay has been mainly on Blm10, Ump1, and Pba1/2. Rpt1 signal is indeed seen in the pulldown with untagged strains as the reviewer points out. We initially included Rpt1 as we see some value in that information but agree that the substantial Rpt1 background makes interpretation of Rpt1 ambiguous and therefore, we have excluded it in the updated figure, since it has no direct bearing on the conclusions.

In figure 6e Without Pba1, the assembly of the proteasome seems less efficient, while the deletion of Blm10 appears to have almost no effect. Surprisingly, the double deletion also shows a signal similar to the WT. Can the authors comment? How can authors confirm that the band labelled as Blm10-Cp is really this and not a different assembly intermediate?

The reviewer raises an excellent point that likely reflects some of the complexity *in vivo* resulting from feedback mechanisms and compensation. To fully sort this out would go beyond the current scope of this manuscript, but there are some reasonable arguments and putative explanations we can provide. These strains are not deleted for RPN4, the transcription factor that can dramatically upregulate proteasome subunits. For example, the absence of proteasome chaperones can be compensated for by increased expression of proteasome subunits (using the transcription factor Rpn4), as is e.g. evident from the severe phenotypes that have been reported for *rpn4*Δ *pba1*Δ or *rpn4*Δ *pba2*Δ (figure below from Le Tallec et al. Mol. Cell 2007 PMID:

17707236: POC1 = Pba1, and our new supplementary data). Furthermore, in the PBA1 deletion strain some of the CP and immature CP is bound to the Blm10 (see new supplementary figure), resulting in the Blm10:CP complex and reduced availability for RP to bind CP. With the deletion of both PBA1 and BLM10 all CP that manages to form upon overexpression with chaperones now binds RP, presumably causing the increase in RP_2-CP and RP-CP compared to PBA1 delete. Our updated manuscript has additional phenotype studies to determine if the Blm10 and Pba1/Pba2 dependent assembly pathways are redundant or have separate functions. Counter to our initial expectations, our data suggest these pathways are not redundant. We feel Pba1/Pab2 is very important for CP formation, while the Blm10:13S pathway might be mainly to secure formation of Blm10:CP complexes.

[REDACTED]

The Blm10-CP band is Blm10 bound to mature CP as it had activity on the gel. This band is absent in a strain deleted for Blm10, and more abundant in strains overexpressing Blm10. In all, the assignment is consistent with all our previous work (see e.g. Burris et al, 2021 JBC, PMID: 33639167) and consistent with data from several other labs. Please note this complex is not an assembly intermediate, as it has proteolytic activity. While we feel confident about these assignments, the only conclusion we draw from this figure is to illustrate that in the absence of PBA1 and BLM10, CP can still from, indicating assembly is possible without these two genes. Admittedly, this idea is not novel and has been shown before, but we fell it prudent to show here as well.

Using the native gels of delta pba1 or WT pull downs (on Ump1), followed by mass spec or WB could help to confirm the structural data and understand the overall picture.

Please see our comments above for points 5 & 6.

Other points:
There should be some attention in the interpretation of flexible small regions (e.g. text line 182-184) discussion should be made on unsharpened map. Is the gate openings in the maturating complexes really important? Because at this point the substrate can enter from the b subunits side.

Thank you for this helpful point. In the revised Figures 4b and 5a, as well as Extended Data Figures 4a, 4b, and 5a, unsharpened maps have been included to facilitate interpretation of the flexible small regions.

We agree with the reviewer that, at this stage substrate entry and exposure to the active sites is unlikely to be relevant. That said, there might be other reasons it could be relevant, as in general, this gate is tightly regulated. For example, if a polypeptide were to enter the Blm10 dome via the CP gate, it could potentially restrict the conformational changes that would have to happen for transitions. Alternatively, interactions within the Blm10 dome might transiently trap polypeptides in a region important for gate opening or propeptide-Ump1 interactions, thereby influencing assembly dynamics. Interaction on the opposite side of CP are less likely to have such effects, as there is no dome to trap substrates and that space is initially occupied by assembly chaperones (Pba3/4 initially, followed by Ump1). Overall, we were intrigued by this observation and find it noteworthy for many labs that report on CP gate opening with the various CP activators or might have data in the future for which it becomes relevant.

Nice the results/analysis about the Blm10 gates. Regarding this: the open and closed state of CP/Blm10 are related to structured peptidase pockets?

Thank you for this comment. We are not entirely certain what is being asked regarding the peptidase pockets. There are pockets at the α subunit interfaces into which the C-termini of regulators dock. One such pocket (the α5/ α6 interface) is occupied by the Blm10 HbYX motif; however, this site does not possess peptidase activity. With respect to the catalytic β subunit active sites (with peptidase activity): in the Blm10:CP and Blm10:13S structures, the α-ring gate is open and the catalytic β subunits (β1, β2, and β5) are present, although no substrate is engaged. In the Blm10: α-ring structure the α-ring gate is also open and only β2 is present.

Overall, we think that the paper is reach in information and the structural analysis is properly performed. We would however require that the authors show and/or discuss densities of the pro-peptides, show the density when indicating very specific details /such as the H-bonds networks (Figures 2c and 2f) and that try to confirm the structural data with the usage of native gels and associated WB or mass spec.

Thank you for these helpful suggestion which as outlined above we have implemented. We have included unsharpened maps in the revised Figures 4b and 5a, as well as Extended Data Figures 2f, 2g, 4a, 4b, and 5a. We have also discussed the pro-peptides with new Extended Data Fig. 2. In our previous publication, we validated the structural composition of a similarly purified complex by mass spectrometry, which supports the component assignments presented here. We hope that the clarifications and additional data provided above address the concerns raised. We appreciate the reviewer's thoughtful comments, which have helped us improve the manuscript.

Reviewer #2 (Remarks to the Author):

The manuscript under review describes the structures of 5 subassemblies of the yeast proteasome that likely represent intermediates on the assembly pathway. The manuscript pays particular attention to complexes that contain Blm10, which can serve as an alternative proteasome cap or as an assembly factor. The complexes are obtained by pulldowns from yeast strains in which the assembly chaperone Ump1 contains an affinity tag, and proteasome assembly is attenuated by the deletion of the assembly chaperone Pba1. Structures of assembly intermediates containing Blm10 had not previously been described and the manuscript describes some interesting features of these structures. The structures also reveal the structural basis that prevents the simultaneous presence of Blm10 and the assembly chaperone Pba1/2.

We thank the reviewer for this succinct summary of our manuscript and the notion that these likely represent intermediates of the assembly pathway.

In addition, the manuscript describes a series of pull-down experiments in which the composition of assembly intermediates is investigated by Western blotting. In particular, the manuscript finds that Blm10 can be pulled down with assembly chaperones that are involved in early (Pba4) and in late (Ump1) steps of the assembly pathway; it finds the same for Pba1/2. From these observations, and the fact that Pba1 and Blm10 cannot bind to the same assembly intermediates simultaneously, the manuscript concludes that Blm10 and Pba1/2 must act in parallel assembly pathways. This conclusion is certainly plausible. However, conclusions about pathways without some kinetic information are, by necessity, speculative. Could the different assemblies represent off-pathway intermediates?

The reviewer raises a good point and one that is not trivial to address. While the assembly intermediate has been purified and previously been observed in a mutant background, we now added data showing the same intermediate is detectable in wild-type cells as well (see e.g. comments to reviewer 1 and new native gel in the Supplementary Fig. 1). The increase in the mutant background that enabled us to purify the complex is thus less likely to be an artifact. In addition, comparing the different Blm10 complexes in wild-type and mutant strains, we would hypothesize that if the Blm10:13S was a dead-end product, it would be specifically enriched in the mutant strain. This is, however, not what we observed, as the ratio of Blm10 bound to mature CP and Blm10 bound to assembly intermediates remained similar. Thus, the increase in Blm10:13S complexes seems to result in more Blm10 mature CP complexes. Obtaining kinetic data for proteasome assembly is beyond the scope of our study; for example, a study looking at assembly of the RP with pulse chase required exposure of radiolabeled gels for up to two months (Park et al, 2009, Nature, PMID: 19412160).

We appreciate this reviewer's comment as it also broadens our attention to another question, namely, are there structural indications that this complex might be a dead-end product? The striking similarities in subunit position between the Pba1/Pba2:13S and Blm10:13S would make it hard to see why the Blm10:13S would be a dead-end product as the interaction surfaces available for association by other CP subunits are close to identical. Furthermore, considering the tightly controlled CP assembly process, we would argue that it would be more surprising if this was a dead-end product or off-pathway product that the cell allows to be formed. Finally, if Blm10:13S was a dead-end product, it would be unlikely that strong overexpression of Blm10 would yield any level of mature CP, especially in a pba1 deletion background. We have shown

previously that strong overexpression yields high levels of Blm10-CP complexes (Burris et al, 2021 JBC, PMID: 33639167). The other conundrum is that people can readily detect free RP in the cell, but no free Blm10, so if Blm10 would just accidentally dock onto an alpha1 during assembly, why do we detect Blm10:13S complexes in wild type cells (see supplementary fig.1), but no RP:13S complexes (Wani et al, 2016 Nat.comm, PMID: 25812915)? Apparently, Pba1/Pba2 prevents RP binding, but not Blm10 binding and we find Pba1/Pba2 binding to be mutually exclusive to Blm10 binding CP. It is not completely clear why this is the case, but it argues against some random coincidental association and likely involves an ability of Blm10, unlike RP, to associate earlier with (partial) α-ring assemblies. For example, both Pba1/Pba2 and Blm10 might both bind to α5-α6 dimers and stabilize their incorporation, which would be consistent with the observed complexes that lack α5-α6 but have the other alpha subunits and several beta subunits). Alternatively, the action of RP chaperones block RP-CP interactions; this possibility is something we hope to explore more in the future.

Reviewer #3 (Remarks to the Author):

I co-reviewed this manuscript with one of the reviewers who provided the listed reports. This is part of the Nature Communications initiative to facilitate training in peer review and to provide appropriate recognition for Early Career Researchers who co-review manuscripts.

Thank you.

Reviewer #4 (Remarks to the Author):

Kaur et al. used a TAP-tagged Ump1 proteasome assembly chaperone to purify assembly intermediates from baker's yeast and report five cryoEM structures of partially assembled 20S proteasome core particles (CPs). Two of the complexes include the proteasome regulatory factor Blm10 bound to the alpha-subunit ring of intermediates, one which apparently lost the Ump1 factor used in the affinity purification. By comparing their structures among themselves and to two previous CP structures (Pba1/2-13S and Blm10-CP), they suggest potential functions for various observed molecular interactions and conformational states, such as gate opening of the alpha ring and size variation of the aperture in the dome-shaped Blm10 protein structure.

The cryoEM structures, gathered on a state-of-the-art microscope, appear to be of high quality and have high overall resolutions.

Thank you for recognizing the high quality of these structures.

Most of the inferences about function are speculative, which I am fine with, but the main speculation, which is part of the title, is that Blm10 is part of a novel, parallel pathway of CP assembly. The straw man in this is that the prevailing model in the field holds that the Pba1/2 assembly factor (acting early) and Blm10 (acting late) function sequentially in CP assembly. There really is no agreement in the field on this idea, and the authors merely cite two review articles that speculate that this might be the case.

We don't disagree with the reviewer point that there might be no agreement in the field on this idea. The reviewer argues that we "merely cite" two reviews that speculate this might be the case. However, we would like to point out that many reviews on proteasome assembly do not discuss any model regarding the role of Blm10 in assembly or don't mention Blm10 at all (see table below), the few that do present the model with Pba1/2 assembly factor (acting early) and Blm10 (acting late) as we cited. We would argue that objectively anybody familiarizing themselves with the field would conclude that this is the current model based on these recent reviews. Additionally, we would like to point out that the reviews with this model are by two prominent labs that have both published substantial primary literature on proteasome assembly and are recognized as experts on the subject (Vierstra and Murata laboratories). To our knowledge any ongoing debates are not reflected in the literature, and we are not aware of any review articles or primary literature bringing up the concept of parallel versus sequential assembly pathways for CP (please correct us if we are wrong and we would be happy to cite them). In all, we feel our representation is fair and the concept will be original to the broad readership not purview to discussions outside the scientific literature realm. We did amend our text to reflect the literature that does not present any models for the role of Blm10 in assembly as follows: "While many reviews of proteasome assembly

do not specify a functional role for Blm10[31-35], those that do propose a sequential model whereby Blm10 interacts with CP after Pba1/Pba2 dissociates and prior to RP binding[36,37]."

We also, rewrote our abstract which overemphasized that one model and now has more neutral language that we hope the reviewer feels more fairly represents the past and new thinking.

Recent reviews and papers on proteasome CP assembly:

Paper/review	Blm10 text?	Blm10 model in assembly?
PMID: 39211201<br>**2024 Nat Commun – research paper**<br>"Structural basis of human 20S proteasome biogenesis"<br>Zhao Laboratory	Blm10 in assembly not mentioned	Not mentioned
PMID: 39260885<br>**2024 Life Sci Alliance – research paper**<br>"Structural roles of Ump1 and β-subunit propeptides in proteasome biogenesis"<br>Dohlman & Wendler Laboratories	Present structure of native Blm10-CP from mutant strain and mentioned others have seen Blm10 bound to immature complexes	No model on Blm10 in assembly
PMID: 37627288<br>**2023 Biomolecules – Review**<br>"Wiggle and Shake: Managing and Exploiting Conformational Dynamics during Proteasome Biogenesis"<br>Tomko Laboratory	Blm10 in assembly not mentioned	Not mentioned
PMID: 35740889<br>**2022 Biomolecules – Review**<br>"Allostery Modulates Interactions between Proteasome Core Particles and Regulatory Particles"<br>Cheng Laboratory	Nothing on Blm10 in assembly.	Not mentioned
PMID: 35451017<br>**2022 Journal of Cell Science - Review**<br>"Chaperone-mediated assembly of the proteasome core particle - recent developments and structural insights."<br>Hanna Laboratory	[REDACTED]	No model on Blm10 in assembly
PMID: 35563886<br>**2022 Cells – Review**<br>"The Molecular Mechanisms Governing the Assembly of the Immuno- and Thymoproteasomes in the Presence of Constitutive Proteasomes"<br>Murata Laboratory	[REDACTED]	Blm10 functions after Pba1/2

PMID: 31231659<br>**2019 Front Mol Biosci. – Review**<br>"Dynamic Regulation of the 26S Proteasome: From Synthesis to Degradation"<br>Vierstra Laboratory	[REDACTED]	Blm10 functions after Pba1/2
PMID: 30065390<br>**2018 Nat Rev Mol Cel Biol – Review**<br>"Regulation of proteasome assembly and activity in health and disease"<br>Bertolotti Laboratory	Blm10 in assembly not mentioned	Not mentioned
PMID: 29243089<br>**2018 Biophys Rev – Review**<br>"Structural insights on the dynamics of proteasome formation"<br>Satoh Laboratory	Blm10 in assembly not mentioned	Not mentioned
**2017 Front. Biol – Review**<br>"Putting it all together: intrinsic and extrinsic mechanisms governing proteasome biogenesis"<br>Kusmierczyk & Tomko Laboratory	[REDACTED]	No model on Blm10 in assembly

In fact, the real question is whether Blm10 has any role at all in CP assembly. The authors themselves note in their Introduction that "[i]t is unclear what role Blm10 plays when bound to assembly intermediates…." Unfortunately, this statement is still true following my reading of the results presented here. If the alpha-ring of various intermediates is not bound by Pba1/2, which would clash sterically with Blm10 binding, then the exposed ring surface could simply associate with available Blm10, which has an immense binding surface for binding to the alpha ring, as well as the HbYX motif at its C-terminus. Blm10 might still function entirely as a regulator of mature CP proteolytic activity, as originally proposed by Schmidt and colleagues.

We provide compelling data that Blm10 is not simply a passive binder to an available alpha-ring, as it can bind to 13S assembly intermediates and prevent Pba1/Pba2 from binding. This mechanism by definition creates parallel assembly pathways, unless the reviewer believes the Blm10-13S complex to be a dead-end product that does not result in the formation of Blm10-CP complexes. As we describe above, this possibility is unlikely.

Moreover, as the assembly of proteasomes is complex and highly regulated with several proteasome assembly chaperones actively functioning in preventing premature interactions between complexes (like Hsm3, nas6, Rpn14 and Pba1/2), we find the notion of binding by coincidence not very compelling. Also, the lack of Pba1/2 leads to RP that is normally not detected on immature CP binding CP assembly intermediates. Blm10 clearly behaves differently and binds to immature CP in the presence and absence of Pba1/2, which argues against Blm10 coincidental binding to 13S. Since both Blm10 and Pba1/Pba2 utilize the same alpha5-alpha6 pocket and in the absence of Pba1/Pba2, alpha5/6 might be missing (our structures and Wani et al, 2016 Nat.comm, PMID: 25812915), both of these complexes appear capable of stabilizing the incorporation of alpha5-6.

Our data, however, do not support a parallel pathway of CP assembly through Blm10 simply to make more mature CP. Because newly added phenotypic studies (Extended data Fig. 8 and 9). do not show a more severe phenotypes for *pba1Δ blm10Δ* strains, even in combination with deletion of RPN4 or the $\beta 7^{CTE}$ mutant. Therefore, we favor the model whereby Blm10 association early in CP assembly ensures the formation of Blm10:CP complexes.

It is still poorly understood how the cell regulates the levels of Blm10:CP versus RP:CP. This might be regulated by the expression level of Blm10 together with its ability to interact with CP intermediates during assembly, unlike RP. It has been hard to find a good way to dissect this as we have not identified structural feature that would enable us to disrupt binding of Blm10 only to immature CP. However, there is interesting data that Blm10 has a synergetic phenotype with β7 mutants ($\beta 7^{CTE}$) that interfere with CP assembly. We attempted to explore this in more detail and observed synergistic interactions between Blm10 and β7 that were not exacerbated by a deletion of Pba1. While we cannot dissect if this reflects an assembly or mature Blm10:CP role, we did discover that the initial phenotype is due to a third genetic modifications, namely the lack of a terminator behind the modified β7. From experience with a different proteasome subunit, we know that such a lack of terminator leads to reduced expression levels, which here clearly contributed to the phenotype. We included these new data in the manuscript as they provide additional insight and clarification of previous data as well. While these do not provide an unambiguous answer regarding a role of Blm10 in binding to 13S, they do make a compelling case against a Blm10:13S assembly pathway being available simply to boast CP production; namely, the separate pathways are clearly not substituting for each other. Therefore, we favor a model whereby this parallel pathway helps to ensure the substoichiometric Blm10 subunit form Blm10:CP complexes.

In sum, our manuscript shows the existence of parallel CP assembly pathways, presents this concept in the context of previous literature, and provides some conceptual arguments for its relevance. Future work will surely expand on the mechanisms and functionality of regulators binding to immature CP. This might end up becoming increasingly relevant considering the observed upregulation and relevance of Blm10 in cancer. Also, this concept might be relevant for other regulators of CP, like PA28α/β and PA28γ.

My opinion is that these new structures are fine in and of themselves but add only incrementally to our understanding of CP assembly given the large number of papers on CP assembly intermediates published in the past few years. Neither the data nor the proposed model of parallel Pba1/2 and Blm10 dependent CP assembly pathways resolves whether Blm10 truly has a role in assembly. As such, I would recommend the current manuscript for a more specialist structure journal.

We clearly have a different point of view. We would argue the numerous papers on CP assembly describing in increasing detail aspects of the well-known assembly pathway in high impact journals reflects interest of the field into this pathway and the broader significance of CP assembly. We don't see why these papers, describing completely different structures would make our work incremental. While we realize we might not convince the reviewer of our points of view, we feel the reviewer will be hard-pressed to deny that our paper does not provide a different aspect and view that is not currently represented in the literature on the CP assembly pathway. In addition to providing novel structures and structural insights, we introduce the concept of parallel assembly pathways for CP, and provide a putative mechanism that cells might use to ensure the presence of Blm10-CP as well as RP-CP complexes.

Other comments:

Line 168. While I believe that CP intermediates involving incomplete alpha rings are likely to be on-pathway for CP assembly or at least resolvable into competent assembly intermediates, I think the concluding sentence here needs to be tempered: “Thus, at least in this pba1Δ background, assembly does not always proceed by formation of the a-ring first.” I would add “probably” before “does not”.

Thank you for pointing that out, we agree and have rephrased this sentence.

Line 188: The “closed state” still has a sizeable opening of the Blm10 aperture. Would an unfolded polypeptide chain still be able to thread through it?

Based on comparison to other gated structures, we think this is unlikely. For CP itself, the closed gate, at 9 Å diameter, does not allow for diffusion of LLVY-amc into the CP core. The open CP state on the other hand allows for unfolded polypeptide entry and has a diameter of 20 Å. Another complex that only allows for unfolded peptide movement in the open state is the Sec61 complex, whose pore opening is 40-60 Å in open state and 9-15 Å in closed state. Our reported closed state for the Blm10 dome has a remaining opening of 6.3 by 11 Å, which is similar or smaller the closed examples listed above.

Line 195: “In all, these data suggests that the Blm10 dome opening can change from a closed state to an open state, enabling potential allosteric regulation of Blm10-dependent protein degradation.” There are no data here that directly address effects on Blm10-dependent degradation.

We agree and apologize if we gave the impression that our paper claimed to show Blm10-dependent degradation. There have been a number of papers that conclude their data indicate Blm10-dependent degradation of proteins and postulate the gate of Blm10 is the substrate entry site. We show a state of Blm10 where this opening is closed and infer that Blm10-dependent degradation has the potential to be regulated via this gate. We hope our rephrasing more appropriately reflect our intended conclusion:
“In all, these data suggests that the Blm10 dome opening can change from a closed state to an open state. Such putative allosteric regulation might be relevant as Blm10 has been proposed to degrade specific protein substrates.”

Figure 6b: Looks to me like you pull down the 'other' chaperone as well, e.g., Blm10-TAP pulls down some Pba4 and vice versa. Maybe Blm10 and Pba4 binding is not fully mutually exclusive?

We indeed believe Blm10 and Pba3-Pba4 are not mutually exclusive (nor is there a reason to assume so based on Pba3-4 structures showing it binding on the other side of the alpha ring compared to Pba1/2 or Blm10). The figure in 6b is easy to misinterpret as the second sample has Pba4-TAP tagged and the third sample has Ump1-TAP tagged (no Blm10-TAP tag was used). We use a western against the TAP tag to detect these two proteins in the purification. However, their molecular weights are very close, so lowest band appears to detect the same protein in lane 2 and lane 3; however in lane 2, it recognizes the Tap-tag of Pba4 and in lane 3 the TAP tag of

Ump1. The middle panel shows Pba1/2 purified in both cases, indicating Pba1/2 is present early and late. The top panel shows Blm10 being present early and late.

We do discuss in the manuscript that a recent paper describes a complex that contains Ump1 and Pba3-4, something that was unexpected and not know at the time of our design. So, our early and late stage intermediates might show a small window of overlap. We discussed this caveat in the manuscript, but believe this intermediate is transient and low abundance (hence it was not detected before) and therefore does not impact our conclusion in a meaningful way.

We thank all reviewers for their time and constructive feedback. We are confident our manuscript has substantially improved and believe it will make a valuable contribution to the proteasome assembly literature by providing new structures and structure-based models as well as original concepts regarding Blm10-dependent CP assembly pathway.

Point to point rebuttal on reviewer comments

Reviewer #1 (Remarks to the Author):

We really appreciate all the work the authors put onto the manuscripts and new experiments to address our questions and requests.
The paper, which was already good, has further improved.

We appreciated the feedback from the reviewer in the previous round that helped improve our manuscript and are please all questions have been addressed.

Reviewer #3 (Remarks to the Author):

I co-reviewed this manuscript with one of the reviewers who provided the listed reports. This is part of the Nature Communications initiative to facilitate training in peer review and to provide appropriate recognition for Early Career Researchers who co-review manuscripts.

We appreciated the feedback from the reviewer in the previous round that helped improve our manuscript and are please all questions have been addressed.

Reviewer #4 (Remarks to the Author):

I like the rewriting of the abstract and introduction, and some of the new genetic data are revealing. However, while the structures of Blm10-13S, etc. in the manuscript are fine in and of themselves, I still am not convinced of their physiological function in CP (or Blm10:CP) assembly.

We are happy to hear the reviewer appreciates the improved text, his/her feedback in the previous round was instrumental for that. We have addressed all concerns raised below, including the implications for assembly.

Fig. 1a. Anti-Blm10 blot should have individual species labeled. It is hard to tell which bands are what.

Thank you for the suggestion, labels have been added.

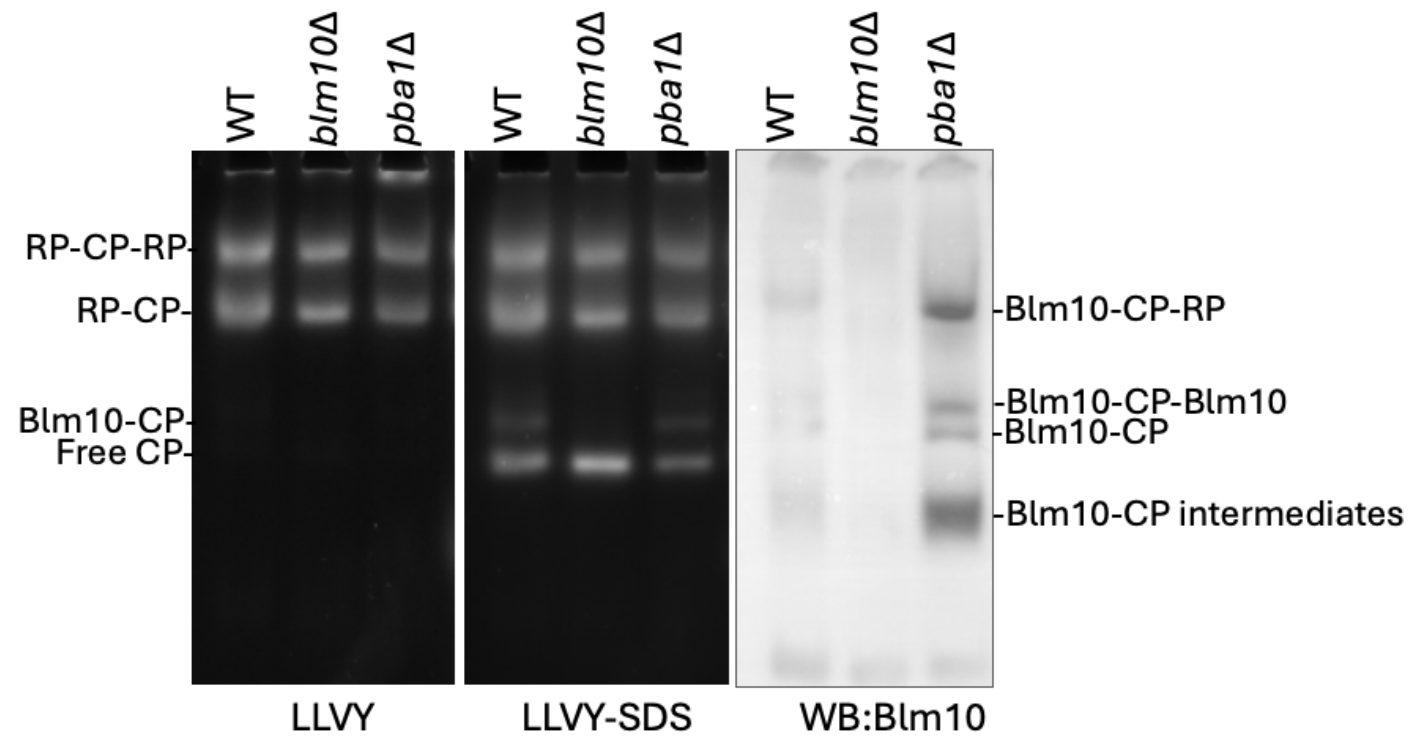

Line 79. The CTE of beta7 is part of the mature protein. It is not a propeptide.

Thank you for noticing that textual error, propeptide is now deleted. To avoid any confusion, please note that "CTE" stands for C-Terminal Extension, which is the mutant described in reference 20 and was used in phenotype assays. It does not refer to the normal endogenous C-terminus.

Line 125. I don't see evidence that the Blm10-CP-RP complexes are proteolytically active, as stated. These particles are not obviously resolved from RP-CP complexes in the substrate overlay assays in these native gels. Consistent with this point, the authors do not mark this active Blm10-CP-RP complex in extended data Fig. 1a.

We feel confident that Blm10-CP-RP complexes form in this assay and the immunoblot for Blm10 best illustrates this. Blm10-CP-RP complexes are present in wild type lysate (lane 1), absent in *blm10Δ* lysate, and reform in the *blm10Δ* lysate that was combined with CP intermediates from *pba1Δ* (lane 3) . We used the red arrow to highlight this. It can be tricky to clearly separate Blm10-CP-RP from CP-RP on native gels and we agree that in this assay that separation is not ideal, which makes the claim of proteolytic activity less compelling. To avoid ambiguity, we deleted proteolytically active from the claim and only focus on the main point that Blm10-CP-RP species are present, which is evident from the immunoblots for Blm10.

Also, these blots should have included an anti-RP antibody to show its inclusion in the larger species (although this is highly likely!)

We acknowledge the reviewer's point and apologies for not including this. However, based on the activity observed without SDS and the migration behavior of RP-CP species in numerous publication from many different labs, we feel the presence of RP in that migration pattern is well established (which is the reason it slipped our mind to blot for it)

and feel confident to argue that its presence is beyond highly likely.

Line 185: "There is no structural indication that 5α3βUmp1 and 5α4βUmp1 represent dead-end products because all subunits are correctly ordered in these intermediates." But I think there is no functional evidence that they are on-pathway intermediates either. True?

While we agree with the reviewer that the structure does not necessarily show that they are on-pathway intermediates, we feel the argument is a bit different; to argue this is an intermediate that is off-pathway requires us to invoke some unknown mechanism that prevents this from being an on-pathway intermediate. There are no clear steric clashes or wrong subunit order that makes it evident. So, we feel it is correct to note there is no structural indication this represents a dead-end product.

Line 244: However, despite this open gate conformation, the complex is unlikely to be catalytically active, as the Blm10 dome is closed" And because the catalytic sites are physically absent!

Thank you for highlighting that, as it did not capture what we intended to say and your reason is indeed more pertinent. We rephrased it to: "However, despite this open gate conformation, the entry of polypeptides from the top is unlikely, as the Blm10 dome is closed."

Line 324. "Thus, both Blm10 and Pba1 are present in early as well as late stages of CP assembly, which is consistent with parallel assembly pathways." Potentially supporting this, a previous report had also seen both Blm10 and Pba1 in 13S-like particles (doi: 10.1038/sj.emboj.7601681.)

Indeed, that is a nice and relevant paper by the Hochstrasser lab. It was not our intension to ignore that, so thank you for pointing out it was missing in our references. We added the reference in this section.

Line 338. What exactly is this beta7-CTE mutant? Should clarify.
The beta7-CTE is a deletion of the last 19 amino acids of β7. We now include this in the text. It was described by the Dohmen group to have a genetic interaction with Blm10 in a 2007 JBC publication (PMID: 1791110) and was described prior to that in a 2004 paper from that group (PMID: 14722099). From the method section, it is hard to retrace the exact genetics that generated the strain and it seems they might have used a selection marker from a plasmid that does not have a specific transcriptional terminator for the targeted

truncated gene. So, if the selection cassette is integrated behind the PRE4 ORF and the endogenous transcriptional terminator is lost, there would be no replacement terminator to compensate for this loss. In that case, the CTE truncation could be a combination of PRE4 truncation AND lower expression levels due to the lack of a transcriptional terminator. Therefore in our phenotype screen, we used +/- truncation and +/- introduction of compensatory transcriptional terminator. We tried to capture this in the image below and the exact primers and plasmids used in our study are in the supplement, so each genetic background can be assessed up to the basepair.

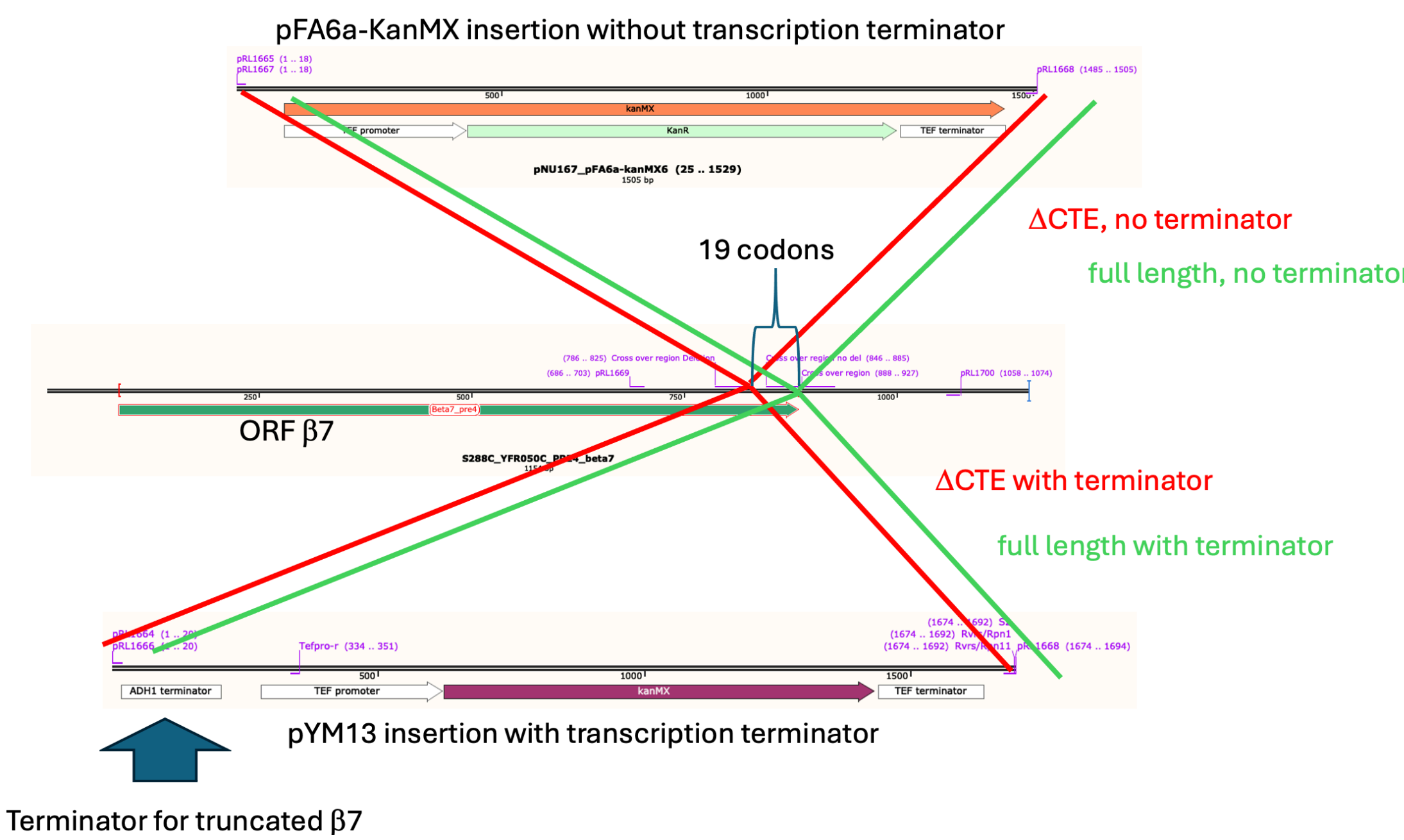

Supplementary Table 3. PCRs for PRE4 integration material and screening

	Primer 1	Primer 2	template	Screening size
PRE4^{CTE}*(KAN)*	pRL1664 (AGA ACA CAG GTT TAA CTT TCA AGA AAA ACT TGC AAG TTT AGC GCC ACT TCT AAA TAA GCG)	pRL1668 (AAA AAT AAA ATG AGT ATT TAA TGA AGG TGG GAT AAA ACT CAT CGA TGA ATT CGA GCT CG)	pYM13 (Janke et al, Yeast, 2004)	
PRE4$^{CTE, no terminator}$ *(KAN)*	pRL1665 (AAG AAC ACA GGT TTA ACT TTC AAG AAA AAC TTG CAA GTT TAG CGT ACG CTG CAG GTC GAC)	pRL1668 (AAA AAT AAA ATG AGT ATT TAA TGA AGG TGG GAT AAA ACT CAT CGA TGA ATT CGA GCT CG)	pFA6a-kanMX6 (Bahler et al, Yeast, 1998)	
PRE4(KAN)	pRL1666 (CGC CAA GGA TAT TAA AGG CTA CGG TAC TCA AAA AAT TTA GCG CCA CTT CTA AAT AAG CG)	pRL1668 (AAA AAT AAA ATG AGT ATT TAA TGA AGG TGG GAT AAA ACT CAT CGA TGA ATT CGA GCT CG)	pYM13 (Janke et al, Yeast, 2004)	
PRE4$^{no terminator}$ *(KAN)*	pRL1667 (CGC CAA GGA TAT TAA AGG CTA CGG TAC TCA AAA AAT TTA GCG TAC GCT GCA GGT CGA C)	pRL1668 (AAA AAT AAA ATG AGT ATT TAA TGA AGG TGG GAT AAA ACT CAT CGA TGA ATT CGA GCT CG)	pFA6a-kanMX6 (Bahler et al, Yeast, 1998)	
**Screening primers** pRL1669 (CCA CTG TAC AGG TTG CTG) pRL1700(AGC GGT GGT GCA TGG TG), and pR714(GCG ACA GTC ACA TCA TGC)				
wild type				389 bp
PRE4^{CTE}*(KAN)*				490 bp
PRE4$^{CTE, no terminator}$ *(KAN)*				302 bp
PRE4(KAN)				550 bp
PRE4$^{no terminator}$ *(KAN)*				362 bp

Line 341. What kind of terminator? Stop codon? Transcriptional terminator?

Thank you for asking for a clarification. We now use the more precise term transcriptional terminator.

Line 343. "Deletion of Pba1 in this background did not further sensitize these cells" Another interpretation is that Blm10 simply does not have a significant effect on CP assembly. Alternatively, the fact that the double pba1 blm10 deletion does not exacerbate the growth defects of the singles could also still argue that they work in the same pathway.

Here we make a conclusion of the phenotype screen without an interpretation as one can see there is no increase sensitivity of those cells. Considering both Pba1 Blm10 binds to assembly intermediates, we think it is a meaningful observation that there was no further sensitization of the cells. The interpretation of this result in the context of all other data and the larger literature is a point for the discussion and something we considered in the models we presented and remains open to be debated.

Line 353. "revealing mutually exclusive binding of Blm10 and Pba1/Pba2 to the 13S" This was pretty much guaranteed to be true given that Blm10 occludes virtually the entire alpha-ring end surface.

Yes and No. Several recent structures of proteasome assembly intermediates have provided unexpected features. For example, the N-terminus of Pba1 going into the CP was not seen when bound to mature CP, but was seen on Pba1-2-13S structures; the Ump1/PI31 binding to CP; the observation of a complex with Ump1, Pba1-2, and Pba3-4 altogether (that was theorized not to exist). So, while we agree that it was within expectation, the data to show how Blm10 binds to immature CP and mature CP provides the data to support this assumption.

Line 368. "…Blm10 or RP might substitute for Pba1/Pba2 function. However, the lack of a synergy phenotype between BLM10 and PBA1 in the rpn4delta or beta7CTE backgrounds, suggest this is not an important function of the Blm10:13S assembly pathway." As above, another interpretation is that Blm10 has a function distinct from CP assembly, and the small fraction of Blm10-13S complexes observed might be physiologically insignificant or involved in some kind of quality control or proteasome nuclear import as others have proposed.

We have not argued against a function of Blm10 distinct from CP assembly, we see no reasons for that to be mutually exclusive. Rather the contrary, we favor a model where the Blm10:13S complexes form to ensure the cell forms Blm10:CP or Blm10:CP:RP complexes

that then would execute a function distinct from CP assembly. Currently it is not well understood how the cell regulated RP:CP versus Blm10:CP complex formation, we argue this might happen (in part) during assembly.

Our discussion we also highlight potential quality control functions (the third putative function mentioned in the discussion, ~ line 397).

In their rebuttal letter, the authors state, based on this lack of genetic interaction (which was not what they had originally predicted), "Therefore, we favor the model whereby Blm10 association early in CP assembly ensures the formation of Blm10:CP complexes." This is possible but assumes slow dissociation of Blm10 from assembly intermediates and Blm10:CP complexes. Is there evidence for this? If Blm10:CP complexes are purified, do they limit RP-CP-RP formation when challenged with RP at relative concentrations that are close to physiological?

That Blm10 binds similar to assembly intermediates and mature CP is something we observed in the past (see references PMID: 25812915 and PMID: 33639167) and is consistent with our structural understanding when comparing Blm10:13S and Blm10:CP structures. This is different from Pba1-Pba2 that binds strongly to 13S and weakly to CP. While we don't have the exact experiment the reviewer is asking for, which we feel is beyond the current rebuttal stage, we would like to highlight three considerations:

(1) In the experiment below on the left, which is unpublished data, we purified 26S proteasomes and then added Blm10 to the samples. The experiment indicates that RP:CP is not extremely dominant and RP does not readily displace Blm10.
(2) Blm10:CP and the Blm10:13S complexes are purified using CP or Ump1-based affinity tags. During the purification and following extensive washing (> 50 bed volumes), Blm10 is found bound to the complexes and not washed away. With a fast dissociation of Blm10, the Blm10 would be lost.
(3) There is some compelling data in PMID: 33639167, shown to the right in the figure below. To our knowledge Blm10 is normally found associated with CP or 13S and not observed as a free species. Only with the overexpression shown below, it is observed as a free species by native gel (after almost all CP binding sites are occupied with Blm10). Blm10 is normally substoichiometric (except in special cells like during sperm development in metazoa) and thus not competed aways by free RP that is normally detectable in cell lysates.

Based on this context, we feel our favored model is reasonable and we look forward to testing it further in the future.

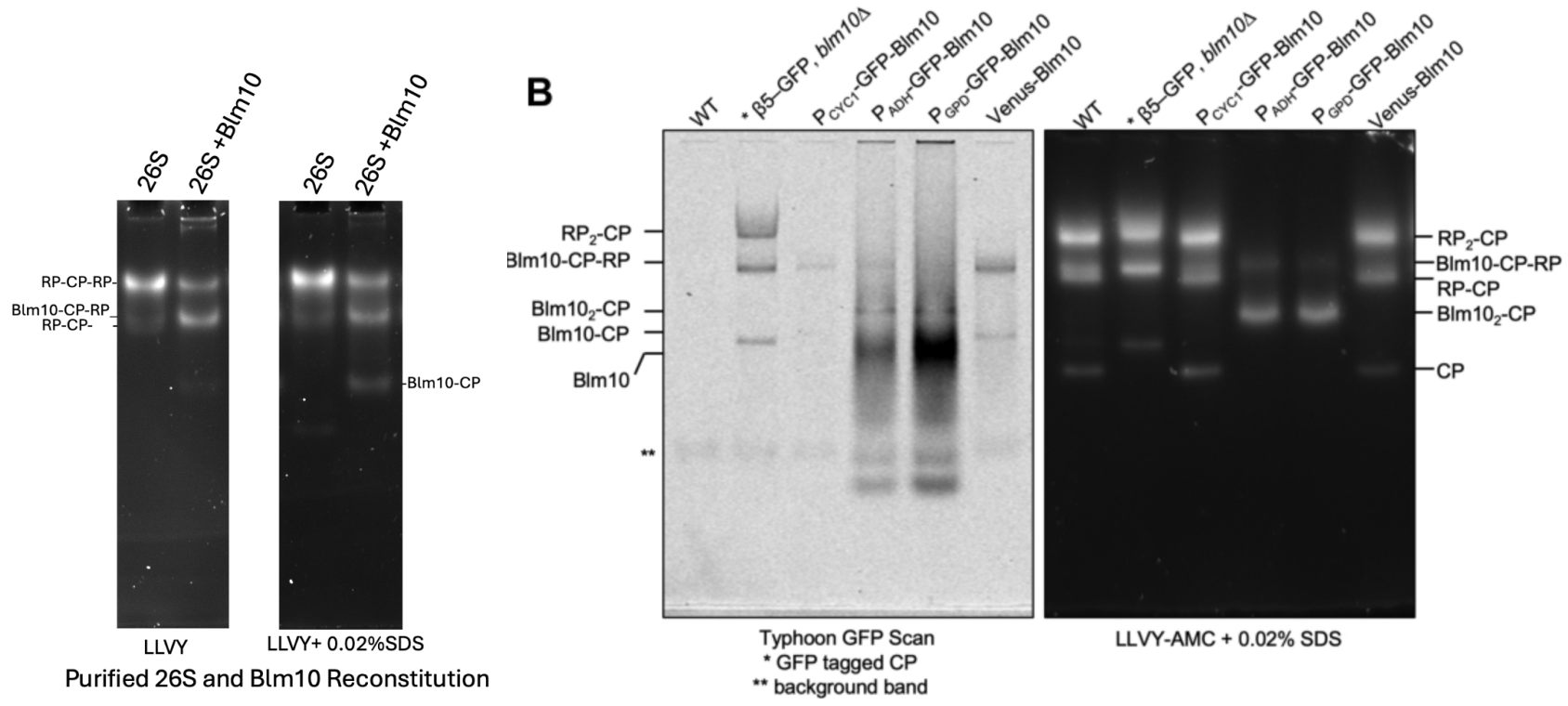

We appreciate the reviewer's critical reading of the manuscript and thoughtful suggestions, and actually think the discussion points raised and incorporated in the discussion are valuable for the field to further test these concepts and ideas raised.
